# Supplementary material for: Enantioselective total syntheses of (+)-stemofoline and three congeners based on a biogenetic hypothesis
Source: Nat Commun. 2020 Oct 20;11:5314. doi: 10.1038/s41467-020-19163-4 (PMC7576163; doi:10.1038/s41467-020-19163-4)
Supplement: Supplementary file 1 — Supplementary Information [file 41467_2020_19163_MOESM1_ESM.pdf]

Supplementary Information

Enantioselective Total Syntheses of (+)-Stemofoline and Three Congeners Based on a Biogenetic Hypothesis

Huang *et al.*

Table of Contents

Eight Structural Groups of *Stemona* Alkaloids 2

General Information 3

Experimental Procedures 4

Comparison of ^{1}H and ^{13}C NMR Data of Our Synthetic Compounds (**1**, **2**, **7**-**9**) with Those of Reported Data 29

NOESY Spectra of Compounds **24**, **13**, **12**, and **31** 38

Single-Crystal X-Ray Diffraction Analysis of **30** 45

NMR Spectra of the Synthesized Compounds in This Article 48

Supplementary References 104

Supplementary Note 1

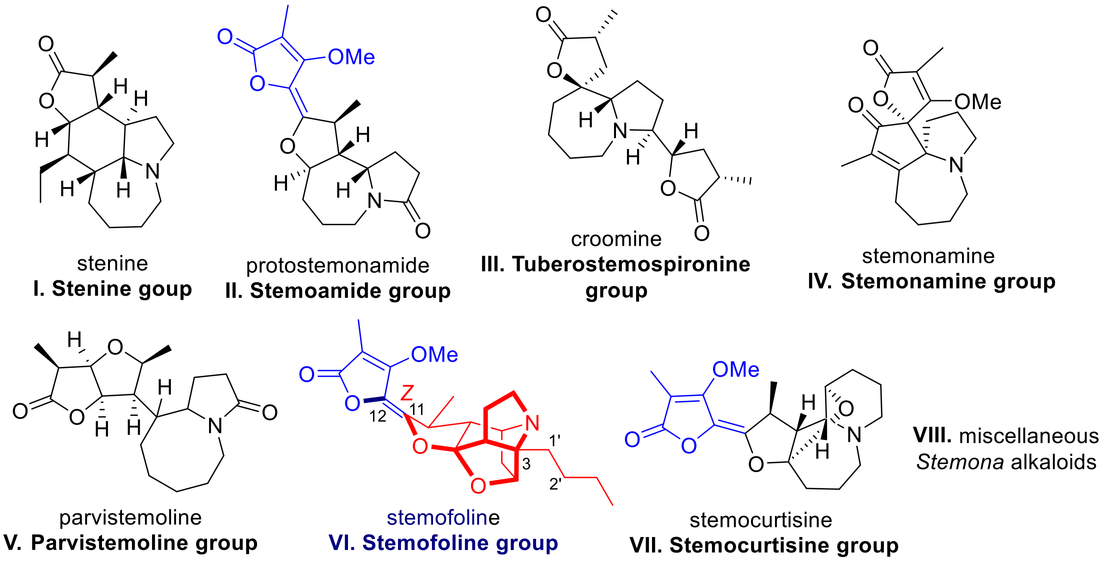

Supplementary Figure 1. Eight structural groups of *Stemona* alkaloids

Supplementary Note 2

General Information

All reactions were carried out under a nitrogen atmosphere under anhydrous conditions and all reagents were purchased from commercial suppliers without further purification. Solvent purification was conducted according to Purification of Laboratory Chemicals (Peerrin, D. D.; Armarego, W. L. and Perrins, D. R., Pergamon Press: Oxford, 1980). Reactions were monitored by Thin Layer Chromatography on plates (GF254) supplied by Yantai Chemicals (China) visualized by ultraviolet or stained with ethanolic solution of phosphomolybdic acid and cerium sulfate or iodine vapour. Flash column chromatography was performed using Qindao Haiyang Chemicals silica gel (200-300 mesh), eluting (unless otherwise stated) with EtOAc/ *n*-hexane (Hex) or EtOAc/ petroleum ether mixture (bp. 60-90 °C) (PE). NMR spectra were recorded on Bruker AV III 400, Bruker AV III 500, Bruker AV III 850 instruments and calibrated by using tetramethyl silane (TMS) (δH = 0.00 p.p.m.) and $CDCl_3$ (δC = 77.00 p.p.m.) as internal references. The following abbreviations were used to explain the multiplicities: s = singlet, d = doublet, t = triplet, q = quartet, br = broad, dd = double doublet, td = triple doublet, dt = double triplet, dq = doubl quartet, m = multiplet. Infrared (IR) spectra were measured with Nicolet FT-380 spectrometer using film KBr pellet techniques. High-resolution mass spectra analyses were performed on Fourier transform ion cyclotron resonance (FT-ICR) mass spectrometer (Bruker Daltonics) with 7 T magent (Magnex) and electrospray ionization (ESI) source (Apollo II, Bruker Daltonics) in positive ion mode. Optical rotations were measured using Anton Paar MCP-500 polarimeter. Melting points were uncorrected.

Supplementary Note 3

Experimental procedures

Synthesis of Compound *cis*-17

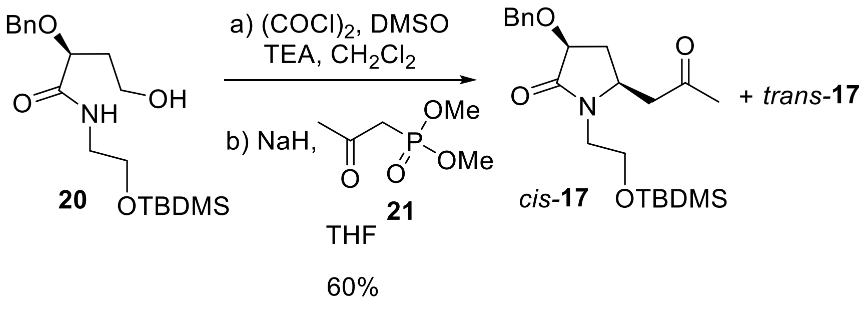

To a cooled solution (–78 ºC) of oxalyl chloride (4.26 mL, 49.7 mmol) in CH_2Cl_2 (110 mL) under a nitrogen atmosphere was added dropwise a solution of DMSO (7.04 mL, 99.4 mmol) in CH_2Cl_2 (23 mL). After being stirred for 15 min at –78 ºC, a solution of compound **20** (16.57 g, 45.2 mmol) (compound **20** was prepared from the addition of commercially available (*S*)-α-benzyloxy-γ-lactone (*S*)-**18** with *O*-silylated β-aminoethanol **19**)[1] in CH_2Cl_2 (45 mL) was added dropwise. After being stirred at the same temperature for 30 min, Et_3N (31.4 mL, 226 mmol) was added. The reaction was stirred for 2.5 h at –78 ºC before being quenched with ice-water (50 mL). The organic layer was separated and the aqueous layer was extracted with CH_2Cl_2 (3 × 200 mL). The combined organic layers were washed with brine, dried over anhydrous Na_2SO_4, filtered, and concentrated under reduced pressure. The presumed mixture of aldehydo amide and its ring-opening tautomer **A** was directly used in the next step without further purification.

To a suspension of dimethyl acetylmethylphosphonate **21** (10.6 mL, 76.8 mmol) in THF (500 mL) at 0 ºC under a nitrogen atmosphere was added NaH (2.71 g, 67.8 mmol, 57-63% oil dispersion) and the mixture was stirred for 1 h. To the resulting mixture was added a solution of mixture **A** in THF (50 mL) at 0 ºC. The reaction mixture was warmed to reflux and stirred overnight. Then the reaction was quenched with a saturated aqueous solution of NH_4Cl (100 mL). The organic layer was separated and

the aqueous layer was extracted with EtOAc (3 × 200 mL). The combined organic layers were washed with brine, dried over anhydrous Na_2SO_4, filtered, and evaporated. The residue was purified by flash chromatography on silica gel (EtOAc/PE = 1/2) to give compound *cis*-**17**[1] (10.96 g, colorless oil, yield: 60% from **20**) and *trans*-**17**[1] (3.85 g, colorless oil, yield: 21% from **20**).

Compound *cis*-**17**: $[\alpha]_D^{20}$ –49.5 (*c* 1.0, $CHCl_3$); **IR** (film) ν_{max}: 2953, 2929, 2857, 1697, 1454, 1421, 1376, 1255, 1098, 1027, 836, 778 cm^{-1}; **^{1}H NMR** (400 MHz, $CDCl_3$) δ 7.41–7.25 (m, 5H), 4.92 (d, *J* = 11.9 Hz, 1H), 4.72 (d, *J* = 11.9 Hz, 1H), 4.08–4.00 (m, 2H), 3.78–3.62 (m, 3H), 3.14 (dd, *J* = 17.6, 3.7 Hz, 1H), 3.11–3.05 (m, 1H), 2.62 (dd, *J* = 17.6, 9.8 Hz, 1H), 2.55 (dt, *J* = 13.7, 7.6 Hz, 1H), 2.14 (s, 3H), 1.63 (dt, *J* = 13.7, 5.0 Hz, 1H), 0.87 (s, 9H), 0.04 (d, *J* = 2.5 Hz, 6H); **^{13}C NMR** (100 MHz, $CDCl_3$) δ 206.1, 172.8, 137.9, 128.3 (2C), 127.9 (2C), 127.7, 75.4, 72.0, 61.3, 52.5, 48.1, 43.5, 33.6, 30.6, 25.8 (3C), 18.1, −5.5 (2C); **HRMS** calcd for $C_{22}H_{35}NO_4SiNa$ [$M+Na^+$]: 428.2228; found: 428.2229.

Compound *trans*-**17**: $[\alpha]_D^{20}$ –57.5 (*c* 1.0, $CHCl_3$); **IR** (film) ν_{max}: 2950, 2928, 2857, 1715, 1459, 1253, 1098, 836, 775 cm^{-1}; **^{1}H NMR** (400 MHz, $CDCl_3$) δ 7.40–7.24 (m, 5H), 4.94 (d, *J* = 12.0 Hz, 1H), 4.74 (d, *J* = 12.0 Hz, 1H), 4.23–4.16 (m, 1H), 4.13 (t, *J* = 7.6 Hz, 1H), 3.80–3.70 (m, 2H), 3.62 (dt, *J* = 14.3, 4.8 Hz, 1H), 3.13–3.05 (m, 1H), 3.01 (dd, *J* = 17.4, 3.4 Hz, 1H), 2.41 (dd, *J* = 17.4, 9.6 Hz, 1H), 2.33–2.24 (m, 1H), 2.14 (s, 3H), 1.96 (ddd, *J* = 13.5, 7.6, 3.4 Hz, 1H), 0.88 (s, 9H), 0.05 (s, 6H); **^{13}C NMR** (100 MHz, $CDCl_3$) δ 205.7, 172.9, 137.9, 128.3 (2C), 127.9 (2C), 127.6, 74.5, 71.9, 61.4, 52.4, 47.1, 43.6, 33.4, 30.5, 25.8 (3C), 18.2, −5.47, −5.52; **HRMS** calcd for $C_{22}H_{35}NO_4SiNa$ [$M+Na^+$]: 428.2228; found: 428.2232.

Synthesis of Compound 16

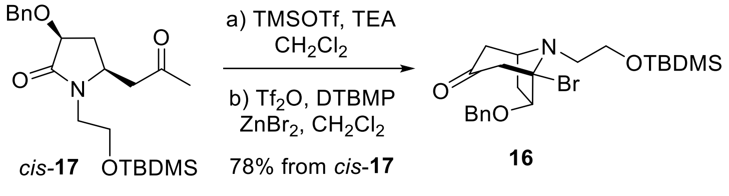

To a cooled solution (0 °C) of compound *cis*-**17** (4.98 g, 12.28 mmol) in anhydrous

CH_2Cl_2 (125 mL) under a nitrogen atmosphere were added Et_3N (4.27 mL, 30.7 mmol) and trimethylsilyl trifluoromethane sulfonate (TMSOTf) (4.44 mL, 24.56 mmol). After being stirred at 0 ºC for 2 h, the reaction mixture was quenched with a saturated aqueous solution of $NaHCO_3$ (30 mL) and extracted with CH_2Cl_2 (3 × 50 mL). The combined organic layers were washed with brine, dried over anhydrous Na_2SO_4, filtered, and evaporated to give an orange residue B, which was used in the next step without further purification.

To a cooled suspension (−78 ºC) of the crude silyl enol ether B, 2,6-di-*tert*-butyl-4-methylpyridine (5.03 g, 24.56 mmol) and $ZnBr_2$ (5.53 g, 24.56 mmol) in anhydrous CH_2Cl_2 (250 mL) was added dropwise trifluoromethanesulfonic anhydride (Tf_2O) (2.43 mL, 14.73 mmol). After being stirred at −78 ºC for 45 min, the mixture was warmed to room temperature slowly and then stirred for 1 h. The reaction was quenched with a saturated aqueous solution of $NaHCO_3$ (50 mL) and extracted with CH_2Cl_2 (3 × 100 mL). The combined organic layers were washed with brine, dried over anhydrous Na_2SO_4, filtered, and concentrated under reduced pressure. The residue was purified by flash chromatography on silica gel (eluent: EtOAc/PE = 1/10) to give bromide **16** (4.51 g, yield: 78% from *cis*-**17**) as a colorless oil.

Compound **16**: $[\alpha]_D^{20}$ −53.5 (*c* 1.0, $CHCl_3$); **IR** (film) ν_{max}: 2951, 2926, 2854, 1722, 1597, 1461, 1404, 1254, 1190, 1098, 893, 835, 775, 730, 693 cm^{-1}; **^{1}H NMR** (400 MHz, $CDCl_3$) δ 7.40–7.25 (m, 5H), 4.78 (d, *J* = 12.2 Hz, 1H), 4.68 (d, *J* = 12.2 Hz, 1H), 4.48 (dd, *J* = 10.4, 3.3 Hz, 1H), 3.90–3.76 (m, 3H), 3.34 (dt, *J* = 13.2, 5.0 Hz, 1H), 3.27 (d, *J* = 16.4 Hz, 1H), 2.95 (d, *J* = 16.4 Hz, 1H), 2.90–2.79 (m, 2H), 2.52–2.43 (m, 1H), 2.15 (d, *J* = 17.3 Hz, 1H), 1.36 (dd, *J* = 13.5, 3.3 Hz, 1H), 0.90 (s, 9H), 0.08 (s, 6H) ppm; **^{13}C NMR** (100 MHz, $CDCl_3$) δ 204.5, 137.6, 128.3 (2C), 127.6, 127.4 (2C), 86.4, 85.4, 72.3, 63.1, 53.2, 50.1, 45.5, 40.9, 35.8, 25.8 (3C), 18.1, −5.45, −5.49 ppm; **HRMS** calcd for $C_{22}H_{34}BrNO_3SiNa$ [M+Na$^+$]: 490.1389 and 492.1369; found: 490.1381 and 492.1370.

Synthesis of compound 15

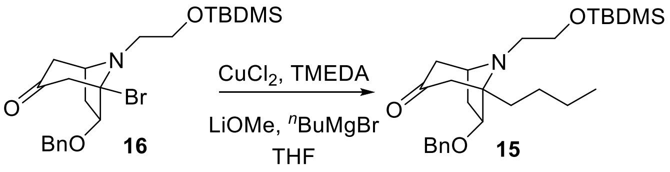

To a solution of the bromide **16** (2342 mg, 5.00 mmol), $CuCl_2$ (134 mg, 1.00 mmol), and LiOMe (228 mg, 6.00 mmol) in anhydrous THF (100 mL) under a nitrogen atmosphere was added *N*,*N*,*N'*,*N'*-tetramethylethylenediamine (TMEDA) (0.32 mL, 2.00 mmol). After being stirred for 10 min, *n*-BuMgBr (14.7 mL, 12.5 mmol, 0.85 M in THF) was added dropwise, and the mixture was stirred for 30 min. The reaction was quenched with a saturated aqueous solution of NH_4Cl (50 mL), and the resulting mixture was stirred for 15 min under air until the aqueous phase turned blue. The mixture was extracted with EtOAc (3 × 100 mL). The combined organic layers were washed with brine, dried over anhydrous Na_2SO_4, filtered, and concentrated under reduced pressure. The residue was purified by flash chromatography on silica gel (eluent: EtOAc/PE = 1/10) to give compound **15** (1810 mg, yield: 81%) as a colorless oil.

Compound **15**: $[\alpha]_D^{20}$ +10.5 (*c* 1.0, $CHCl_3$); **IR** (film) ν_{max}: 2931, 2857, 1716, 1426, 1359, 1256, 1059, 835, 777, 697 cm^{-1}; **^{1}H NMR** (500 MHz, $CDCl_3$) δ 7.34–7.22 (m, 5H), 4.56 (d, *J* = 12.1 Hz, 1H), 4.34 (d, *J* = 12.1 Hz, 1H), 3.80 (dd, *J* = 9.8, 3.5 Hz, 1H), 3.76–3.72 (m, 3H), 2.82–2.66 (m, 3H), 2.60 (d, *J* = 16.0 Hz, 1H), 2.32–2.23 (m, 1H), 2.10 (d, *J* = 16.4 Hz, 1H), 2.04 (d, *J* = 16.0 Hz, 1H), 1.55–1.48 (m, 1H), 1.39–1.31 (m, 2H), 1.28–1.19 (m, 3H), 1.14–1.06 (m, 1H), 0.89 (s, 9H), 0.85 (t, *J* = 7.0 Hz, 3H), 0.06 (s, 6H) ppm; **^{13}C NMR** (125 MHz, $CDCl_3$) δ 209.0, 138.4, 128.2 (2C), 127.4 (3C), 80.4, 71.3, 67.1, 63.0, 55.4, 45.0, 44.0, 41.8, 35.6, 35.4, 25.9 (3C), 24.9, 23.3, 18.3, 14.0, −5.39, −5.41 ppm; **HRMS** calcd for $C_{26}H_{43}NO_3SiNa$ $[M+Na^+]$: 468.2904; found: 468.2905.

General Procedure for Synthesis of functionalized tropinone derivatives 15a-d

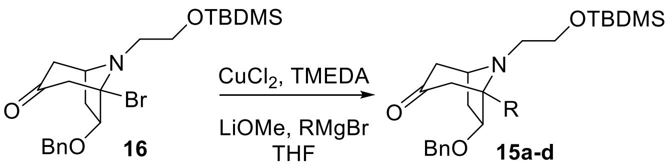

To a solution of the bromide **16** (0.30 mmol, 1.0 equiv), $CuCl_2$ (0.03 mmol, 0.10 equiv), and LiOMe (0.36 mmol, 1.2 equiv) in anhydrous THF (10 mL) under a nitrogen atmosphere was added *N*,*N*,*N'*,*N'*-tetramethylethylenediamine (TMEDA) (0.06 mmol, 0.20 equiv). After being stirred for 10 min, an alkyl bromide (0.75 mmol, 2.5 equiv) was added dropwise, and the mixture was stirred for 30 min. The reaction was quenched with a saturated aqueous solution of NH_4Cl (5 mL), the resulting mixture was stirred for 15 min under air until the aqueous phase turned blue. The mixture was extracted with EtOAc (3 × 30 mL). The combined organic layers were washed with brine, dried over anhydrous Na_2SO_4, filtered, and concentrated under reduced pressure. The residue was purified by flash chromatography on silica gel (eluent: EtOAc/PE = 1/10) to give an alkylated product **15a-d**.

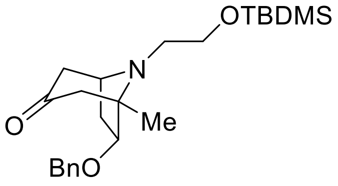

Compound **15a**: (colorless oil, yield: 76%); $[\alpha]_D^{20}$ −2.7 (*c* 1.0, $CHCl_3$); **IR** (film) ν_{max}: 2955, 2929, 2856, 1716, 1426, 1256, 1100, 835, 777, 697 cm^{-1}; **^{1}H NMR** (500 MHz, $CDCl_3$) δ 7.35–7.24 (m, 5H), 4.57 (d, *J* = 12.1 Hz, 1H), 4.39 (d, *J* = 12.1 Hz, 1H), 3.77–3.72 (m, 3H), 3.65 (dd, *J* = 9.8, 3.8 Hz, 1H), 2.79 (dd, *J* = 6.3, 6.3 Hz, 2H), 2.75 (dd, *J* = 16.3, 3.8 Hz, 1H), 2.57 (dd, *J* = 16.3, 1.2 Hz, 1H), 2.38–2.29 (m, 1H), 2.12 (d, *J* = 16.3 Hz, 1H), 2.08 (d, *J* = 16.3 Hz, 1H), 1.33 (dd, *J* = 13.6, 3.8 Hz, 1H), 1.17 (s, 3H), 0.89 (s, 9H), 0.06 (s, 6H) ppm; **^{13}C NMR** (125 MHz, $CDCl_3$) δ 208.9, 138.4, 128.3 (2C), 127.4, 127.3 (2C), 84.4, 71.7, 64.8, 63.4, 55.9, 45.4, 44.1, 41.7, 35.4, 25.9 (3C), 23.6, 18.3, −5.37, −5.40 ppm; **HRMS** calcd for $C_{23}H_{37}NO_3SiNa$ $[M+Na^+]$: 426.2435; found: 426.2440.

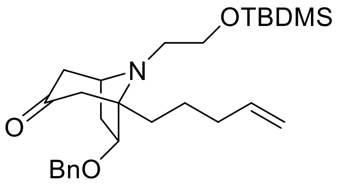

Compound **15b**: (colorless oil, yield: 75%); $[\alpha]_D^{20}$ +13.5 (*c* 1.0, $CHCl_3$); **IR** (film) ν_{max}: 3065, 2930, 2857, 1716, 1640, 1471, 1257, 1100, 835, 777, 738, 698 cm^{-1}; **^{1}H NMR** (500 MHz, $CDCl_3$) δ 7.34–7.24 (m, 5H), 5.71 (dddd, *J* =16.9, 10.1, 6.8, 6.8 Hz, 1H), 5.00–4.92 (m, 2H), 4.57 (d, *J* = 12.1 Hz, 1H), 4.33 (d, *J* = 12.1 Hz, 1H), 3.79 (dd, *J* = 9.7, 3.3 Hz, 1H), 3.77–3.72 (m, 3H), 2.79 (dd, *J* = 14.1, 3.3 Hz, 1H), 2.75 (dt, *J* = 12.7, 6.4 Hz, 1H), 2.69 (dt, *J* = 12.7, 6.0 Hz, 1H), 2.60 (d, *J* = 16.0 Hz, 1H), 2.32–2.24 (m, 1H), 2.11 (d, *J* = 16.6 Hz, 1H), 2.04 (d, *J* = 16.0 Hz, 1H), 1.98 (d, *J* = 6.8 Hz, 1H), 1.95 (d, *J* = 6.8 Hz, 1H), 1.56–1.48 (m, 1H), 1.41–1.32 (m, 3H), 1.28–1.20 (m, 1H) 0.89 (s, 9H), 0.06 (s, 6H) ppm; **^{13}C NMR** (125 MHz, $CDCl_3$) δ 208.9, 138.4, 138.3, 128.3 (2C), 127.5, 127.4 (2C), 114.8, 80.4, 71.3, 67.1, 63.0, 55.4, 45.0, 44.0, 41.8, 35.4, 35.3, 34.1, 25.9 (3C), 22.1, 18.3, −5.36, −5.38 ppm; **HRMS** calcd for $C_{27}H_{43}NO_3SiNa$ [M+Na$^+$]: 480.2904; found: 480.2905.

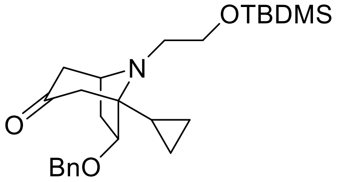

Compound **15c**: (colorless oil, yield: 78%); $[\alpha]_D^{20}$ −5.3 (*c* 1.0, $CHCl_3$); **IR** (film) ν_{max}: 2954, 2928, 2856, 1717, 1462, 1360, 1255, 1098, 835, 777, 697 cm^{-1}; **^{1}H NMR** (500 MHz, $CDCl_3$) δ 7.34–7.23 (m, 5H), 4.54 (d, *J* = 12.2 Hz, 1H), 4.38 (d, *J* = 12.2 Hz, 1H), 3.83–3.72 (m, 3H), 3.67 (dd, *J* = 8.8, 3.5 Hz, 1H), 3.08 (ddd, *J* = 12.7, 6.6, 6.3 Hz, 1H), 2.82 (dd, *J* = 13.3, 6.6 Hz, 1H), 2.80–2.74 (m, 1H), 2.37–2.30 (m, 1H), 2.29 (dd, *J* = 15.8, 1.5 Hz, 1H), 2.10 (dt, *J* = 16.4, 1.5 Hz, 1H), 1.93 (d, *J* = 15.8 Hz, 1H), 1.38–1.32 (m, 2H), 0.90 (s, 9H), 0.83–0.76 (m, 1H), 0.50–0.44 (m, 1H), 0.37–0.30 (m, 2H), 0.07 (s, 6H) ppm; **^{13}C NMR** (125 MHz, $CDCl_3$) δ 208.8, 138.4, 128.2 (2C), 127.3 (3C), 82.9, 71.5, 68.8, 63.5, 55.8, 45.9, 42.1, 39.4, 35.7, 25.9 (3C), 18.3, 17.3, −1.1, −1.0, −5.35, −5.36 ppm; **HRMS** calcd for $C_{25}H_{39}NO_3SiNa$ [M+Na$^+$]: 452.2591; found:

452.2594.

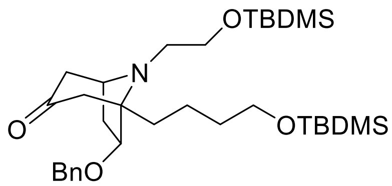

Compound **15d**: (colorless oil, yield: 85%); $[\alpha]_D^{20}$ +5.7 (*c* 1.0, $CHCl_3$); **IR** (film) ν_{max}: 2953, 2929, 2857, 1716, 1463, 1361, 1256, 1100, 836, 776, 698 cm^{-1}; **^{1}H NMR** (500 MHz, $CDCl_3$) δ 7.36–7.24 (m, 5H), 4.57 (d, *J* = 12.1 Hz, 1H), 4.37 (d, *J* = 12.1 Hz, 1H), 3.82 (dd, *J* = 9.8, 3.5 Hz, 1H), 3.79–3.75 (m, 3H), 3.57 (dd, *J* = 6.5, 6.5 Hz, 2H), 2.85–2.69 (m, 3H), 2.63 (d, *J* = 16.1 Hz, 1H), 2.34–2.25 (m, 1H), 2.12 (d, *J* = 16.5 Hz, 1H), 2.06 (d, *J* = 16.1 Hz, 1H), 1.57 (ddd, *J* = 13.8, 12.3, 4.6 Hz, 1H), 1.50–1.42 (m, 2H), 1.42–1.32 (m, 3H), 1.25–1.18 (m, 1H), 0.92 (s, 9H), 0.91 (s, 9H), 0.08 (s, 6H), 0.05 (s, 6H) ppm; **^{13}C NMR** (125 MHz, $CDCl_3$) δ 208.9, 138.4, 128.2 (2C), 127.4, 127.3 (2C), 80.7, 71.4, 67.1, 63.2, 62.9, 55.4, 45.0, 43.9, 41.8, 35.9, 35.5, 33.4, 26.0 (3C), 25.9 (3C), 19.2, 18.30, 18.26, −5.3 (2C), −5.38, −5.41 ppm; **HRMS** calcd for $C_{32}H_{57}NO_4Si_2Na$ $[M+Na^+]$: 598.3718; found: 598.3724.

Asymmetric Total Synthesis of Stemofoline alkaloids (+)-Stemoburkilline (7), (+)-(11*S*,12*R*)-Dihydrostemofoline (8), and unnatural diastereomer (+)-(11*S*,12*S*)-Dihydrostemofoline (9)

Synthesis of Compound 23-1 and 23-2

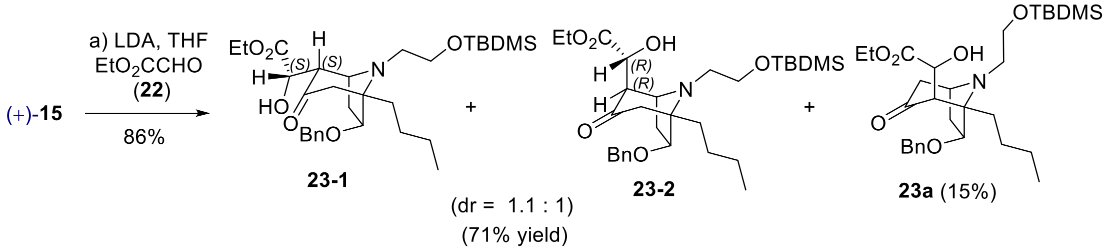

To a cooled solution (−78 °C) of lithium diisopropylamide (LDA) (3.54 mL, 7.08 mmol, 2.0 M in THF) was added a solution of compound **15** (1050 mg, 2.36 mmol) in anhydrous THF (50 mL) under a nitrogen atmosphere. After being stirred at the same

temperature for 30 min, ethyl glyoxylate **22** (1.47 mL, 7.08 mmol, *ca.* 50% in toluene, *v/v*) was slowly added, and then mixture was stirred for 1.5 h at the same temperature. The reaction was quenched with a saturated aqueous solution of NH_4Cl (20 mL). The organic layer was separated and the aqueous layer was extracted with EtOAc (3 × 50 mL). The combined organic layers were washed with brine, dried over anhydrous Na_2SO_4, filtered, and concentrated under reduced pressure. The residue was purified by flash chromatography on silica gel (EtOAc/PE = 1/5) to give two diastereomers **23-1** (475 mg, colorless oil, yield: 37%) and **23-2** (440 mg, colorless oil, yield: 34%) as well as with regioisomer **23a** (single diastereomer, 193 mg, colorless oil, yield: 15%). The diastereomeric mixture **23-1** and **23-2** was used in the next step without separation.

Compound **23-1**: $[\alpha]_D^{20}$ −3.3 (*c* 1.0, $CHCl_3$); **IR** (film) ν_{max}: 3508, 2955, 2930, 2857, 1738, 1714, 1463, 1257, 1009, 936, 836, 778, 737, 698 cm^{-1}; **^{1}H NMR** (500 MHz, $CDCl_3$) δ 7.34–7.23 (m, 5H), 4.57 (d, *J* = 12.2 Hz, 1H), 4.32 (d, *J* = 12.2 Hz, 1H), 4.30–4.20 (m, 2H), 4.11–4.07 (m, 1H), 3.81–3.72 (m, 4H), 3.45 (d, *J* = 7.0 Hz, 1H), 3.34–3.31 (m, 1H), 2.84 (dt, *J* = 13.0, 6.3 Hz, 1H), 2.74 (dt, *J* = 13.0, 6.6 Hz, 1H), 2.62 (d, *J* = 15.5 Hz, 1H), 2.20–2.14 (m, 1H), 2.07 (dd, *J* = 13.8, 3.4 Hz, 1H), 2.04 (d, *J* = 15.5 Hz, 1H), 1.55–1.48 (m, 1H), 1.36–1.31 (m, 1H), 1.29 (t, *J* = 7.1 Hz, 3H), 1.30–1.26 (m, 1H), 1.26–1.20 (m, 2H), 1.12–1.07 (m, 1H), 0.90 (s, 9H), 0.85 (t, *J* = 7.2 Hz, 3H), 0.07 (s, 6H) ppm; **^{13}C NMR** (125 MHz, $CDCl_3$) δ 208.7, 173.0, 138.4, 128.3 (2C), 127.4 (3C), 80.4, 71.5, 70.2, 68.2, 63.1, 61.7, 60.0, 50.6, 44.8, 44.2, 35.3, 32.3, 25.9 (3C), 25.0, 23.3, 18.4, 14.1, 13.9, −5.3 (2C) ppm; **HRMS** calcd for $C_{30}H_{49}NO_6SiNa$ [$M+Na^+$]: 570.3221; found: 570.3228.

Compound **23-2**: $[\alpha]_D^{20}$ +2.4 (*c* 1.0, $CHCl_3$); **IR** (film) ν_{max}: 3460, 2929, 2857, 1714, 1463, 1258, 1098, 1029, 856, 778, 698 cm^{-1}; **^{1}H NMR** (500 MHz, $CDCl_3$) δ 7.34–7.23 (m, 5H), 4.79 (t, *J* = 4.9 Hz, 1H), 4.57 (d, *J* = 12.2 Hz, 1H), 4.30 (d, *J* = 12.2 Hz, 1H), 4.26 (q, *J* = 7.2 Hz, 2H), 3.78 (dd, *J* = 9.6, 3.5 Hz, 1H), 3.73–3.65 (m, 2H), 3.61 (dd, *J* = 7.3, 3.5 Hz, 1H), 3.22–3.18 (m, 1H), 2.82–2.71 (m, 2H), 2.68 (d, *J* = 4.9 Hz, 1H), 2.65 (d, *J* = 15.2 Hz, 1H), 2.20–2.12 (m, 1H), 2.09 (d, *J* = 15.2 Hz, 1H), 1.87 (dd, *J* = 13.9, 3.5 Hz, 1H), 1.56–1.48 (m, 1H), 1.40–1.34 (m, 1H), 1.31 (t, *J* = 7.2 Hz, 3H), 1.26–1.20 (m, 3H), 1.13–1.04 (m, 1H), 0.90 (s, 9H), 0.85 (t, *J* = 7.1 Hz, 3H), 0.06 (s,

6H) ppm; **^{13}C NMR** (125 MHz, $CDCl_3$) δ 207.0, 173.2, 138.4, 128.2 (2C), 127.4 (3C), 80.4, 71.2, 68.6, 68.3, 62.4, 61.7, 57.4, 50.5, 45.1, 44.2, 35.4, 32.6, 25.9 (3C), 25.0, 23.2, 18.4, 14.2, 14.0, −5.31, −5.33 ppm; **HRMS** calcd for $C_{30}H_{49}NO_6SiNa$ [M+Na$^+$]: 570.3221; found: 570.3223.

Compound **23a**: $[\alpha]_D^{20}$ +1.1 (*c* 1.0, $CHCl_3$); **IR** (film) ν_{max}: 3490, 2956, 2929, 2857, 1721, 1463, 1258, 1098, 1029, 854, 777, 698 cm^{-1}; **^{1}H NMR** (500 MHz, $CDCl_3$) δ 7.35–7.23 (m, 5H), 4.61–4.56 (m, 2H), 4.35 (d, *J* = 12.0 Hz, 1H), 4.28–4.20 (m, 2H), 3.88 (dd, *J* = 9.2, 2.2 Hz, 1H), 3.79–3.68 (m, 3H), 2.27–2.25 (m, 1H), 2.98 (dt, *J* = 12.5, 6.4 Hz, 1H), 2.84–2.77 (m, 1H), 2.47–2.36 (m, 3H), 1.97–1.89 (m, 1H), 1.65–1.56 (m, 2H), 1.31 (t, *J* = 7.3 Hz, 3H), 1.31–1.24 (m, 5H), 0.90-0.86 (m, 12H), 0.06 (s, 6H) ppm; **^{13}C NMR** (125 MHz, $CDCl_3$) δ 207.0, 172.3, 137.5, 128.3 (2C), 127.7, 127.6 (2C), 84.4, 72.1, 71.0, 70.9, 63.0, 61.1, 57.3, 54.0, 49.6, 48.7, 34.8, 33.7, 25.9 (3C), 25.5, 23.4, 18.3, 14.2, 13.8, −5.43, −5.45 ppm; HRMS calcd for $C_{30}H_{49}NO_6SiNa$ [M+Na$^+$]: 570.3221; found: 570.3230.

Synthesis of Compound 24

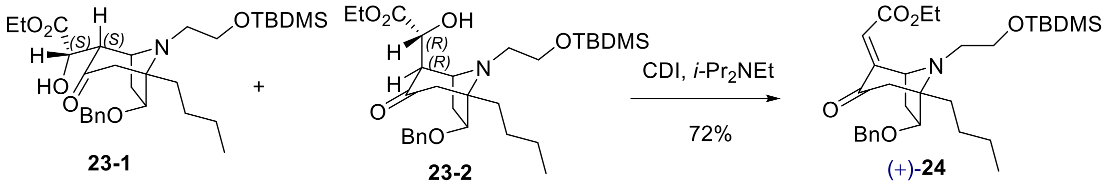

To a cooled solution (0 ºC) of diastereomeric **23-1**and **23-2** (1090 mg, 2.00 mmol) in anhydrous CH_2Cl_2 (40 mL) were added successively *N*, *N*'-4-(dimethylamino)pyridine (DMAP) (244 mg, 2.00 mmol), *i*-Pr_2NEt (2.31 mL, 14.0 mmol), and a solution of *N*,*N*'-carbonyldiimidazole (CDI) (1620 mg, 10.0 mmol) in CH_2Cl_2 (10 mL) under a nitrogen atmosphere. After being stirred for 4 h, the reaction was quenched with a saturated aqueous solution of $NaHCO_3$ (20 mL). The organic layer was separated and the aqueous layer was extracted with CH_2Cl_2 (3 × 50 mL). The combined organic layers were washed with brine, dried over anhydrous Na_2SO_4, filtered, and concentrated under reduced pressure. The residue was purified by flash chromatography on silica gel (EtOAc/PE = 1/10) to give compound **24** (762 mg, yield: 72%) as a colorless oil.

Compound **24**: $[\alpha]_D^{20}$ +85.5 (*c* 1.0, $CHCl_3$); **IR** (film) ν_{max}: 2953, 2924, 2847, 1700, 1460, 1367, 1344, 1242, 1181, 1098, 1027, 832, 775, 733, 698 cm^{-1}; **^{1}H NMR** (500 MHz, $CDCl_3$) δ 7.34–7.22 (m, 5H), 6.60 (s, 1H), 5.20 (d, *J* = 7.7 Hz, 1H), 4.55 (d, *J* = 12.0 Hz, 1H), 4.35 (d, *J* = 12.0 Hz, 1H), 4.21 (dq, *J* = 11.0, 7.2 Hz, 1H), 4.18 (dq, *J* = 11.0, 7.2 Hz, 1H), 3.91 (dd, *J* = 9.2, 2.7 Hz, 1H), 3.71 (ddd, *J* = 9.9, 8.8, 5.3 Hz, 1H), 3.64 (ddd, *J* = 9.9, 8.1, 6.5 Hz, 1H), 2.95 (d, *J* = 18.1 Hz, 1H), 2.72 (ddd, *J* = 15.1, 8.8, 6.5 Hz, 1H), 2.53–2.44 (m, 2H), 2.21 (d, *J* = 18.1 Hz, 1H), 1.60–1.54 (m, 1H), 1.51 (dd, *J* = 13.8, 2.7 Hz, 1H), 1.42–1.34 (m, 1H), 1.30 (t, *J* = 7.2 Hz, 3H), 1.29–1.23 (m, 3H), 1.16–1.08 (m, 1H), 0.88 (s, 9H), 0.86 (t, *J* = 7.2 Hz, 3H), 0.03 (s, 6H) ppm; **^{13}C NMR** (125 MHz, $CDCl_3$) δ 198.8, 165.9, 152.3, 138.1, 128.3 (2C), 127.5, 127.4 (2C), 122.2, 80.5, 71.4, 65.8, 62.2, 60.7, 56.5, 46.0, 43.0, 36.14, 36.11, 25.9 (3C), 25.0, 23.2, 18.4, 14.1, 14.0, −5.4 (2C) ppm; **HRMS** calcd for $C_{30}H_{47}NO_5SiNa$ $[M+Na^+]$: 552.3116; found: 552.3119.

Synthesis of Compound 25

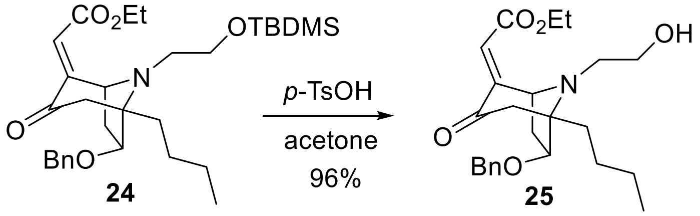

A solution of compound **24** (2540 mg, 4.80 mmol) and *p*-toluenesulfonic acid (PTSA) monohydrate (3650 mg, 19.20 mmol) in acetone (50 mL) was stirred at 50 ºC for 30 min. To the resulting mixture was added a saturated aqueous solution of $NaHCO_3$ till pH reach 8. The organic layer was separated and the aqueous phase was extracted with EtOAc (3 × 100 mL). The combined organic layers were washed with brine, dried over anhydrous Na_2SO_4, filtered, and concentrated under reduced pressure. The residue was purified by flash chromatography on silica gel (EtOAc/PE = 1/1) to give alcohol **25** (1910 mg, yield: 96%) as a colorless oil.

Compound **25**: $[\alpha]_D^{20}$ +78.3 (*c* 1.0, $CHCl_3$); **IR** (film) ν_{max}: 3477, 3029, 2956, 2930, 2862, 1698, 1369, 1346, 1237, 1181, 1098, 1028, 882, 737, 698 cm^{-1}; **^{1}H NMR** (500 MHz, $CDCl_3$) δ 7.35–7.23 (m, 5H), 6.61 (s, 1H), 5.25 (d, *J* = 7.7 Hz, 1H), 4.57 (d, *J* =

12.0 Hz, 1H), 4.37 (d, J = 12.0 Hz, 1H), 4.23 (dq, J = 10.9, 7.1 Hz, 1H), 4.21 (dq, J = 10.9, 7.1 Hz, 1H), 3.95 (dd, J = 9.0, 2.5 Hz, 1H), 3.69 (ddd, J = 10.6, 10.6, 3.4 Hz, 1H), 3.58–3.47 (m, 1H), 3.01 (d, J = 18.2 Hz, 1H), 2.83 (ddd, J = 15.1, 10.6, 5.1 Hz, 1H), 2.55–2.47 (m, 3H), 2.20 (d, J = 18.2 Hz, 1H), 1.62–1.54 (m, 2H), 1.43–1.35 (m, 1H), 1.29 (t, J = 7.1 Hz, 3H), 1.30–1.20 (m, 3H), 1.19–1.10 (m, 1H), 0.86 (t, J = 7.1 Hz, 3H) ppm; **^{13}C NMR** (125 MHz, $CDCl_3$) δ 198.2, 165.8, 151.2, 137.8, 128.3 (2C), 127.6, 127.4 (2C), 122.6, 80.7, 71.4, 65.7, 60.9, 58.9, 55.2, 45.2, 43.1, 36.14, 36.10, 25.0, 23.1, 14.1, 13.9 ppm; **HRMS** calcd for $C_{24}H_{33}NO_5Na$ [M+Na$^+$]: 438.2251; found: 438.2249.

Synthesis of Compound 26

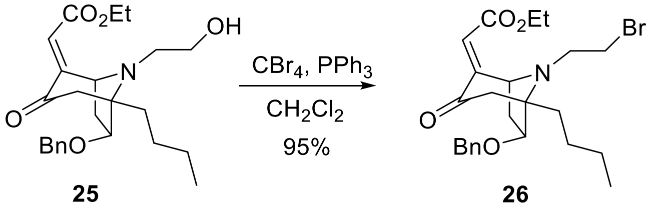

25 **26**

To a cooled solution (0 ºC) of alcohol **25** (1910 mg, 4.60 mmol) in anhydrous CH_2Cl_2 (50 mL) were added CBr_4 (1980 mg, 5.98 mmol) and PPh_3 (2500 mg, 6.90 mmol) under a nitrogen atmosphere. The resulting mixture was stirred at 0 ºC for 30 min and then concentrated under reduced pressure. The residue was purified by flash column chromatography on silica gel (EtOAc/PE = 1/10) to give bromide **26** (2080 mg, yield: 95%) as a colorless oil.

Compound **26**: $[\alpha]_D^{20}$ +87.9 (c 1.0, $CHCl_3$); **IR** (film) ν_{max}: 3029, 2956, 2931, 2868, 1701, 1453, 1368, 1236, 1181, 1130, 1028, 883, 737, 698 cm^{-1}; **^{1}H NMR** (500 MHz, $CDCl_3$) δ 7.35–7.22 (m, 5H), 6.58 (s, 1H), 5.22 (d, J = 7.8 Hz, 1H), 4.56 (d, J = 12.1 Hz, 1H), 4.36 (d, J = 12.1 Hz, 1H), 4.23 (dq, J = 11.0, 7.1 Hz, 1H), 4.21 (dq, J = 11.0, 7.1 Hz, 1H), 3.93 (dd, J = 9.0, 2.8 Hz, 1H), 3.44 (ddd, J = 9.6, 9.6, 4.6 Hz, 1H), 3.36 (ddd, J = 9.6, 7.9, 7.9 Hz, 1H), 3.00 (d, J = 18.1 Hz, 1H), 2.94 (ddd, J = 13.1, 7.9, 4.6 Hz, 1H), 2.78 (ddd, J = 13.1, 9.6, 7.9 Hz, 1H), 2.52 (ddd, J = 13.8, 9.0, 7.8 Hz, 1H), 2.18 (d, J = 18.1 Hz, 1H), 1.59–1.54 (m, 1H), 1.51 (dd, J = 13.8, 2.8 1H), 1.43–1.35 (m, 1H), 1.31 (t, J = 7.1 Hz, 3H), 1.29–1.22 (m, 3H), 1.19–1.09 (m, 1H), 0.87 (t, J = 7.1 Hz, 3H) ppm; **^{13}C NMR** (125 MHz, $CDCl_3$) δ 198.4, 165.9, 151.5, 137.9, 128.3

(2C), 127.6, 127.4 (2C), 122.3, 80.4, 71.4, 65.9, 60.9, 55.7, 45.9, 43.4, 36.0, 35.9, 30.4, 25.0, 23.2, 14.1, 13.9 ppm; **HRMS** calcd for $C_{24}H_{32}BrNO_4Na$ [M+Na$^+$]: 500.1407 and 502.1386; found: 500.1406 and 502.1384.

Synthesis of Compound 14

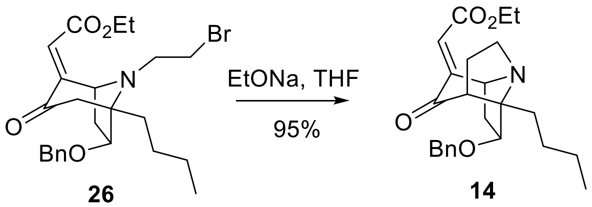

26 **14**

To a cooled solution (0 ℃) of bromide **26** (2080 mg, 4.36 mmol) in anhydrous THF (100 mL) was added under a nitrogen atmosphere a freshly prepared NaOEt (10.5 mL, 5.23 mmol, 0.5 M in THF and EtOH). The resulting mixture was stirred at the same temperature for 20 min, then quenched with a saturated aqueous solution of NH_4Cl (40 mL). The organic layer was separated and the aqueous layer was extracted with EtOAc (3 × 100 mL). The combined organic layers were washed with brine, dried over anhydrous Na_2SO_4, filtered, and concentrated under reduced pressure. The residue was purified by flash chromatography on silica gel (EtOAc/PE = 2/1) to give compound **14** (1640 mg, yield: 95%) as a colorless oil.

Compound **14**: $[\alpha]_D^{20}$ +27.3 (*c* 1.0, $CHCl_3$); **IR** (film) ν_{max}: 3029, 2957, 2933, 1707, 1454, 1236, 1180, 1098, 1030, 975, 735, 697 cm^{-1}; **^{1}H NMR** (500 MHz, $CDCl_3$) δ 7.35–7.21 (m, 5H), 6.63 (s, 1H), 5.01 (d, *J* = 6.1 Hz, 1H), 4.54 (d, *J* = 12.3 Hz, 1H), 4.37 (d, *J* = 12.3 Hz, 1H), 4.22 (dq, *J* = 10.9, 7.1 Hz, 1H), 4.19 (dq, *J* = 10.9, 7.1 Hz, 1H), 4.08 (d, *J* = 7.3 Hz, 1H), 3.06 (dd, *J* = 13.5, 3.6 Hz, 1H), 3.04 (d, *J* = 6.7 Hz, 1H), 2.95 (ddd, *J* = 13.5, 8.7, 6.7 Hz, 1H), 2.37 (ddd, *J* = 13.3, 6.7, 6.7 Hz, 1H), 2.32–2.23 (m, 1H), 1.83 (d, *J* = 13.4 Hz, 1H), 1.77 (ddd, *J* = 13.3, 8.7, 3.6 Hz, 1H), 1.57–1.50 (m, 1H), 1.40–1.20 (m, 5H), 1.30 (t, *J* = 7.1 Hz, 3H), 0.86 (t, *J* = 6.8 Hz, 3H) ppm; **^{13}C NMR** (125 MHz, $CDCl_3$) δ 198.8, 165.8, 153.3, 137.7, 128.3 (2C), 127.5, 127.3 (2C), 122.4, 82.0, 78.9, 71.6, 63.6, 60.7, 55.7, 46.1, 38.9, 33.9, 30.9, 26.1, 23.1, 14.1, 13.9 ppm; **HRMS** calcd for $C_{24}H_{31}NO_4Na$ [M+Na$^+$]: 420.2145; found: 420.2139.

Synthesis of Compound 13

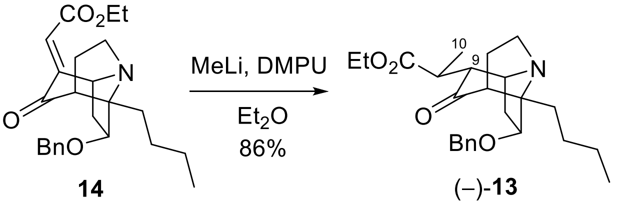

To a cooled solution (–40 ºC) of compound **14** (1640 mg, 4.13 mmol) in anhydrous ether (50 mL) under nitrogen atmosphere was added 1,3-dimethyl-tetrahydropyrimidin-2(1*H*)-one (DMPU) (0.55 mL, 4.54 mmol). After being stirred for 10 min, methyllithium (MeLi) (3.25 mL, 4.54 mmol, 1.4 M in ether) was added dropwise. The reaction mixture was stirred at the same temperature for 20 min, then quenched with a saturated aqueous solution of NH_4Cl (40 mL). The organic layer was separated and the aqueous layer was extracted with EtOAc (3 × 50 mL). The combined organic layers were washed with brine, dried over anhydrous Na_2SO_4, filtered, and concentrated under reduced pressure. The residue was purified by flash chromatography on silica gel (EtOAc/hex. = 2/1) to give compound **13** (1460 mg, yield: 86%) as a colorless oil (single diastereomer as seen for NMR spectra).

Compound **13**: $[\alpha]_D^{20}$ –24.9 (*c* 1.0, $CHCl_3$); **IR** (film) ν_{max}: 3036, 2935, 1716, 1673, 1455, 1378, 1262, 1183, 1102, 1072, 1029, 973, 736, 698 cm^{-1}; **^{1}H NMR** (500 MHz, $CDCl_3$) δ 7.35–7.24 (m, 5H), 4.52 (d, *J* = 12.2 Hz, 1H), 4.38 (d, *J* = 12.2 Hz, 1H), 4.24 (dq, *J* = 10.8, 7.1 Hz, 1H), 4.16 (dq, *J* = 10.8, 7.1 Hz, 1H), 4.00 (d, *J* = 8.3 Hz, 1H), 3.48 (t, *J* = 5.9 Hz, 1H), 3.18 (ddd, *J* = 13.7, 8.6, 5.5 Hz, 1H), 3.11 (ddd, *J* = 13.7, 9.9, 4.3 Hz, 1H), 2.92 (dd, *J* = 10.5, 5.6 Hz, 1H), 2.76 (d, *J* = 6.5 Hz, 1H), 2.54 (ddd, *J* = 14.3, 10.5, 7.3 Hz, 1H), 2.20–2.10 (m, 1H), 2.07–2.00 (m, 1H), 1.75 (ddd, *J* = 13.9, 8.6, 4.3 Hz, 1H), 1.60 (d, *J* = 13.9 Hz, 1H), 1.52–1.45 (m, 1H), 1.31 (t, *J* = 7.1 Hz, 3H), 1.28–1.15 (m, 5H), 1.05 (d, *J* = 7.1 Hz, 3H), 0.85 (t, *J* = 7.1 Hz, 3H) ppm; **^{13}C NMR** (125 MHz, $CDCl_3$) δ 207.3, 176.5, 138.0, 128.2 (2C), 127.42 (2C), 127.40, 81.5, 79.0, 71.7, 62.4, 60.5, 54.8, 48.4, 45.7, 37.6, 33.7, 32.5, 31.0, 25.9, 23.1, 14.4, 14.1, 13.9 ppm; **HRMS** calcd for $C_{25}H_{35}NO_4Na$ $[M+Na^+]$: 436.2458; found: 436.2457.

Synthesis of Compound 27

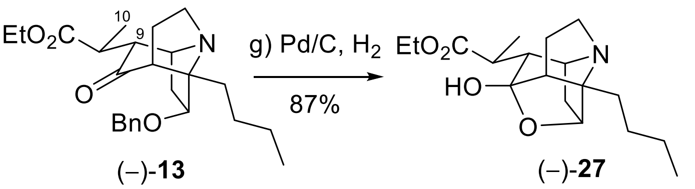

(–)-**13** (–)-**27**

A suspension of compound **13** (1460 mg, 3.54 mmol) and 10% Pd/C (730 mg) in ethanol (50 mL) was stirred under an atmosphere of H_2 for 2 days at room temperature. The mixture was filtered through a Celite pad by washing with methanol. The solvent was removed under reduced pressure. The residue was purified by flash chromatography on silica gel (EtOAc/PE = 4/1) to afford compound **27** (995 mg, yield: 87%) as a colorless oil.

Compound **27**: $[\alpha]_D^{20}$ –0.8 (*c* 1.0, $CHCl_3$); **IR** (film) ν_{max}: 3373, 2957, 2931, 1733, 1463, 1377, 1319, 1290, 1262, 1175, 1140, 1086, 1032, 979, 948, 912, 819, 757, 656 cm^{-1}; **^{1}H NMR** (500 MHz, $CDCl_3$) δ 4.29 (br s, 1H), 4.14 (q, J = 7.1 Hz, 2H), 3.27 (br s, 1H), 3.10-2.97 (m, 2H), 2.82-2.74 (m, 1H), 2.20 (dd, J = 9.8, 3.6 Hz, 1H), 2.14 (d, J = 6.0 Hz, 1H), 1.91-1.85 (m, 2H), 1.82 (d, J = 12.0 Hz, 1H), 1.68-1.60 (m, 1H), 1.55-1.48 (m, 2H), 1.42-1.30 (m, 3H), 1.28-1.21 (m, 2H), 1.25 (t, J = 7.1 Hz, 3H), 1.13 (d, J = 7.2 Hz, 3H), 0.90 (t, J = 7.2 Hz, 3H) ppm; **^{13}C NMR** (125 MHz, $CDCl_3$) δ 178.0, 105.5, 82.0, 79.9, 62.5, 60.6, 55.8, 47.3, 41.2, 36.9, 33.6, 31.5, 27.3, 26.2, 23.2, 15.0, 14.04, 13.99 ppm; **HRMS** calcd for $C_{18}H_{29}NO_4$ $[M+Na^+]$: 346.1989; found: 346.1986.

Synthesis of Compound 12 (two-step manner from Compound 13)

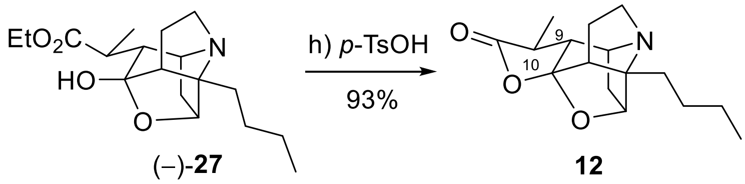

(–)-**27** **12**

A solution of compound **27** (995 mg, 3.08 mmol) and *p*-toluenesulfonic acid (PTSA) monohydrate (879 mg, 4.62 mmol) in toluene (30 mL) was stirred at 85 °C for 2 h. After being cooled down to room temperature, a saturated aqueous $NaHCO_3$ was added till pH = 8. The organic layer was separated, and the aqueous layer was extracted with EtOAc (3 × 30 mL). The combined organic layers were washed with brine, dried over anhydrous Na_2SO_4, filtered, and concentrated under reduced pressure. The residue was

purified by flash chromatography on silica gel (CH_2Cl_2/MeOH= 20/1) to give compound **12** (775 mg, yield: 91%) as a colorless oil.

^{1}H NMR and ^{13}C NMR data of **12** matched by-product by Kende's total synthesis of isostemofoline[2] and semi-synthesis compound by Pyne and Ung[3].

Compound **12**: $[\alpha]_D^{20}$ +24.4 (*c* 1.0, $CHCl_3$) {lit[3] $[\alpha]_D^{20}$ +26.3 (*c* 0.21, $CHCl_3$) (semi-synthetic compound by Pyne and Ung)}; **IR** (film) ν_{max}: 2955, 2933, 2869, 1799, 1458, 1235, 1133, 1081, 1021, 980, 831, 751 cm^{-1};**^{1}H NMR** (500 MHz, $CDCl_3$) δ 4.33 (br s, 1H), 3.41 (br s, 1H), 3.15 (ddd, *J* = 13.6, 10.4, 5.4 Hz, 1H), 3.02 (ddd, *J* = 13.6, 8.5, 4.6 Hz, 1H), 2.81–2.73 (m, 1H), 2.65 (d, *J* =6.2 Hz, 1H), 1.98 (d, *J* = 12.7 Hz, 1H), 1.96 (d, *J* = 12.7 Hz, 1H), 1.95–1.89 (m, 1H), 1.82 (ddd, *J* = 13.9, 8.5, 5.4 Hz, 1H), 1.78–1.72 (m, 1H), 1.63–1.50 (m, 2H), 1.45–1.39 (m, 1H), 1.38–1.30 (m, 2H), 1.25 (d, *J* = 7.0 Hz, 3H), 1.29–1.21 (m, 1H), 0.92 (t, *J* = 7.2 Hz, 3H) ppm; **^{13}C NMR** (125 MHz, $CDCl_3$) δ 178.3, 109.1, 83.0, 78.8, 61.1, 50.1, 47.6, 45.6, 35.7, 32.8, 31.8, 27.2, 26.5, 23.1, 14.0, 13.2 ppm; **HRMS** calcd for $C_{16}H_{24}NO_4$ [M+H$^+$]: 278.1751; found: 278.1747.

Synthesis of Compound 12 from Compound 13 without further purification

A suspension of compound **13** (1145 mg, 2.77 mmol) and 10% Pd/C (580 mg) in ethanol (50 mL) was stirred under an atmosphere of H_2 for 2 days at room temperature. The mixture was filtered through a Celite pad, and washed with ethanol. The solvent was removed under reduced pressure. The residue C was directly used in the next synthetic step without further purification.

A solution of the residue C and *p*-toluenesulfonic acid (PTSA) monohydrate (791 mg, 4.16 mmol) in toluene (30 mL) was stirred at 85 °C for 2 h. After being cooled down to room temperature, the resulting mixture was quenched with saturated aqueous solution of $NaHCO_3$ till pH reached 8. The organic layer was separated and the aqueous layer was extracted with EtOAc (3 × 30 mL). The combined organic layers were washed with brine, dried over anhydrous Na_2SO_4, filtered, and concentrated under reduced pressure. The residue was purified by flash chromatography (CH_2Cl_2/MeOH= 20/1) to give compound **12** (637 mg, yield: 83%, from compound **13**) as a colorless oil.

Synthesis of Compound 11

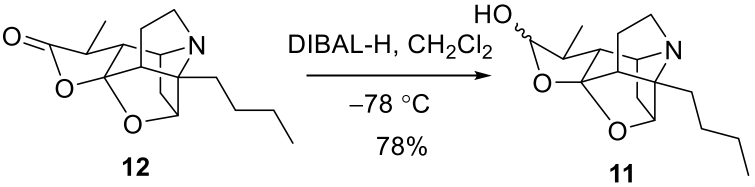

To a cooled solution (–78 ºC) of compound **12** (775 mg, 2.80 mmol) in anhydrous CH_2Cl_2 (50 mL) was added diisobutyl aluminium hydride (DIBAL-H) (3.10 mL, 3.10 mmol, 1.0 M in toluene) dropwise under a nitrogen atmosphere. After being stirred at the same temperature for 30 min, the reation was quenched with a saturated aqueous solution of NH_4Cl (40 mL). The organic layer was separated and the aqueous layer was extracted with CH_2Cl_2 (3 × 50 mL). The combined organic layers were washed with brine, dried over anhydrous Na_2SO_4, filtered, and concentrated under reduced pressure. The residue was purified by flash chromatography on silica gel (CH_2Cl_2/MeOH= 10/1) to give lactol **11** (609 mg, yield: 78%) as an inseparable diastereoisomeric mixture (*d.r.* = 2.6: 1), which was used in the next step as it was.

Compound **11**: **IR** (film) ν_{max}: 3357, 2957, 2931, 2873, 1720, 1456, 1338, 1119, 1031, 931, 754, 687 cm^{-1}; **^{1}H NMR** (500 MHz, $CDCl_3$, data of the diastereomeric mixture) δ 5.47 (d, J = 5.5 Hz, 0.3H), 5.01 (br s, 0.7H), 4.26 (s, 0.7H), 4.18 (br s, 0.3H), 4.18 (br s, 0.3H), 3.38 (br s, 0.3H), 3.33 (br s, 0.7H), 3.15–3.06 (m, 1H), 3.04–2.93 (m, 1H), 2.50–2.36 (m, 1.3H), 2.22–2.14 (m, 0.7H), 1.99 (t, J = 11.7 Hz, 0.7H), 1.91 (t, J = 12.0 Hz, 0.3H), 1.89–1.70 (m, 2H), 1.69–1.60 (m, 2H), 1.60–1.48 (m, 2H), 1.44–1.30 (m, 3H), 1.30–1.20 (m, 1H), 1.14 (t, J = 6.4 Hz, 2H), 1.03 (t, J = 6.9 Hz, 1H), 0.91 (t, J = 7.1 Hz, 3H) ppm; **^{13}C NMR** (125 MHz, $CDCl_3$, data of the diastereomeric mixture) δ 112.3, 112.0, 108.4, 101.1, 82.4, 82.1, 78.8, 78.1, 61.0, 50.4, 50.2, 47.6, 47.5, 47.3, 43.0, 41.0, 38.9, 33.4, 33.0, 31.7, 27.4, 27.3, 26.6, 26.3, 23.2, 14.8, 14.0, 11.1 ppm; **HRMS** calcd for $C_{16}H_{26}NO_3$ [M+H$^+$]: 280.1907; found: 280.1915.

Synthesis of Compound 10

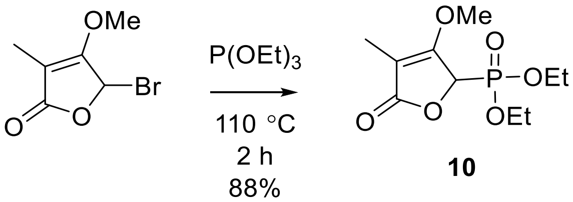

The mixture of the known compound 5-bromo-4-methoxy-3-methylfuran-2(5*H*)-one[4] (2050 mg, 10.0 mmol) and $P(OEt)_3$ (2.2 mL, 13.0 mmol) was stirred at 110 ºC for 2 h. The mixture was concentrated under reduced pressure, and the residue was purified by flash column chromatography on silica gel (EtOAc/PE = 4/1) to give compound **10** (2320 mg, yield: 88%) as a pale yellow oil.

Compound **10**: **IR** (film) ν_{max}: 2986, 2914, 1763, 1662, 1455, 1396, 1337, 1263, 1023, 977 cm^{-1}; **^{1}H NMR** (400 MHz, $CDCl_3$) δ 4.91 (d, $J_{P\text{-}H}$ = 10.7 Hz, 1H), 4.29–4.16 (m, 4H), 4.15 (s, 3H), 1.97 (d, J = 2.8 Hz, 3H), 1.36 (t, J = 7.1 Hz, 3H), 1.31 (t, J = 7.1 Hz, 3H) ppm; **^{13}C NMR** (100 MHz, $CDCl_3$) δ 173.9, 169.4 (d, $J_{P\text{-}C}$ = 5.2 Hz, 1C), 99.7 (d, $J_{P\text{-}C}$ = 5.2 Hz, 1C), 73.3 (d, $J_{P\text{-}C}$ = 162.2 Hz, 1C), 64.3 (d, $J_{P\text{-}C}$ = 6.4 Hz, 1C), 63.9 (d, $J_{P\text{-}C}$ = 6.4 Hz, 1C), 59.4, 16.4 (d, $J_{P\text{-}C}$ = 5.6 Hz, 2C), 8.3 ppm; **HRMS** calcd for $C_{10}H_{17}O_6PNa$ [M+Na$^+$]: 287.0660; found: 287.0655.

Completion of the total synthesis of (+)-Stemoburkilline (7), (+)-(11*S*,12*R*)-Dihydrostemofoline (8), and (+)-(11*S*,12*S*)-Dihydrostemofoline (9)

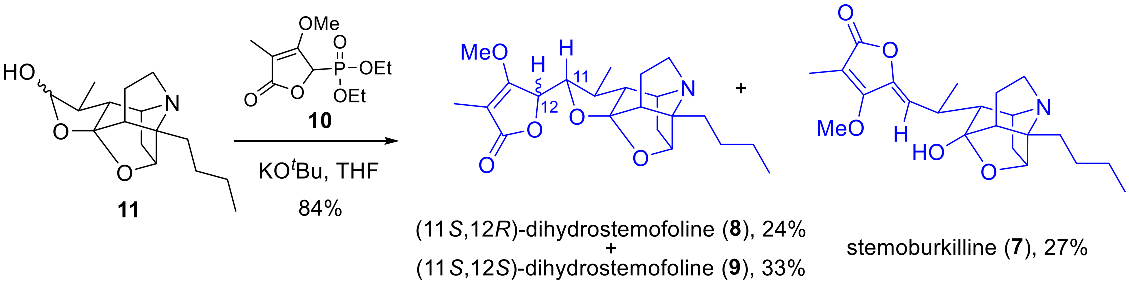

To a cooled suspension (0 ºC) of compound **10** (1440 mg, 5.45 mmol) in anhydrous THF (20 mL) was added KO^{t}Bu (665 mg, 5.45 mmol) under a nitrogen atmosphere. The suspension was stirred for 30 min. To the resulting mixture was added dropwise a solution of compound **11** (305 mg, 1.09 mmol) in THF (10 mL). After being stirred at the same temperature for 1 h, the reaction was quenched with a saturated aqueous solution of NH_4Cl (10 mL). The organic layer was separated and the aqueous layer was

extracted with EtOAc (3 × 30 mL). The combined organic layers were washed with brine, dried over anhydrous Na_2SO_4, filtered, and concentrated under reduced pressure. The residue was purified by flash chromatography on silica gel (EtOAc/MeOH = 5/1) to give (+)-stemoburkilline (**7**) (114 mg, colorless oil, yield: 27%), (+)-(11*S*,12*R*)-dihydrostemofoline (**8**) (101 mg, colorless oil, yield: 24%), and (+)-(11*S*,12*S*)-dihydrostemofoline (**9**) (140 mg, colorless oil, yield: 33%), combined yield: 84%. The mixture of the three products (**7**, **8**, and **9**) was used without separation in the total synthesis of stemofoline (**1**).

(+)-Stemoburkilline (**7**): $[\alpha]_D^{20}$ +5.1 (*c* 1.0, $CHCl_3$) {lit.[6] $[\alpha]_D^{24}$ +7.6 (*c* 0.64, $CHCl_3$) (semi-synthetic compound by Pyne); lit.[5] $[\alpha]_D^{26}$ +37.5 (*c* 0.28, $CHCl_3$) (natural product)}; **IR** (film) ν_{max}: 3331, 2958, 2929, 2873, 1755, 1633, 1454, 1355, 1032, 987, 756, 666 cm^{-1}; **^{1}H NMR** (500 MHz, $CDCl_3$) δ 5.47 (d, *J* = 10.0 Hz, 1H), 4.32 (br s, 1H), 4.11 (s, 3H), 3.34 (br s, 1H), 3.20–3.12 (m, 1H), 3.12–3.05 (m, 1H), 3.01–2.94 (m, 1H), 2.19 (d, *J* = 5.5 Hz, 1H), 2.05 (s, 3H), 1.96–1.91 (m, 1H), 1.90–1.82 (m, 2H), 1.76 (dd, *J* = 9.2, 3.5 Hz, 1H), 1.67–1.61 (m, 1H), 1.55–1.50 (m, 2H), 1.44–1.37 (m, 1H), 1.36–1.30 (m, 2H), 1.28–1.21 (m, 1H), 1.05 (d, *J* = 6.9 Hz, 3H), 0.90 (t, *J* = 7.2 Hz, 3H) ppm; **^{13}C NMR** (125 MHz, $CDCl_3$) δ 170.5, 161.8, 142.0, 114.7, 105.9, 99.3, 82.2, 80.3, 63.6, 58.9, 55.6, 47.3, 44.7, 33.4, 31.3, 28.3, 27.3, 26.2, 23.1, 18.5, 14.0, 8.6 ppm; **HRMS** calcd for $C_{22}H_{32}NO_5$ [M+H$^+$]: 390.2275; found: 390.2271.

(+)-(11*S*,12*R*)-Dihydrostemofoline (**8**): $[\alpha]_D^{20}$ +28.3 (*c* 1.0, $CHCl_3$) {lit.[5] $[\alpha]_D^{26}$ +38.9 (*c* 0.35, $CHCl_3$) (natural product)}; **IR** (film) ν_{max}: 2957, 2930, 2873, 1756, 1671, 1458, 1353, 1031, 961, 756 cm^{-1}; **^{1}H NMR** (500 MHz, $CDCl_3$) δ 4.59 (br s, 1H), 4.23 (br s, 1H), 4.11(s, 3H), 3.79 (dd, *J* = 9.0, 3.0 Hz, 1H), 3.48 (br s, 1H), 3.18–3.13 (m, 1H), 3.03–2.99 (m, 1H), 2.64–2.59 (m, 1H), 2.47 (d, *J* = 6.0 Hz, 1H), 2.01 (br s, 3H), 2.02–1.98 (m, 1H), 1.89–1.84 (m, 1H), 1.74–1.70 (m, 1H), 1.64 (dd, *J* = 12.0, 3.0 Hz, 1H), 1.67–1.62 (m, 1H), 1.56 (t, *J* = 8.0 Hz, 2H), 1.41–1.37 (m, 1H), 1.35–1.29 (m, 2H), 1.25–1.20 (m, 1H), 1.08 (d, *J* = 6.5 Hz, 3H), 0.89 (t, *J* = 7.0 Hz, 3H) ppm; **^{13}C NMR** (125 MHz, $CDCl_3$) δ 174.6, 170.3, 111.8, 98.6, 86.3, 82.6, 78.1, 76.5, 61.2, 58.9, 50.5, 47.4, 47.3, 33.4, 33.1, 31.3, 27.2, 26.4, 23.1, 14.8, 13.9, 8.7 ppm; **HRMS** calcd for $C_{22}H_{31}NO_5Na$ [M+Na$^+$]: 412.2094; found: 412.2091.

(+)-(11*S*,12*S*)-Dihydrostemofoline (**9**): $[\alpha]_D^{20}$ +18.7 (*c* 1.0, $CHCl_3$) {lit.[5] $[\alpha]_D^{26}$ +35.9 (*c* 0.22, $CHCl_3$) (semi-synthetic compound by Pyne)}; **IR** (film) ν_{max}: 2957, 2876, 1758, 1666, 1461, 1339, 1030, 957, 757, 666 cm^{-1}; **^{1}H NMR** (500 MHz, $CDCl_3$) δ 4.75 (d, *J* = 6.5 Hz, 1H), 4.21 (br s, 1H), 4.10 (s, 3H), 3.69 (t, *J* = 7.0 Hz, 1H), 3.40 (br s ,1H), 3.16–3.07 (m, 1H), 3.02–2.94 (m, 1H), 2.55–2.45 (m, 1H), 2.47 (d, *J* = 6.0 Hz, 1H), 1.98–1.94 (m, 1H), 1.95 (br s ,3H), 1.89–1.83 (m, 1H), 1.76–1.70 (m, 1H), 1.65–1.59 (m, 2H), 1.56–1.51 (m, 2H), 1.44–1.37 (m, 1H), 1.34 (q, *J* = 6.8 Hz, 2H), 1.26–1.21 (m, 1H), 1.12 (d, *J* = 6.3 Hz, 3H), 0.91 (t, *J* = 6.8 Hz, 3H) ppm; **^{13}C NMR** (125 MHz, $CDCl_3$) δ 174.3, 173.1, 112.8, 98.8, 87.8, 82.3, 78.7, 78.6, 61.1, 59.3, 50.5, 47.5, 47.3, 35.0, 33.4, 31.7, 27.3, 26.7, 23.3, 16.5, 14.0, 8.3 ppm; **HRMS** calcd for $C_{22}H_{31}NO_5Na$ [M+Na$^+$]: 412.2094; found: 412.2085.

Synthesis of (+)-Stemoburkilline (7)

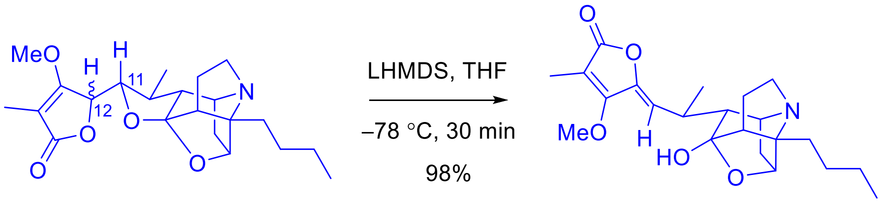

(+)-(11*S*,12*R*)-dihydrostemofoline (**8**)
+
(+)-(11*S*,12*S*)-dihydrostemofoline (**9**)

(+)-stemoburkilline (**7**)

To a cooled solution (–78 ºC) of a mixture of diastereomers (+)-(11*S*,12*R*)-dihydrostemofoline **8** and (+)-(11*S*,12*S*)-dihydrostemofoline **9** (89.5 mg, 0.23 mmol) in anhydrous THF (5 mL) was added lithium bis(trimethylsilyl)amide (LiHMDS) (0.28 mL, 0.28 mmol, 1.0 M in THF) under a nitrogen atmosphere. After being stirred at the same temperature for 30 min, the reaction was quenched with a saturated aqueous solution of NH_4Cl (5 mL). The organic layer was separated and the aqueous layer was extracted with EtOAc (3 × 30 mL). The combined organic layers were washed with brine, dried over anhydrous Na_2SO_4, filtered, and concentrated under reduced pressure. The residue was purified by flash chromatography on silica gel (CH_2Cl_2/MeOH = 20/1) to give (+)-stemoburkilline **7** (87.7 mg, yield: 98%) as a colorless oil.

Asymmetric Total Synthesis of (+)-Stemofoline (1), (+)-Isostemofoline (2):

Supplementary Table 1. Attempted conversion of 7 into 1 by Wacker-type reaction

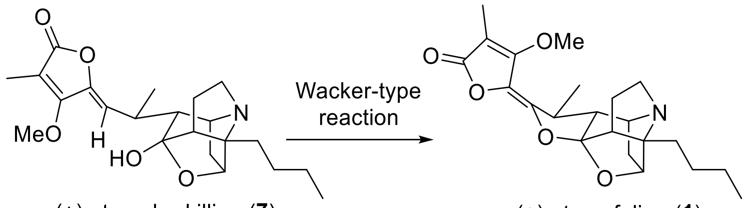

entry	Conditions	yield
1	Na_2PdCl_4, TBHP, NaOAc, AcOH, *t*-BuOH/H_2O, rt	ND[a]
2	Na_2PdCl_4, TBHP, NaOAc, AcOH, dioxane/H_2O, rt	ND
3	$PdCl_2$, CuCl, O_2, Na_2HPO_4, DME, 85°C	NR[b]
4[c]	$PdCl_2$, CuCl, O_2, Na_2HPO_4, DME, 85°C	NR
5[c]	$Pd(TFA)_2$[d], MeCN, rt to 65 °C	NR
6[c]	$Pd(TFA)_2$, IMes, DMAP, Na_2CO_3, toluene, rt	decomp.

[a]ND = Not detected. [b]NR = No reaction. [c]Hydrochloride salt of stemoburkilline (**7**) used as the starting material. [d]2.0 equiv of Pd used.

Supplementary Table 2. Attempted transformations of 8/9 to 28

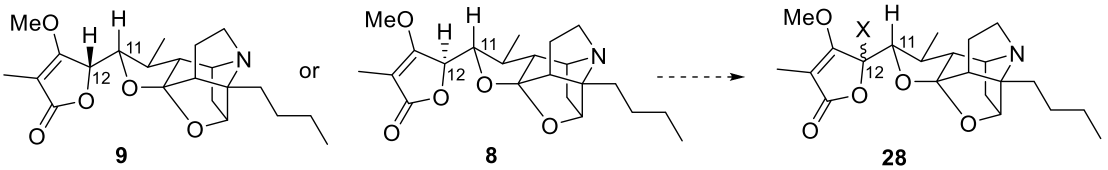

Entry	Conditions	Products/Yield
1	IBX, DMSO	NR[a]
2	O_2, DBU, MeCN, reflux	Mixture of **7**, **8**, **9**
3	LDA, NIS, THF, –78 °C to rt	Mixture of **7**, **8**, **9**
4	DBU, NIS, THF, rt to 60 °C	Mixture of **7**, **8**, **9**
5	LDA, NBS, THF, –78 °C to rt	Only **7**
6	DBU, NBS, THF, rt to 60 °C	Mixture of **7**, **8**, **9**

7	LDA, MeSSMe, –78 °C to rt	Only **7**

[a]NR = No reaction.

Supplementary Table 3. Attempted transformations of 8/9 to 29

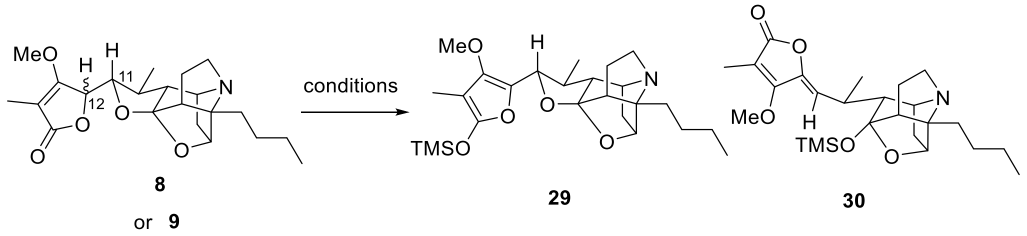

Entry	Conditions	Yield
1	Et_3N, TMSOTf, CH_2Cl_2, –20 °C to rt	58% for SM and 30%% for **30**
2	LHMDS, TMSCl, THF, –78 °C	93% for **30**
3	HMDS, TMSI, CH_2Cl_2, 0 °C to rt	27% for SM and 55% for **30**
4	DBU, TMSOTf, CH_2Cl_2, –20 °C to rt	11% for SM and 71% for **30**
5	TMSI, CH_3CN, reflux	NR[a]

[a]NR = No reaction.

Supplementary Table 4. Attempted transformations of 7 to 33

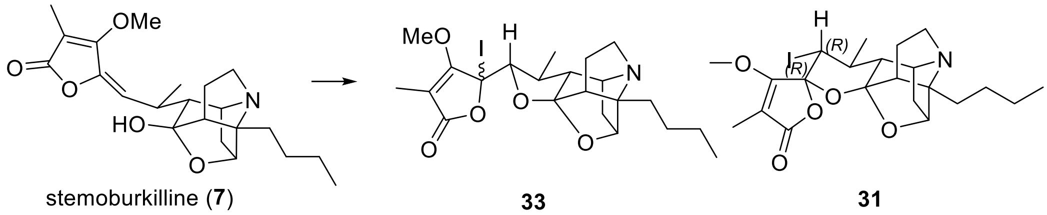

Entry	Conditions	Yield
1	NIS, CH_2Cl_2, rt to reflux	NR[a]
2	NIS, CH_3CN, rt to reflux	NR
3	NIS, THF, rt to reflux	NR
4	NIS, Et_3N, CH_2Cl_2, rt to reflux	Mixture of **7**, **8**, **9**
5	NIS, DBU, CH_2Cl_2, rt to 35 °C	Mixture of **7**, **8**, **9**
6	NIS, DBU, THF, rt to 60°C	Mixture of **7**, **8**, **9**
7	NIS, NaOEt, THF, 60°C	Mixture of **7**, **8**, **9**
8	NIS, NaH, THF, 60°C	Mixture of **7**, **8**, **9**
9	NIS, LDA, THF, –78 °C	Only **7**

10	NIS, ^{t}BuOK, THF, rt	decomposed
11	PhSeCl, K_2CO_3, THF, 60°C	ND[b]
12	I_2, $NaHCO_3$, THF, rt	86% for **31**

[a]NR = No reaction. [b]ND = Not detected.

Synthesis of Compound 30

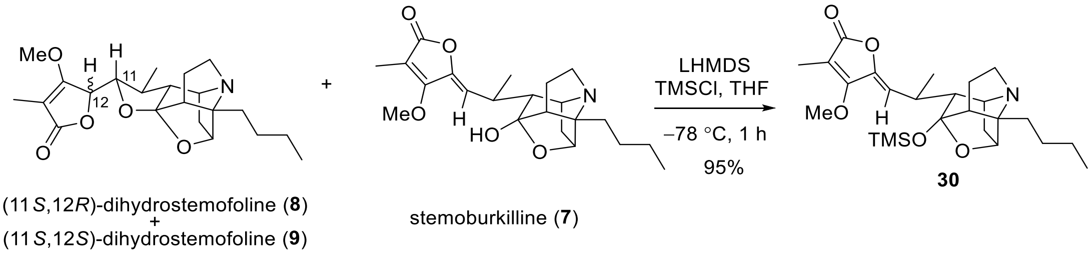

To a cooled solution (–78 ºC) of the mixture of the products obtained from the HWE reaction of **11**: **7**, **8** and **9** (59.2 mg, 0.15 mmol) in anhydrous THF (5 mL), was added lithium bis(trimethylsilyl)amide (LiHMDS) (0.23 mL, 0.23 mmol, 1.0 M in THF) under a nitrogen atmosphere. The mixture was stirred for 30 min before addition of trimethyl chlorosilane (TMSCl) (0.029 mg, 0.23 mmol). After being stirred at –78 ºC for 1 h, the reaction was quenched with a saturated aqueous solution of NH_4Cl (5 mL). The organic layer was separated and the aqueous layer was extracted with EtOAc (3 × 30 mL). The combined organic layers were washed with brine, dried over anhydrous Na_2SO_4, filtered, and concentrated under reduced pressure. The residue was purified by flash chromatography on silica gel (CH_2Cl_2/MeOH = 20/1) to give compound **30** (65.6 mg, yield: 95%) as a colorless oil.

Compound **30**: **M.p.**: 141~142 °C; $[\alpha]_D^{20}$ −10.5 (*c* 1.0, $CHCl_3$); **IR** (film) ν_{max}: 2956, 2875, 1760, 1635, 1455, 1356, 1248, 1037, 977, 844, 755 cm^{-1}; **^{1}H NMR** (500 MHz, $CDCl_3$) δ 5.33 (d, *J* =9.0 Hz, 1H), 4.27 (br s, 1H), 4.10 (s, 3H), 3.31 (br s, 1H), 3.09 (ddd, *J* =13.4, 8.0, 8.0 Hz, 1H), 3.02–2.98 (m, 1H), 2.96 (dd, *J* =13.4, 6.8, 6.8 Hz, 1H), 2.20 (dd, *J* =3.3, 3.3 Hz, 1H), 2.05 (s, 3H), 1.90 (d, *J* =11.9 Hz, 1H), 1.87–1.82 (m, 2H), 1.70 (dd, *J* =9.5, 3.3 Hz, 1H), 1.60 (ddd, *J* =12.0, 3.3, 3.3 Hz, 1H), 1.64–1.57 (m, 1H), 1.55–1.46 (m, 2H), 1.38–1.31 (m, 3H), 1.24–1.19 (m, 1H), 1.00 (d, *J* = 7.1 Hz, 3H), 0.90 (t, *J* = 7.0 Hz, 3H), 0.01 (s, 9H) ppm; **^{13}C NMR** (125 MHz, $CDCl_3$) δ 171.1, 162.4, 140.0, 118.4, 107.2, 98.2, 81.9, 79.7, 63.3, 58.7, 55.6, 47.3, 45.4, 33.3, 31.2, 28.4,

27.3, 26.1, 23.1, 18.1, 13.9, 8.6, 1.5 (3C) ppm; **HRMS** calcd for $C_{25}H_{40}NO_5Si$ [M+H$^+$]: 462.2670; found: 462.2677.

Synthesis of Compound 31

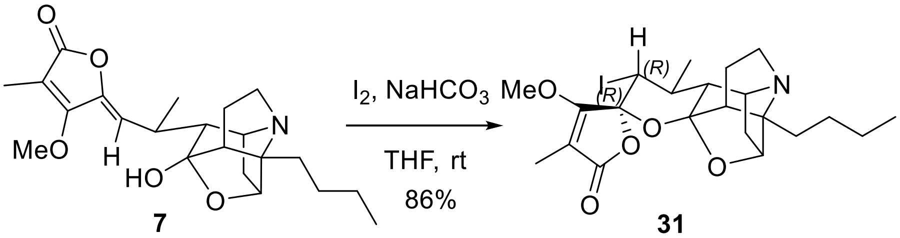

A solution of (+)-stemoburkilline **7** (24.0 mg, 0.062 mmol) in THF (2 mL) was added $NaHCO_3$ (26.0 mg, 0.31 mmol), and the suspension was stirred for 5 min. To the resulting mixture was added I_2 (78.0 mg, 0.31 mmol), and the reaction was stirred overnight before being quenched with a saturated aqueous NaS_2O_3 (3 mL). The organic layer was separated and the aqueous layer was extracted with EtOAc (3 × 20 mL). The combined organic layers were washed with brine, dried over anhydrous Na_2SO_4, filtered, and concentrated under reduced pressure. The residue was purified by flash chromatography on silica gel (MeOH/CH_2Cl_2 = 1/20) to give compound **31** (27.1 mg, yield: 86%) as a pale yellow oil.

Compound **31**: $[\alpha]_D^{20}$ +33.5 (*c* 1.0, $CHCl_3$); **IR** (film) ν_{max}: 2959, 2926, 2854, 1748, 1634, 1260, 1074, 1015, 799, 755, 665 cm^{-1}; **^{1}H NMR** (500 MHz, $CDCl_3$) δ 4.46 (br s, 1H), 4.15 (d, *J* =13.0 Hz, 1H), 4.13 (br s, 3H), 3.44 (s, 1H), 3.15 (ddd, *J* =13.5, 10.6, 5.4 Hz, 1H), 2.99 (ddd, *J* =13.5, 8.6, 5.1 Hz, 1H), 2.67–2.54 (m, 1H), 2.36 (d, *J* =6.2 Hz, 1H), 2.02 (s, 3H), 2.01 (d, *J* =9.2 Hz, 1H), 1.93–1.87 (m, 1H), 1.84 (ddd, *J* =13.9, 10.6, 5.1 Hz, 1H), 1.67 (dd, *J* =13.0, 3.1 Hz, 1H), 1.59–1.50 (m, 3H), 1.45–1.36 (m, 1H), 1.32 (dd, *J* =14.3, 7.2 Hz, 2H), 1.24–1.17 (m, 1H), 1.14 (d, *J* = 6.6 Hz, 3H), 0.88 (t, *J* = 7.2 Hz, 3H) ppm; **^{13}C NMR** (125 MHz, $CDCl_3$) δ 171.6, 168.2, 105.5, 101.5, 100.3, 81.2, 81.1, 63.1, 59.4, 54.4, 47.2, 43.6, 37.3, 33.0, 32.2, 31.4, 27.2, 26.6, 23.1, 20.6, 13.9, 8.0 ppm; **HRMS** calcd for $C_{22}H_{30}INO_5$ [M+Na$^+$]: 538.1061; found: 538.1062.

Synthesis of (+)-Stemofoline (1), (+)-Isostemofoline (2), and (+)-Stemoburkilline (7)

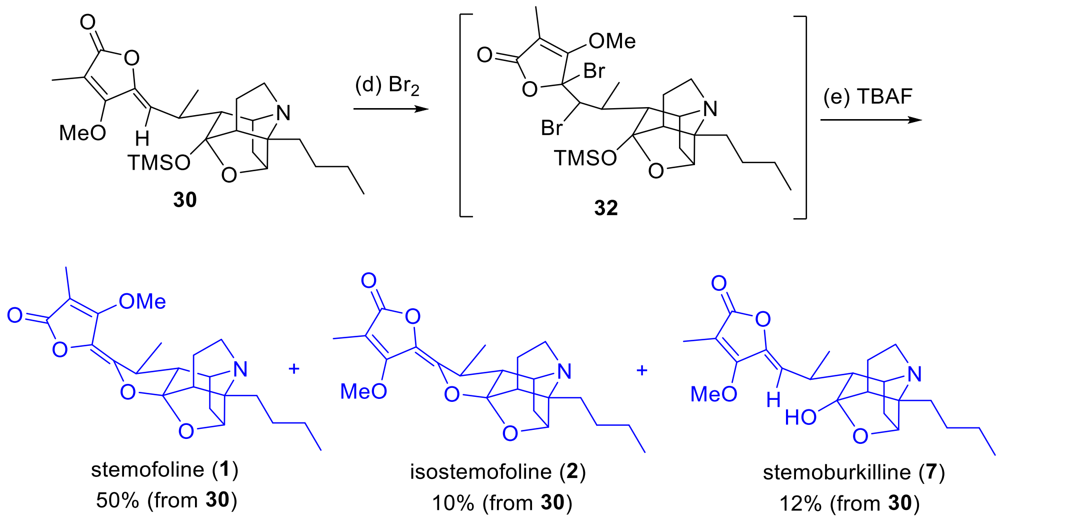

To a solution of compound **30** (40.0 mg, 0.087 mmol) in CH_2Cl_2 (10 mL) was added dropwise Br_2 (0.022 mL, 0.43 mmol).After being stirred for 30 min, the reaction was quenched with a saturated aqueous solution of NaS_2O_3 (3 mL). The organic layer was separated and the aqueous layer was extracted with EtOAc (3 × 30 mL). The combined organic layers were washed with brine, dried over anhydrous Na_2SO_4, filtered, and concentrated under reduced pressure. The presumed dibromination product **32** was directly used in the next step without further purification.

To a solution of the crude **32** in THF (5 mL) at 0 °C was added dropwise tetrabutylammonium fluoride (TBAF) (0.45 mL, 0.45 mmol, 1.0 M in THF) under a nitrogen atmosphere. The mixture was stirred for 1 h before being quenched with a saturated aqueous solution of NH_4Cl (3 mL). The organic layer was separated and the aqueous layer was extracted with EtOAc (3 × 30 mL). The combined organic layers were washed with brine, dried over anhydrous Na_2SO_4, filtered, and concentrated under reduced pressure. The residue was purified by flash chromatography on silica gel (CH_2Cl_2/MeOH = 20/1) to give (+)-stemofoline (**1**) (16.6 mg, colorless oil, yield: 50%), (+)-isostemofoline (**2**) (3.4 mg, colorless oil, yield: 10%) and (+)-stemoburkilline (**7**) (3.9 mg, colorless oil, yield: 12%).

(+)-Stemofoline (**1**): $[\alpha]_D^{20}$ +276 (*c* 1.0, MeOH) {lit.[7] $[\alpha]_D$ +273 (MeOH); lit.[8] $[\alpha]_D^{20}$ +270 (*c* 0.8, MeOH)}; **IR** (film) ν_{max}: 2956, 2928, 2855, 1746, 1621, 1365, 1141, 1006, 754, 677 cm^{-1}; **^{1}H NMR** (850 MHz, $CDCl_3$) δ 4.25 (br s, 1H), 4.13 (s, 3H), 3.46 (br s,

1H), 3.13 (ddd, J = 13.6, 10.0, 5.1 Hz, 1H), 3.09 (dd, J = 10.0, 6.6 Hz, 1H), 2.99 (ddd, J = 13.6, 8.8, 4.7 Hz, 1H), 2.69 (d, J = 6.3 Hz, 1H), 2.07 (s, 3H), 1.95 (d, J = 12.1 Hz, 1H), 1.93–1.88 (m, 1H), 1.84–1.82 (m, 1H), 1.81 (dd, J = 10.0, 3.6 Hz, 1H), 1.72–1.69 (m, 1H), 1.58–1.52 (m, 2H), 1.44–1.39 (m, 1H), 1.37 (d, J = 6.5 Hz, 3H), 1.36–1.32 (m, 2H), 1.28–1.22 (m, 1H), 0.91 (t, J = 7.1 Hz, 3H) ppm; **^{13}C NMR** (216 MHz, $CDCl_3$) δ 169.7, 162.8, 148.4, 127.9, 112.7, 98.5, 82.8, 78.5, 60.9, 58.8, 49.9, 47.6, 47.5, 34.5, 33.3, 31.6, 27.3, 26.7, 23.1, 18.3, 14.0, 9.2 ppm; **HRMS** calcd for $C_{22}H_{30}NO_5$ [M+H$^+$]: 388.2118; found: 388.2126.

(+)-Isostemofoline (**2**): colorless oil. $[\alpha]_D^{20}$ +102.1 (c 0.5, $CHCl_3$) (the optical rotation of this alkaloid has not been reported previously); **IR** (film) ν_{max}: 2957, 2929, 2857, 1747, 1620, 1446, 1396, 1157, 1025, 752, 674 cm^{-1}; **^{1}H NMR** (850 MHz, $CDCl_3$) δ 4.29 (br s, 1H), 4.13 (s, 3H), 3.50–3.48 (m, 1H), 3.24–3.19 (m, 1H), 3.15 (ddd, J = 13.9, 10.4, 5.0 Hz, 1H), 3.02 (ddd, J = 13.9, 8.8, 4.6 Hz, 1H), 2.71 (d, J = 6.2 Hz, 1H), 2.06 (s ,3H), 1.99 (d, J = 12.2 Hz, 1H), 1.96–1.91 (m, 1H), 1.84 (ddd, J = 13.9, 8.8, 5.3 Hz, 1H), 1.75 (dd, J = 10.8, 3.6 Hz, 1H), 1.72 (dt, J = 12.3, 3.3 Hz, 1H), 1.63–1.55 (m, 2H), 1.46–1.41 (m, 1H), 1.47 (d, J = 6.5 Hz, 3H), 1.37 (q, J = 7.0 Hz, 2H), 1.30–1.26 (m, 1H), 0.94 (t, J = 7.0 Hz, 3H) ppm; **^{13}C NMR** (216 MHz, $CDCl_3$) δ 169.7, 162.8, 148.4, 127.9, 112.7, 98.5, 82.8, 78.5, 60.9, 58.8, 49.9, 47.6, 47.5, 34.5, 33.3, 31.6, 27.3, 26.7, 23.1, 18.3, 14.0, 9.2 ppm; **HRMS** calcd for $C_{22}H_{30}NO_5$ [M+H$^+$]: 388.2118; found: 388.2122.

^{1}H NMR and ^{13}C NMR data of (+)-stemoburkilline (**7**) matched those described in page S21.

Supplementary Note 4

Comparison of ^{1}H and ^{13}C NMR Data of Our Synthetic Compounds (1, 2, 7-9) with Those of Reported Data

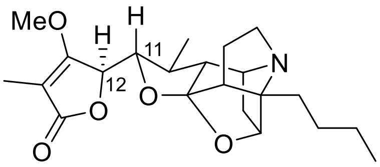

Supplementary Table 5. Comparison of ^{1}H NMR Spectral Data of (11*S*,12*R*)-dihydrostemofoline (**8**)

^{1}H NMR	
Our synthetic compound (500 MHz, $CDCl_3$)	Natural product [5] (500 MHz, $CDCl_3$)
4.59 (br s, 1H)	4.60 (br s, 1H)
4.23 (br s, 1H)	4.22 (br s, 1H)
4.11(s, 3H)	4.11 (s, 3H)
3.79 (dd, *J* = 9.0, 3.0 Hz, 1H)	3.79 (dd, *J* = 9, 3 Hz, 1H)
3.48 (br s, 1H)	3.44 (br s, 1H)
3.18–3.13 (m, 1H)	3.14 (m, 1H)
3.03–2.99 (m, 1H)	3.01 (m, 1H)
2.64–2.59 (m, 1H)	2.61 (m, 1H)
2.47 (d, *J* = 6.0 Hz, 1H)	2.45 (d, *J* = 6 Hz, 1H)
2.01 (br s, 3H)	2.01 (br s, 3H)
2.02–1.98 (m, 1H)	1.99 (m, 1H)
1.89–1.84 (m, 1H)	1.82 (m, 1H)
1.74–1.70 (m, 1H)	1.72 (m, 1H)
1.64 (dd, *J* = 12.0, 3.0 Hz, 1H)	1.64 (dd, *J* = 12, 3 Hz, 1H)
1.67–1.62 (m, 1H)	1.63 (dd, *J* = 7.5, 3 Hz, 1H)
1.56 (t, *J* = 8.0 Hz, 2H)	1.56 (t, *J* = 8 Hz, 2H)
1.41–1.37 (m, 1H)	1.40 (m, 1H)
1.35–1.29 (m, 2H)	1.33 (m, 2H)
1.25–1.20 (m, 1H)	1.23 (m, 1H)
1.08 (d, *J* = 6.5 Hz, 3H)	1.08 (d, *J* = 6.5 Hz, 3H)
0.89 (t, *J* = 7.0 Hz, 3H)	0.87 (t, *J* = 7 Hz, 3H)

Supplementary Table 6. Comparison of ^{13}C NMR Spectral Data of (11*S*,12*R*)-dihydrostemofoline (**8**)

^{13}C NMR	
Our synthetic compound (125 MHz, $CDCl_3$)	Natural product[5] (125 MHz, $CDCl_3$)
174.6	174.5
170.3	170.3
111.8	111.8
98.6	98.5
86.3	86.3
82.6	82.2
78.1	78.2
76.5	76.5
61.2	61.0
58.9	58.8
50.5	50.5
47.4	47.4
47.3	47.3
33.4	33.4
33.1	33.1
31.3	31.5
27.2	27.2
26.4	26.5
23.1	23.2
14.8	14.8
13.9	13.9
8.7	8.7

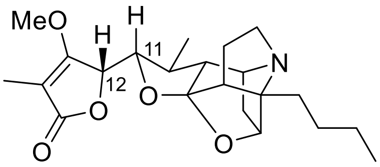

Supplementary Table 7. Comparison of ^{1}H NMR Spectral Data of (11*S*,12*S*)-dihydrostemofoline (**9**)

^{1}H NMR	
Our synthetic compound (500 MHz, $CDCl_3$)	Semi-synthetic compound [5] (500 MHz, $CDCl_3$)
4.75 (d, *J* = 6.5 Hz, 1H)	4.75 (d, *J* = 6.5 Hz, 1H)
4.21 (br s, 1H)	4.21 (br s, 1H)
4.10 (s, 3H)	4.10 (s, 3H)
3.69 (t, *J* = 7.0 Hz, 1H)	3.69 (t, *J* = 7 Hz, 1H)
3.40 (br s, 1H)	3.38 (br s, 1H)
3.16–3.07 (m, 1H)	3.09 (m, 1H)
3.02–2.94 (m, 1H)	2.96 (m, 1H)
2.55–2.45 (m, 1H)	2.48 (m, 1H)
2.47 (d, *J* = 6.0 Hz, 1H)	2.47 (d, *J* = 6 Hz, 1H)
1.98–1.94 (m, 1H)	1.97 (m, 1H)
1.95 (br s, 3H)	1.95 (br s, 3H)
1.89–1.83 (m, 1H)	1.85 (m, 1H)
1.76–1.70 (m, 1H)	1.73 (m, 1H)
1.65–1.59 (m, 2H)	1.62 (m, 1H)
	1.60 (m, 1H)
1.56–1.51 (m, 2H)	1.54 (m, 2H)
1.44–1.37 (m, 1H)	1.40 (m, 1H)
1.34 (q, *J* = 6.8 Hz, 2H)	1.35 (q, *J* = 6.8 Hz, 2H)
1.26–1.21 (m, 1H)	1.23 (m, 1H)
1.12 (d, *J* = 6.3 Hz, 3H)	1.12 (d, *J* = 6.3 Hz, 3H)
0.91 (t, *J* = 6.8 Hz, 3H)	0.92 (t, *J* = 6.8 Hz, 3H)

Supplementary Table 8. Comparison of ^{13}C NMR Spectral Data of (11*S*,12*S*)-dihydrostemofoline (**9**)

^{13}C NMR	
Our synthetic compound (125 MHz, $CDCl_3$)	Semi-synthetic compound[5] (125 MHz, $CDCl_3$)
174.3	174.3
173.1	173.3
112.8	112.8
98.8	98.8
87.8	87.8
82.3	82.1
78.7	78.7
78.6	78.6
61.1	61.1
59.3	59.4
50.5	50.5
47.5	47.5
47.3	47.2
35.0	35.1
33.4	33.4
31.7	31.8
27.3	27.4
26.7	26.8
23.3	23.4
16.5	16.9
14.0	14.1
8.3	8.2

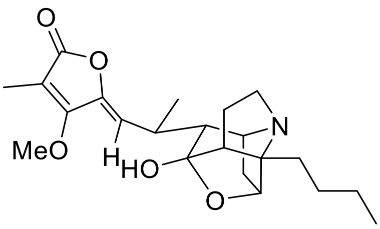

Supplementary Table 9. Comparison of ^{1}H NMR Spectral Data of Stemoburkilline (**7**)

^{1}H NMR		
Our synthetic compound (500 MHz, $CDCl_3$)	Natural product[5] (500 MHz, $CDCl_3$)	Semi-synthetic compound[6] (500 MHz, $CDCl_3$)
5.47 (d, J = 10.0 Hz, 1H)	5.50 (d, J = 10 Hz, 1H)	5.48 (d, J = 10 Hz, 1H)
4.32 (br s, 1H)	4.38 (br s, 1H)	4.30 (br s, 1H)
4.11 (s, 3H)	4.13 (s, 3H)	4.10 (s, 3H)
3.34 (br s, 1H)	3.60 (br s, 1H)	3.28 (br s, 1H)
3.20–3.12 (m, 1H)	3.27 (m, 1H)	3.13 (m, 1H)
3.12–3.05 (m, 1H)	3.18 (m, 1H)	3.05 (m, 1H)
3.01–2.94 (m, 1H)	3.09 (m, 1H)	2.94 (m, 1H)
2.19 (d, J = 5.5 Hz, 1H)	2.34 (d, J = 5.9 Hz, 1H)	2.15 (d, J = 5.0 Hz, 1H)
2.05 (s, 3H)	2.07 (s, 3H)	2.05 (s, 3H)
1.96–1.91 (m, 1H)	1.99 (m, 1H)	1.91 (m, 1H)
1.90–1.82 (m, 2H)	1.93 (m, 2H)	1.83 (m, 2H)
1.76 (dd, J = 9.2, 3.5 Hz, 1H)	1.85 (m, 1H)	1.74 (m, 1H)
1.67–1.61 (m, 1H)	1.80 (m, 1H)	1.59 (m, 1H)
1.55–1.50 (m, 2H)	1.67 (m, 1H)	1.48 (m, 2H)
	1.60 (m, 1H)	
1.44–1.37 (m, 1H)	1.39 (m, 1H)	1.38 (m, 1H)
1.36–1.30 (m, 2H)	1.36 (m, 2H)	1.32 (m, 2H)
1.28–1.21 (m, 1H)	1.29 (m, 1H)	1.24 (m, 1H)
1.05 (d, J = 6.9 Hz, 3H)	1.08 (d, J = 6.8 Hz, 3H)	1.05 (d, J = 6.5 Hz, 3H)
0.90 (t, J = 7.2 Hz, 3H)	0.92 (t, J = 6.8 Hz, 3H)	0.90 (t, J = 7.5 Hz, 3H)

Supplementary Table 10. Comparison of ^{13}C NMR Spectral Data of Stemoburkilline (**7**)

^{13}C NMR		
Our synthetic compound (125 MHz, $CDCl_3$)	Natural product[5] (125 MHz, $CDCl_3$)	Semi-synthetic compound [6] (125 MHz, $CDCl_3$)
170.5	170.5	170.8
161.8	161.8	162.1
142.0	141.9	142.1
114.7	114.7	115.2
105.9	105.7	106.2
99.3	99.1	99.5
82.2	82.9	82.1
80.3	79.8	80.6
63.6	63.4	63.8
58.9	59.3	59.1
55.6	55.4	55.9
47.3	46.5	47.5
44.7	44.2	45.0
33.4	32.9	33.7
31.3	30.2	31.7
28.3	28.4	28.6
27.3	27.5	27.5
26.2	25.3	26.7
23.1	23.7	23.4
18.5	18.7	18.8
14.0	14.1	14.2
8.6	8.7	8.8

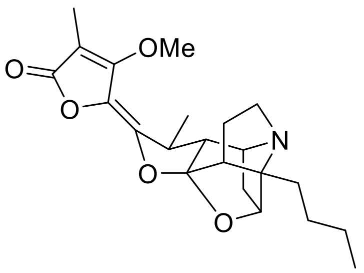

Supplementary Table 11. Comparison of ^{1}H NMR Spectral Data of stemofoline (**1**)

^{1}H NMR		
Our synthetic compound (850 MHz, $CDCl_3$)	Natural product[9] ($CDCl_3$)	Natural product[8] ($CDCl_3$)
4.25 (br s, 1H)	4.28 (br s, 1H)	4.25 (br s, 1H)
4.13 (s, 3H)	4.15 (s, 3H)	4.13 (s, 3H)
3.46 (br s, 1H)	3.53 (br s, 1H)	3.46 (br s, 1H)
3.13 (ddd, J = 13.6, 10.0, 5.1 Hz, 1H)	3.18 (ddd, 1H)	3.13 (ddd, J = 13.6, 9.8, 5.1 Hz, 1H)
3.09 (dd, J = 10.0, 6.6 Hz, 1H)	3.10 (dd, J = 9.9, 6.6 Hz, 1H)	3.08 (dd, J = 10.4, 6.6 Hz, 1H)
2.99 (ddd, J = 13.6, 8.8, 4.7 Hz, 1H)	3.04 (ddd, 1H)	2.99 (ddd, J = 13.6, 8.6, 5.1 Hz, 1H)
2.69 (d, J = 6.3 Hz, 1H)	2.72 (d, J = 6.0 Hz, 1H)	2.69 (d, J = 6.3 Hz, 1H)
2.07 (s, 3H)	2.07 (s, 3H)	2.07 (s, 3H)
1.95 (d, J = 12.1 Hz, 1H)	1.98 (d, J = 12.2 Hz, 1H)	1.95 (d, J = 12.1 Hz, 1H)
1.93–1.88 (m, 1H)	1.89 (m, 2H)	1.87 (m, 2H)
1.84–1.82 (m, 1H)		
1.81 (dd, J = 10.0, 3.6 Hz, 1H)	1.83 (dd, J = 10.0, 3.6 Hz, 1H)	1.81 (dd, J = 10.4, 2.7 Hz, 1H)
1.72–1.69 (m, 1H)	1.75 (m, 1H)	1.69 (m, 1H)
1.58–1.52 (m, 2H)	1.59 (m, 2H)	1.55 (m, 2H)
1.44–1.39 (m, 1H)	1.44 (m, 1H)	1.42 (m, 1H)
1.37 (d, J = 6.5 Hz, 3H)	1.38 (d, J = 6.5 Hz, 3H)	1.37 (d, 3H)
1.36–1.32 (m, 2H)	1.34 (m, 2H)	1.33(m, 2H)
1.28–1.22 (m, 1H)	1.25 (m, 1H)	1.24 (m, 1H)
0.91 (t, J = 7.1 Hz, 3H)	0.93 (t, J = 6.9 Hz, 3H)	0.91 (t, 3H)

Supplementary Table 12. Comparison of ^{13}C NMR Spectral Data of stemofoline (**1**)

^{13}C NMR		
Our synthetic compound (216 MHz, $CDCl_3$)	Natural product[9] ($CDCl_3$)	Natural product[8] ($CDCl_3$)
169.7	169.7	169.7
162.8	162.8	162.8
148.4	148.3	148.4
127.9	127.9	127.9
112.7	112.6	112.7
98.5	98.6	98.6
82.8	83.2	82.8
78.5	78.4	78.6
60.9	61.1	60.9
58.8	58.9	58.8
49.9	49.8	49.9
47.6	47.5	47.6
47.5	47.4	47.6
34.5	34.5	34.6
33.3	33.2	33.3
31.6	31.4	31.6
27.3	27.3	27.3
26.7	26.4	26.7
23.1	23.1	23.1
18.3	18.3	18.3
14.0	13.9	14.0
9.2	9.2	9.2

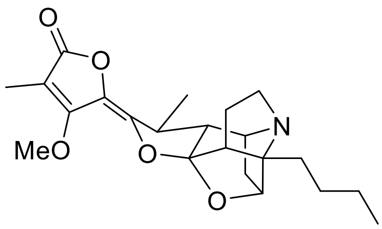

Supplementary Table 13. Comparison of ^{1}H NMR Spectral Data of isostemofoline (**2**)

^{1}H NMR (only ^{1}H NMR data is available)	
Our synthetic compound (850 MHz, $CDCl_3$)	Kende's synthetic compound[3] (400 MHz, $CDCl_3$)
4.29 (s, 1H)	4.29 (s, 1H)
4.13 (s, 3H)	4.12 (s, 3H)
3.50–3.48 (m, 1H)	3.49 (m, 1H)
3.24–3.19 (m, 1H)	3.20 (m, 2H)
3.15 (ddd, *J* = 13.9, 10.4, 5.0 Hz, 1H)	
3.02 (ddd, *J* = 13.9, 8.8, 4.6 Hz, 1H)	3.03 (m, 1H)
2.71 (d, *J* = 6.2 Hz, 1H)	2.73 (d, *J* = 5.2 Hz, 1H)
2.06 (s ,3H)	2.05 (s ,3H)
1.99 (d, *J* = 12.2 Hz, 1H)	2.00 (m, 2H)
1.96–1.91 (m, 1H)	
1.84 (ddd, *J* = 13.9, 8.8, 5.3 Hz, 1H)	1.84 (m, 1H)
1.75 (dd, *J* = 10.8, 3.6 Hz, 1H)	1.74 (dd, *J* = 10.8, 3.6 Hz, 2H)
1.72 (dt, *J* = 12.3, 3.3 Hz, 1H)	
1.63–1.55 (m, 2H)	1.58 (m, 3H)
1.46–1.41 (m, 1H)	
1.47 (d, *J* = 6.5 Hz, 3H)	1.46 (d, *J* = 6.4 Hz, 3H)
1.37 (q, *J* = 7.0 Hz, 2H)	1.36 (q, *J* = 6.8 Hz, 2H)
1.30–1.26 (m, 1H)	1.28 (m, 1H)
0.94 (t, *J* = 7.0 Hz, 3H)	0.92 (t, *J* = 6.8 Hz, 3H)

Supplementary Note 5

NOESY spectra of compounds 24, 13, 12 and 31

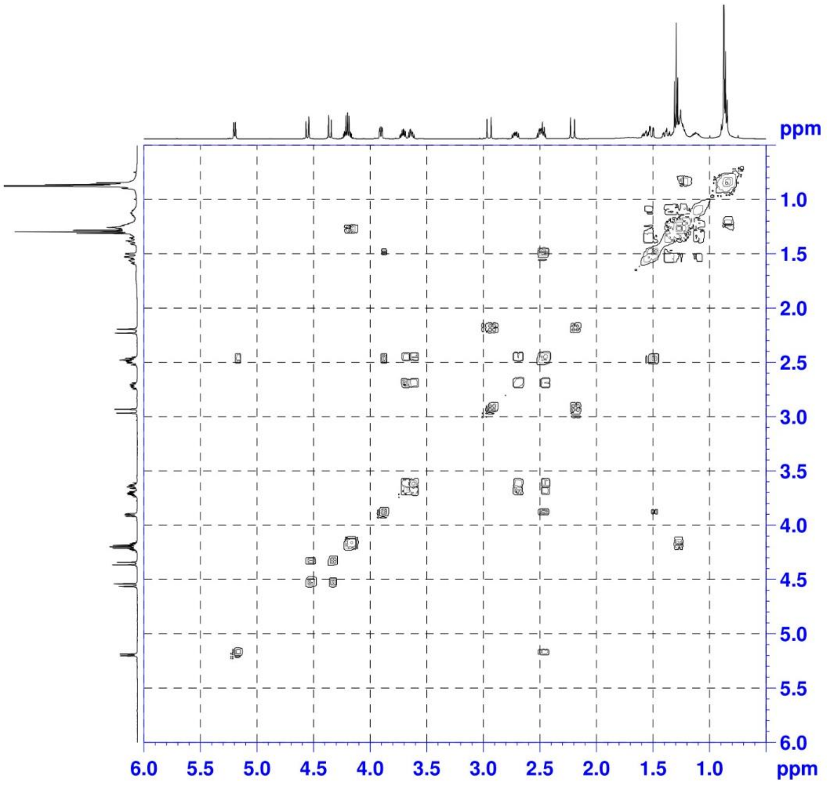

Supplementary Figure 2. ^{1}H-^{1}H COSY spectrum of compound **24**

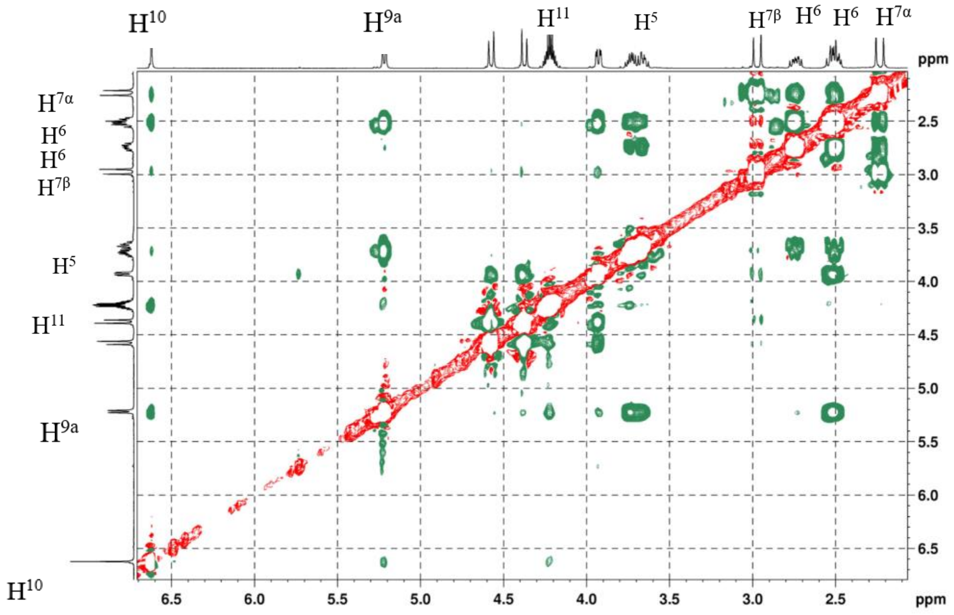

Supplementary Figure 3. NOESY spectrum of compound **24**

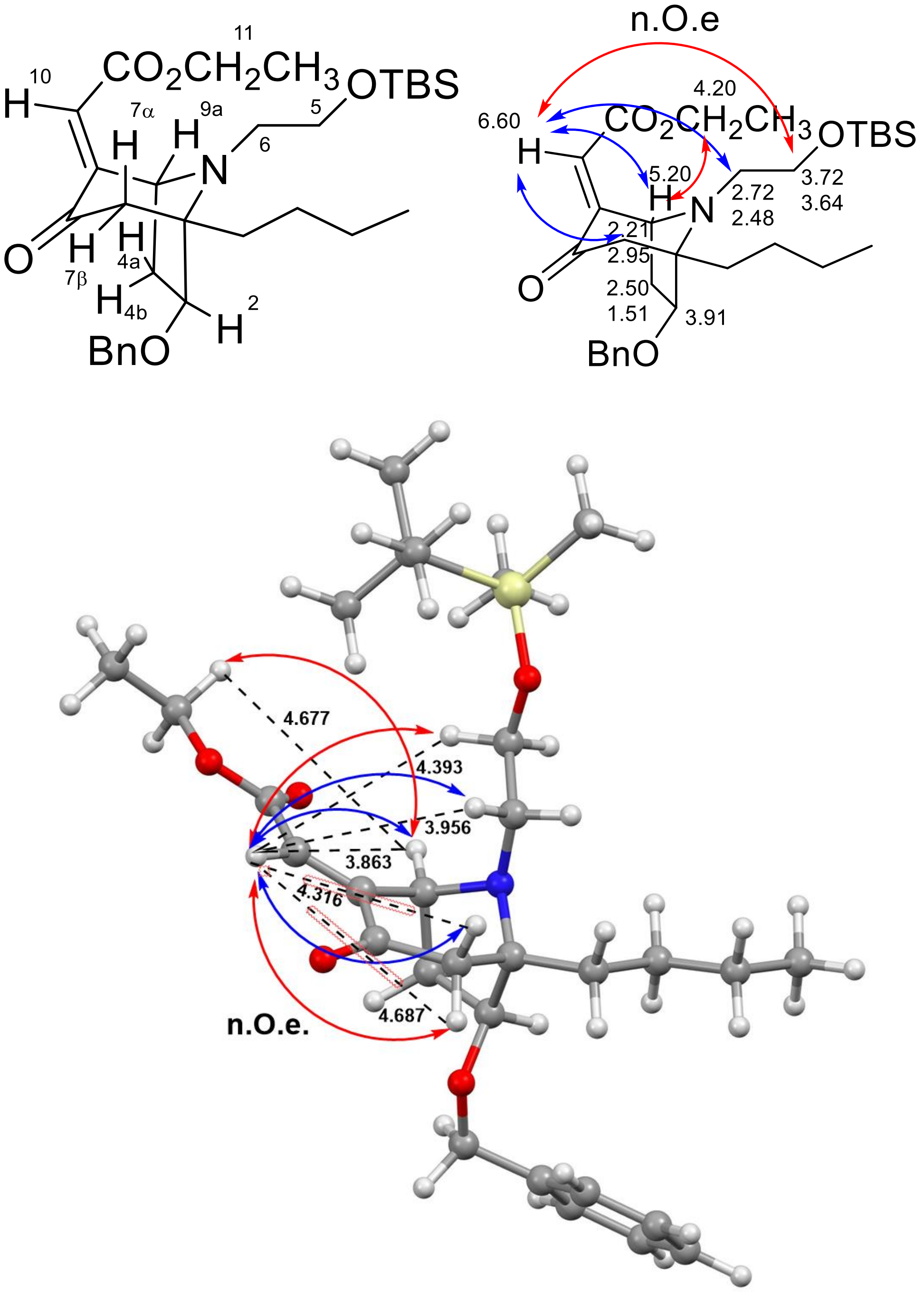

Supplementary Figure 4. Geometry of **24** optimized at b3lyp/6-31G* level and key correlations shown in NOESY spectrum

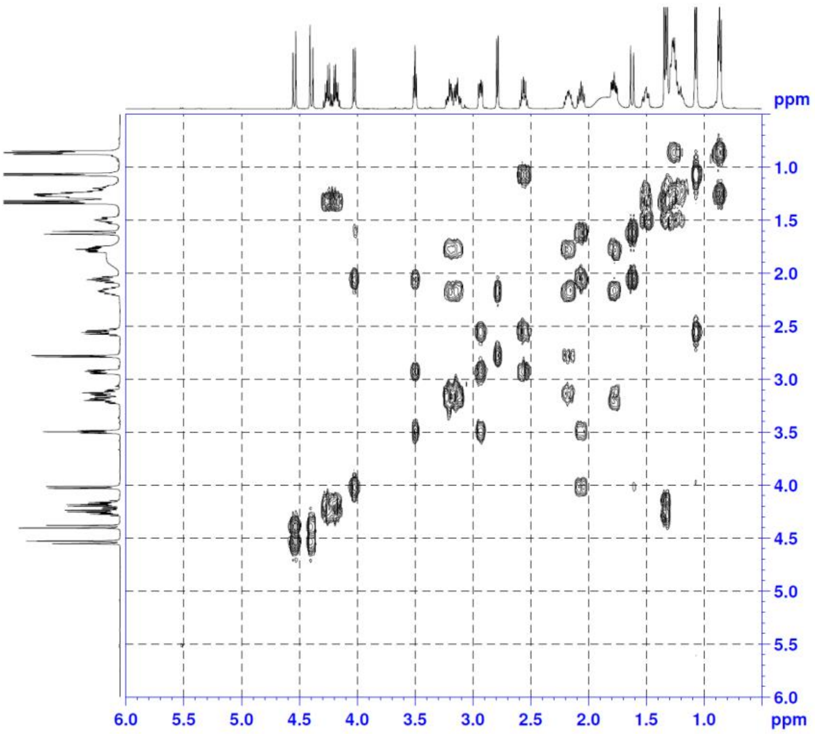

Supplementary Figure 5. ^{1}H-^{1}H COSY spectrum of compound **13**

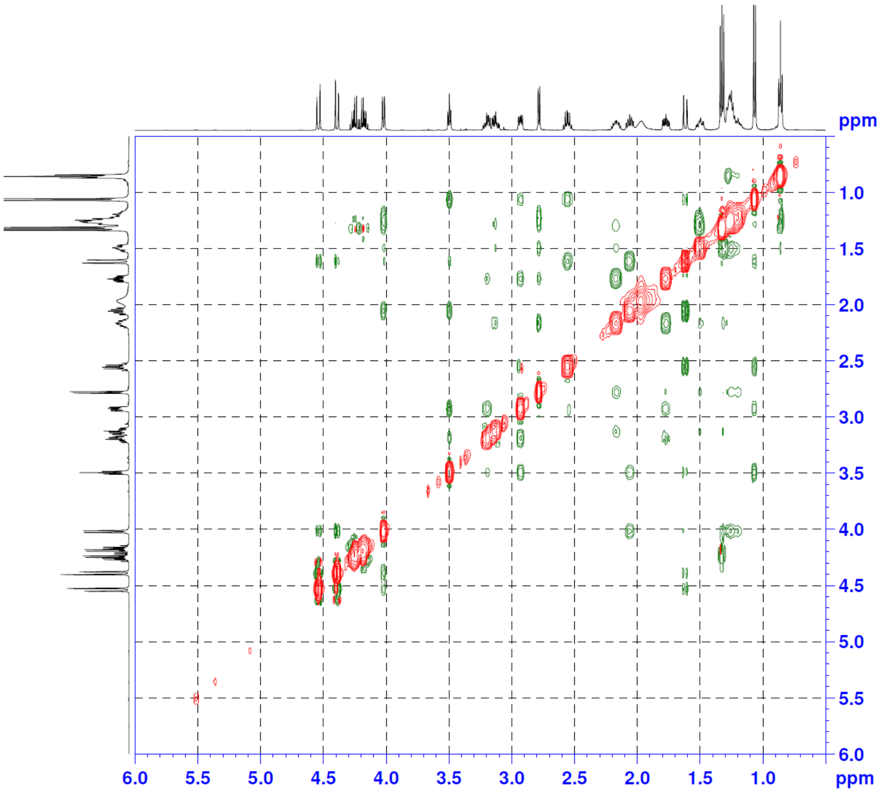

Supplementary Figure 6. NOESY spectrum of compound **13**

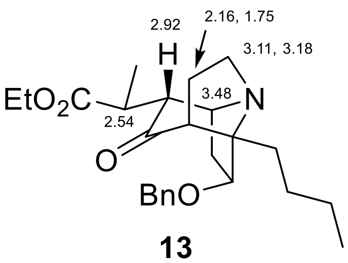

13

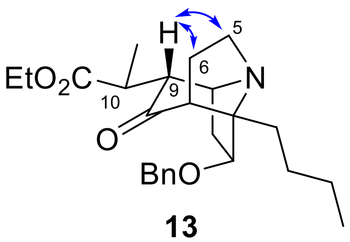

13

Supplementary Figure 7. Key correlations of compound **13** shown in NOESY spectrum

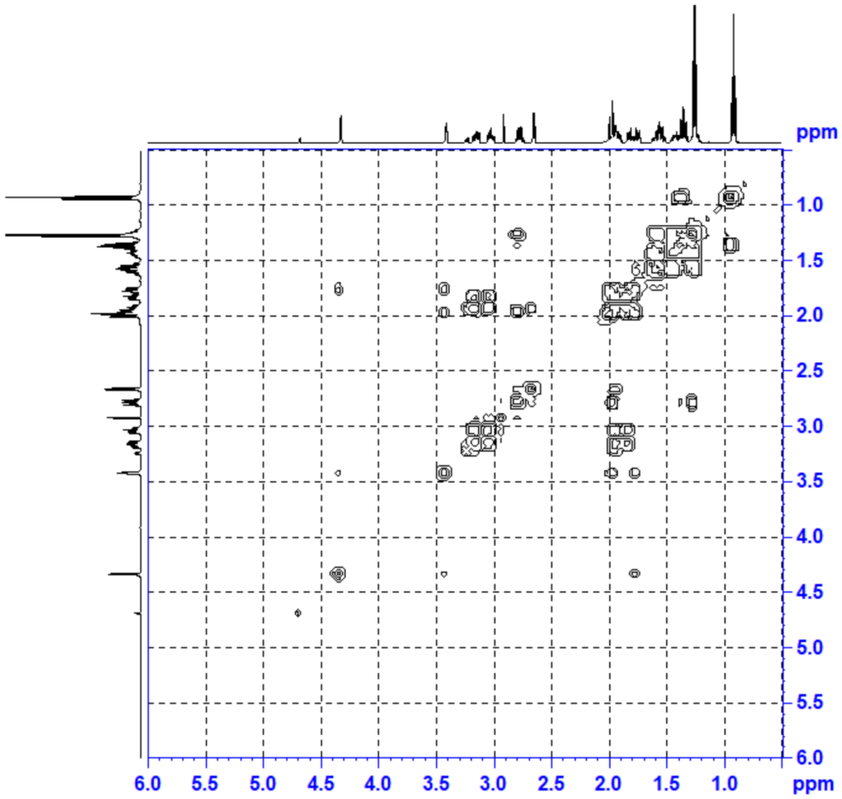

Supplementary Figure 8. ^{1}H-^{1}H COSY spectrum of compound **12**

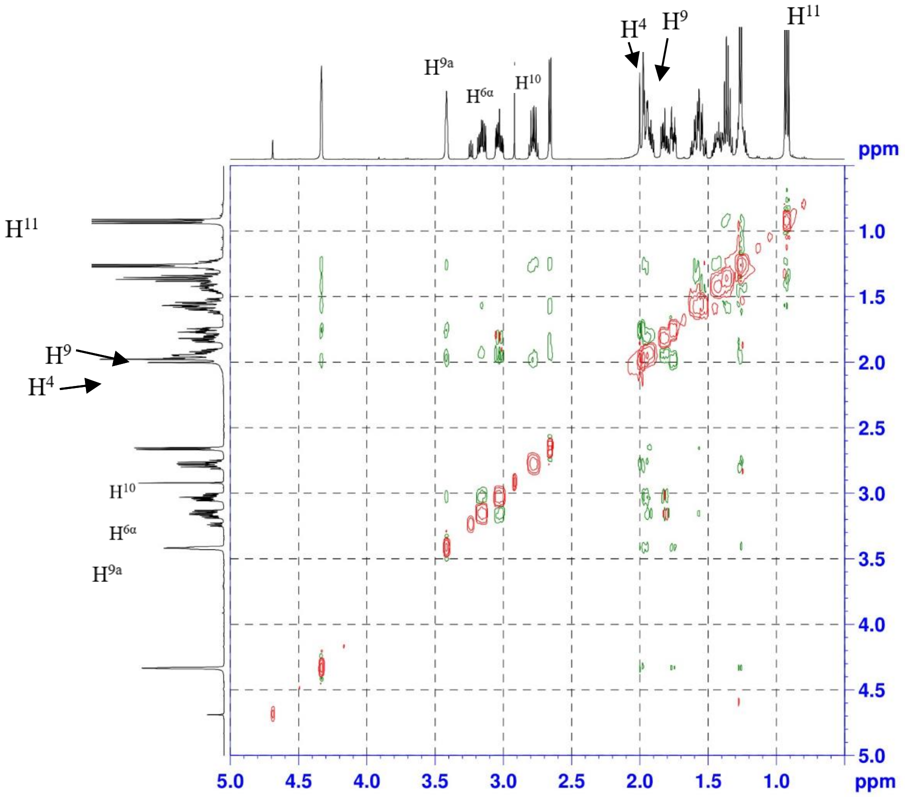

Supplementary Figure 9. NOESY spectrum of compound **12**

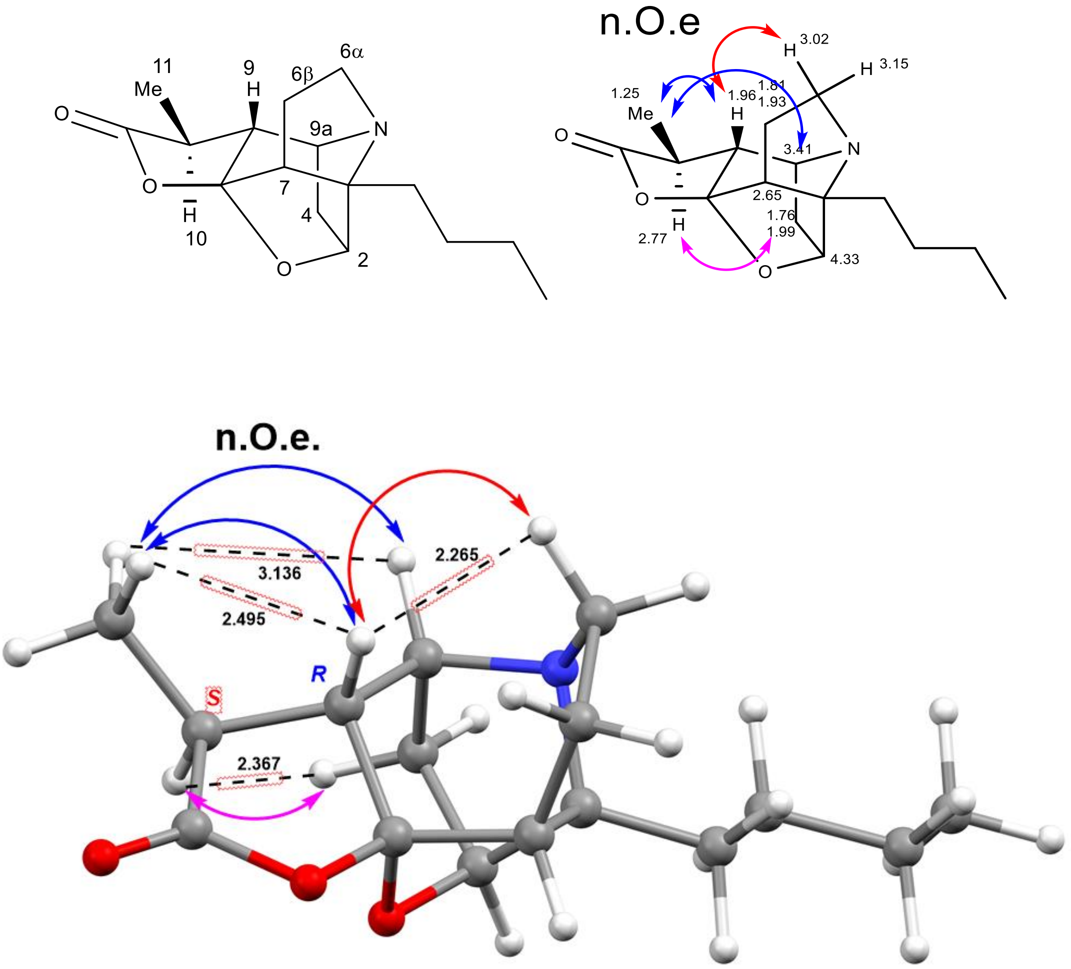

Supplementary Figure 10. Geometry of **12** optimized at b3lyp/6-31G* level and key correlations shown in NOESY spectrum

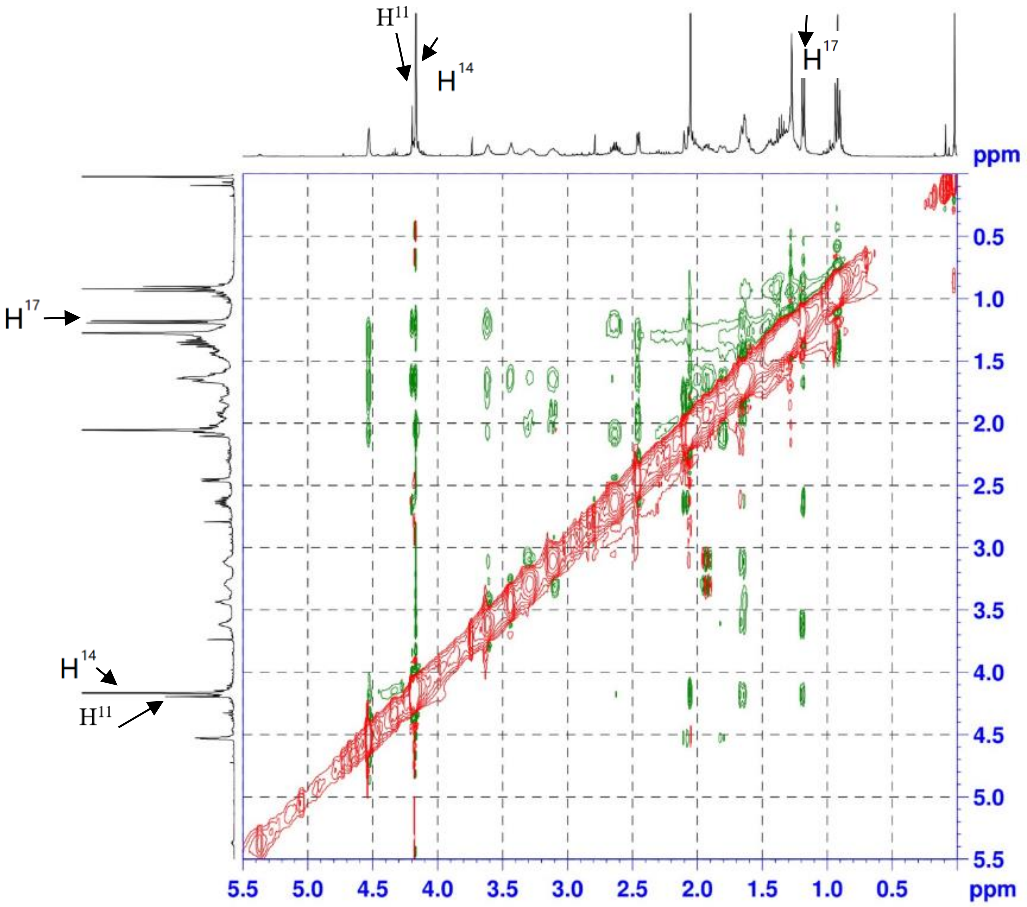

Supplementary Figure 11. NOESY spectrum of compound **31**

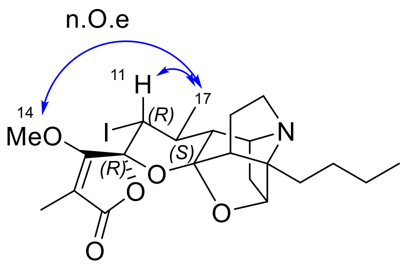

Supplementary Figure 12. The key correlations shown in NOESY spectrum

Supplementary Note 6

Single-Crystal X-Ray Diffraction Analysis of 30

X-Ray Crystallographic Data

CCDC 1962403 contains the crystallographic data for compound **30**. These data can be obtained free of charge from The Cambridge Crystallographic Data Centre via www.ccdc.cam.ac.uk/structures.

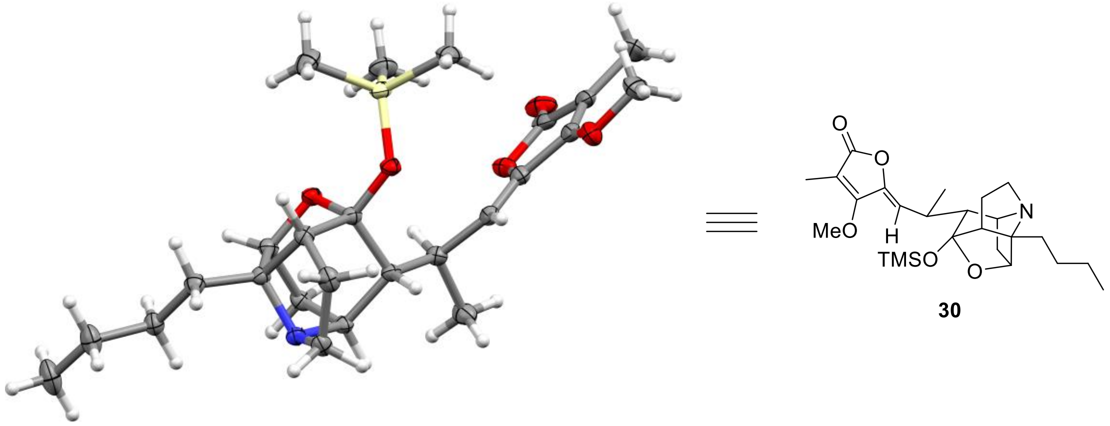

30

Supplementary Figure 13. X-ray crystal structure of **30**
(Thermals ellipsoids are shown with 50% probability.)

Supplementary Table 14. Crystal data and structure refinement for compound 30.

Identification code	**hxzh-1**
Empirical formula	$C_{25}H_{39}NO_5Si$
Formula weight	461.66
Temperature/K	100.00(10)
Crystal system	monoclinic
Space group	$P2_1$
a/Å	9.74827(11)
b/Å	10.52493(10)
c/Å	12.70946(12)
α/°	90
β/°	93.2925(9)
γ/°	90
Volume/Å^3	1301.84(2)
Z	2
ρ_{calc}g/cm^3	1.178
μ/mm^{-1}	1.066
F(000)	500.0
Crystal size/mm^3	1.091 × 0.518 × 0.424

Radiation	CuKα (λ = 1.54184)
2Θ range for data collection/°	6.966 to 134.898
Index ranges	$-11 \leq h \leq 11$, $-12 \leq k \leq 12$, $-15 \leq l \leq 15$
Reflections collected	18255
Independent reflections	4677 [R_{int} = 0.0209, R_{sigma} = 0.0138]
Data/restraints/parameters	4677/1/296
Goodness-of-fit on F^2	1.064
Final R indexes [I>=2σ (I)]	R_1 = 0.0280, wR_2 = 0.0774
Final R indexes [all data]	R_1 = 0.0281, wR_2 = 0.0775
Largest diff. peak/hole / e Å^{-3}	0.21/-0.18
Flack parameter	0.008(8)

Supplementary Table 15. Fractional Atomic Coordinates (×10^4) and Equivalent Isotropic Displacement Parameters (Å^2×10^3) for compound 30. U_{eq} is defined as 1/3 of of the trace of the orthogonalised U_{IJ} tensor.

Atom	*x*	*y*	*z*	U(eq)
Si01	3710.5(5)	7457.1(5)	6695.8(4)	21.21(14)
O002	4525.4(14)	6682.7(13)	7686.8(10)	19.0(3)
O003	7963.9(15)	6223.4(14)	5688.2(12)	24.6(3)
O004	3936.8(14)	4668.6(13)	7084.4(10)	19.4(3)
O005	8104.6(16)	9100.1(15)	7120.9(12)	30.3(3)
O006	8682.2(17)	6610.9(19)	4067.3(12)	35.3(4)
N007	4496.2(17)	3286.6(16)	9354.1(12)	18.4(3)
C008	7807(2)	6958(2)	6584.4(16)	21.6(4)
C009	4434.1(19)	5417.1(17)	7977.0(14)	17.1(4)
C00A	5859.4(19)	4896.2(17)	8368.1(14)	16.8(4)
C00B	5648.5(19)	3472.2(18)	8659.2(15)	17.6(4)
C00C	3279.3(19)	3689.3(18)	8672.1(14)	17.9(4)
C00D	6957.7(19)	5085.9(18)	7556.4(15)	19.6(4)
C00E	5075(2)	2743.3(18)	7687.9(15)	20.4(4)
C00F	3348(2)	5144.3(18)	8778.6(15)	19.2(4)
C00G	7338.7(19)	6464.7(19)	7456.5(15)	20.2(4)
C00H	4539(2)	4133(2)	10277.3(15)	22.6(4)
C00I	3698(2)	3426.0(19)	7526.9(14)	19.4(4)
C00J	8440(2)	7027(2)	4925.2(16)	26.7(5)
C00K	1956(2)	1622(2)	8861.6(16)	23.1(4)
C00L	1967(2)	3059.5(19)	8996.5(16)	22.7(4)
C00M	8195(2)	8257(2)	6342.6(17)	24.2(4)
C00N	8567(2)	8310(2)	5334.0(17)	27.7(5)

C00O	4273(3)	9135(2)	6879.8(18)	31.2(5)
C00P	8274(2)	4316(2)	7827.5(18)	29.3(5)
C00Q	3802(2)	5386.8(19)	9932.5(15)	22.7(4)
C00R	4228(3)	6875(2)	5395.2(17)	32.3(5)
C00S	1813(2)	7363(2)	6779.4(19)	34.1(5)
C00T	737(2)	985(2)	9336(2)	33.4(5)
C00U	9080(3)	9347(3)	4649(2)	40.4(6)
C00V	727(3)	-445(2)	9133(2)	41.0(6)
C00W	8327(3)	10420(2)	6901(2)	42.8(6)

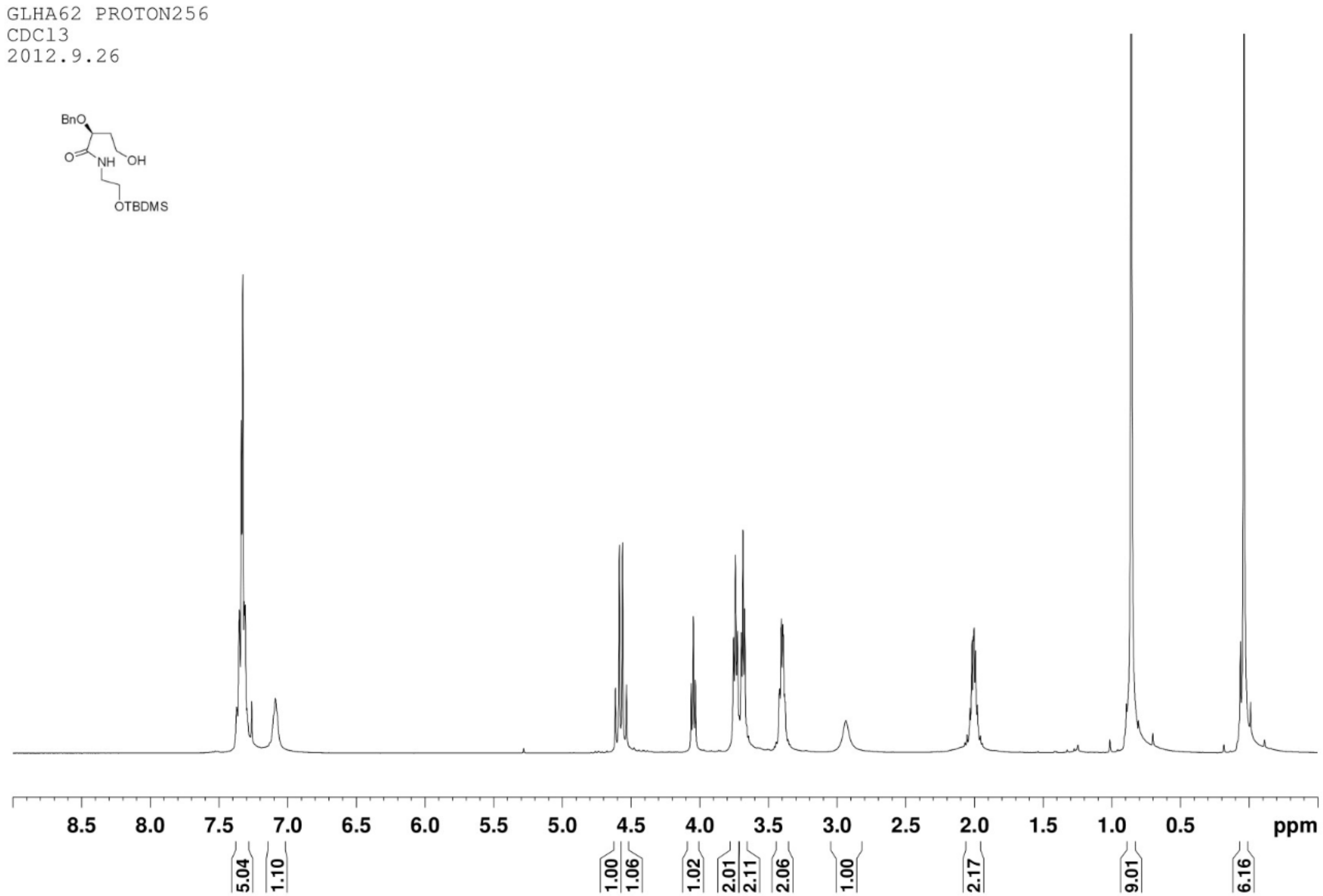

Supplementary Figure 14. ^{1}H NMR spectrum of compound **20**

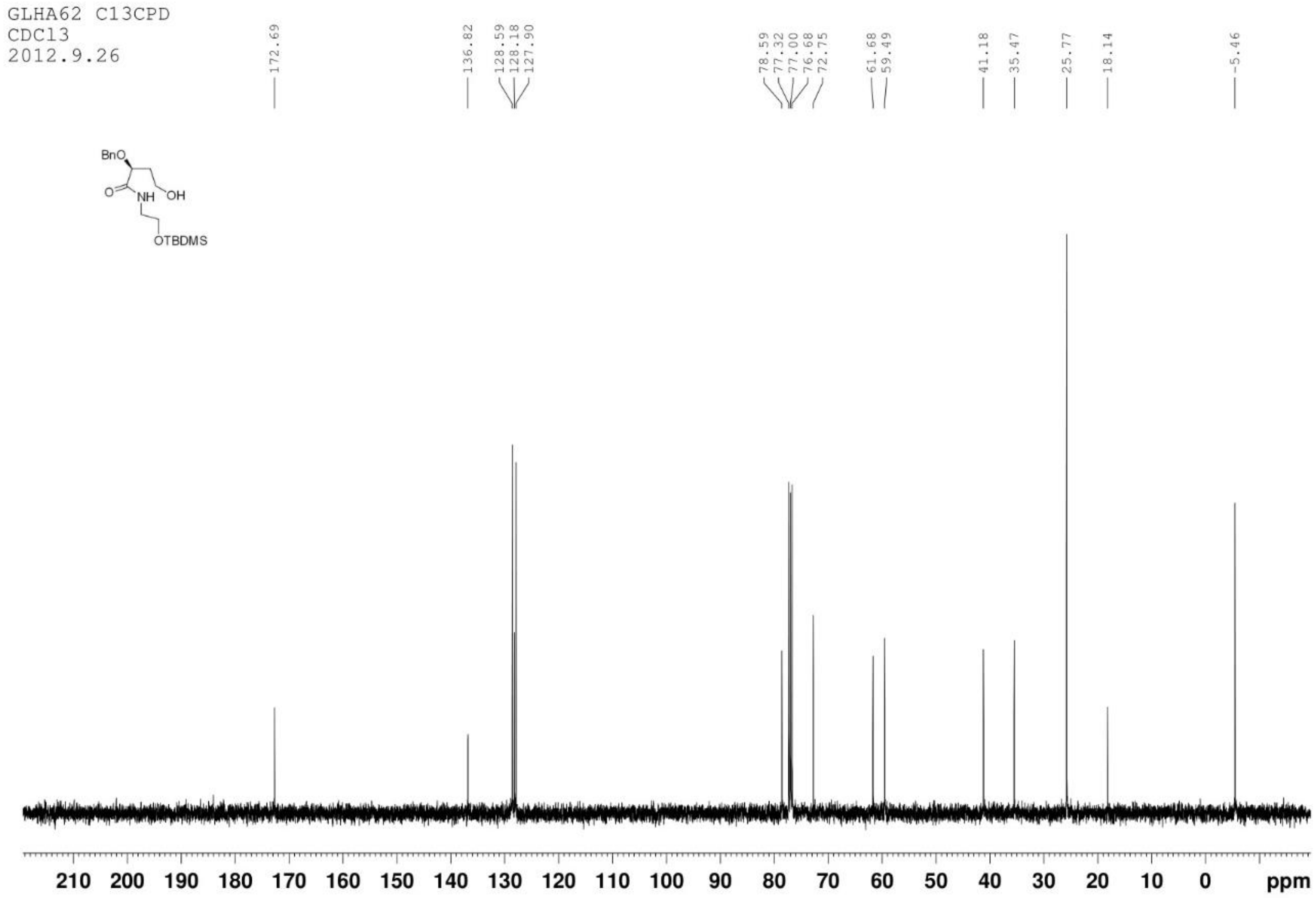

Supplementary Figure 15. ^{13}C NMR spectrum of compound **20**

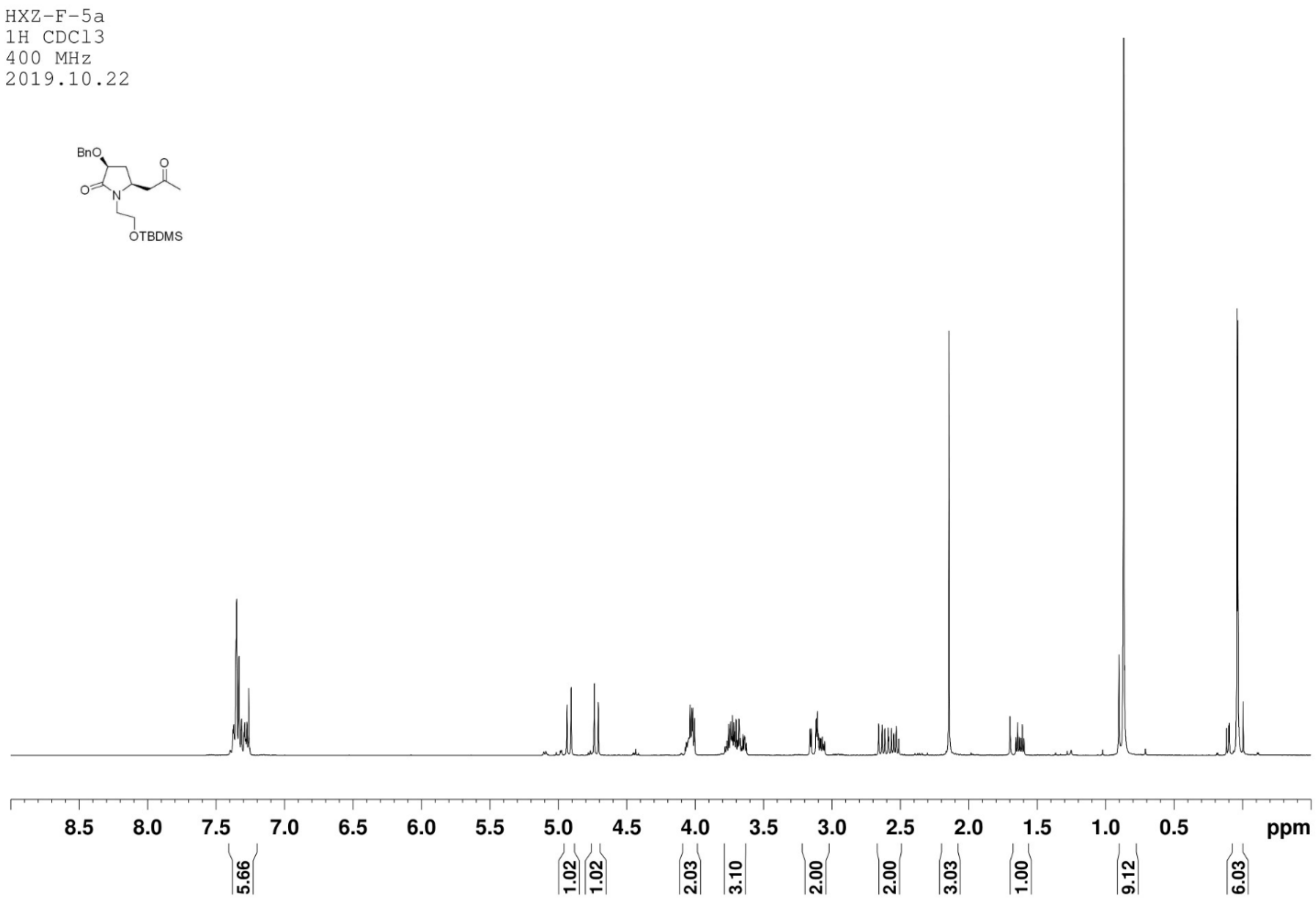

Supplementary Figure 16. ^{1}H NMR spectrum of compound ***cis*-17**

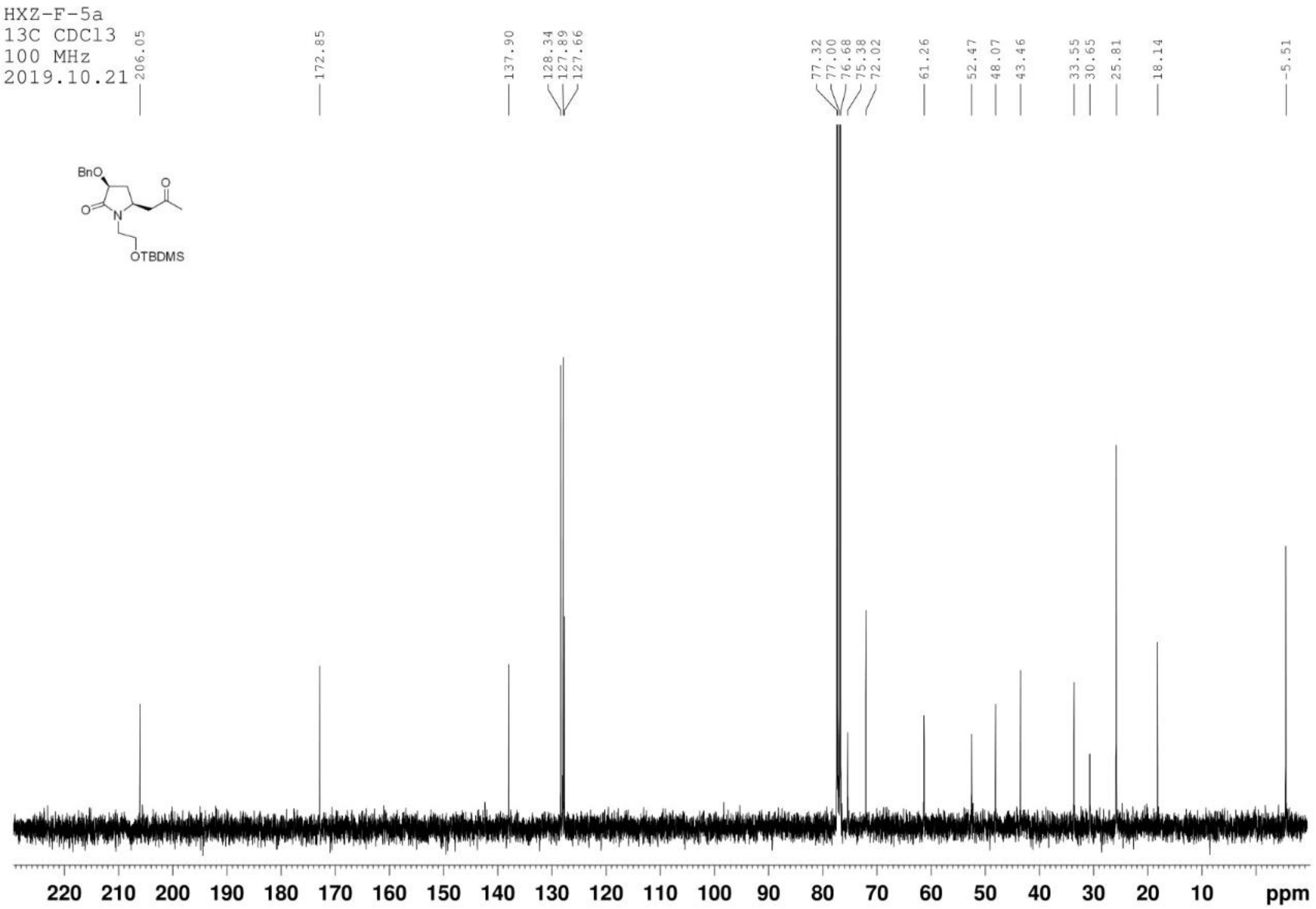

Supplementary Figure 17. ^{13}C NMR spectrum of compound ***cis*-17**

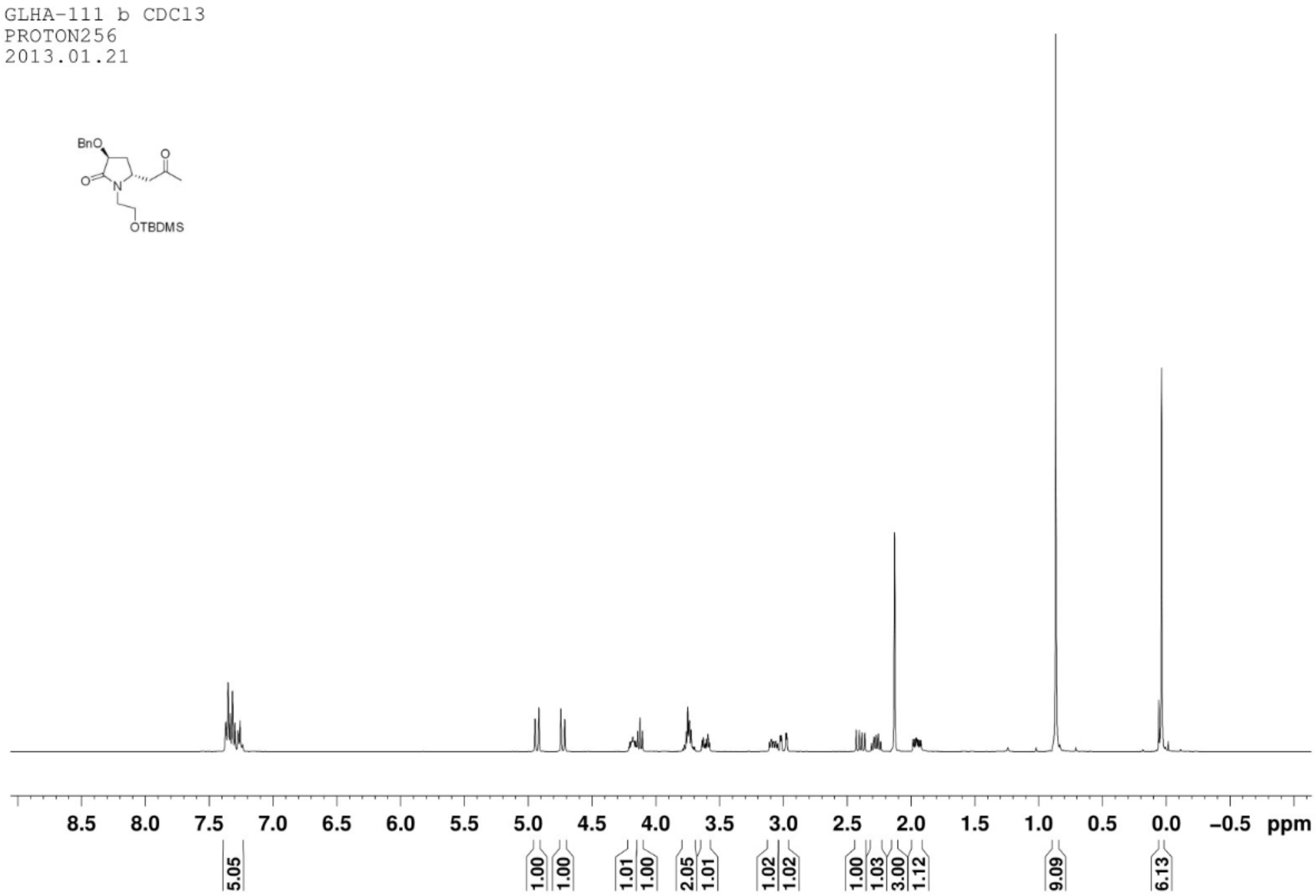

Supplementary Figure 18. ^{1}H NMR spectrum of compound ***trans*-17**

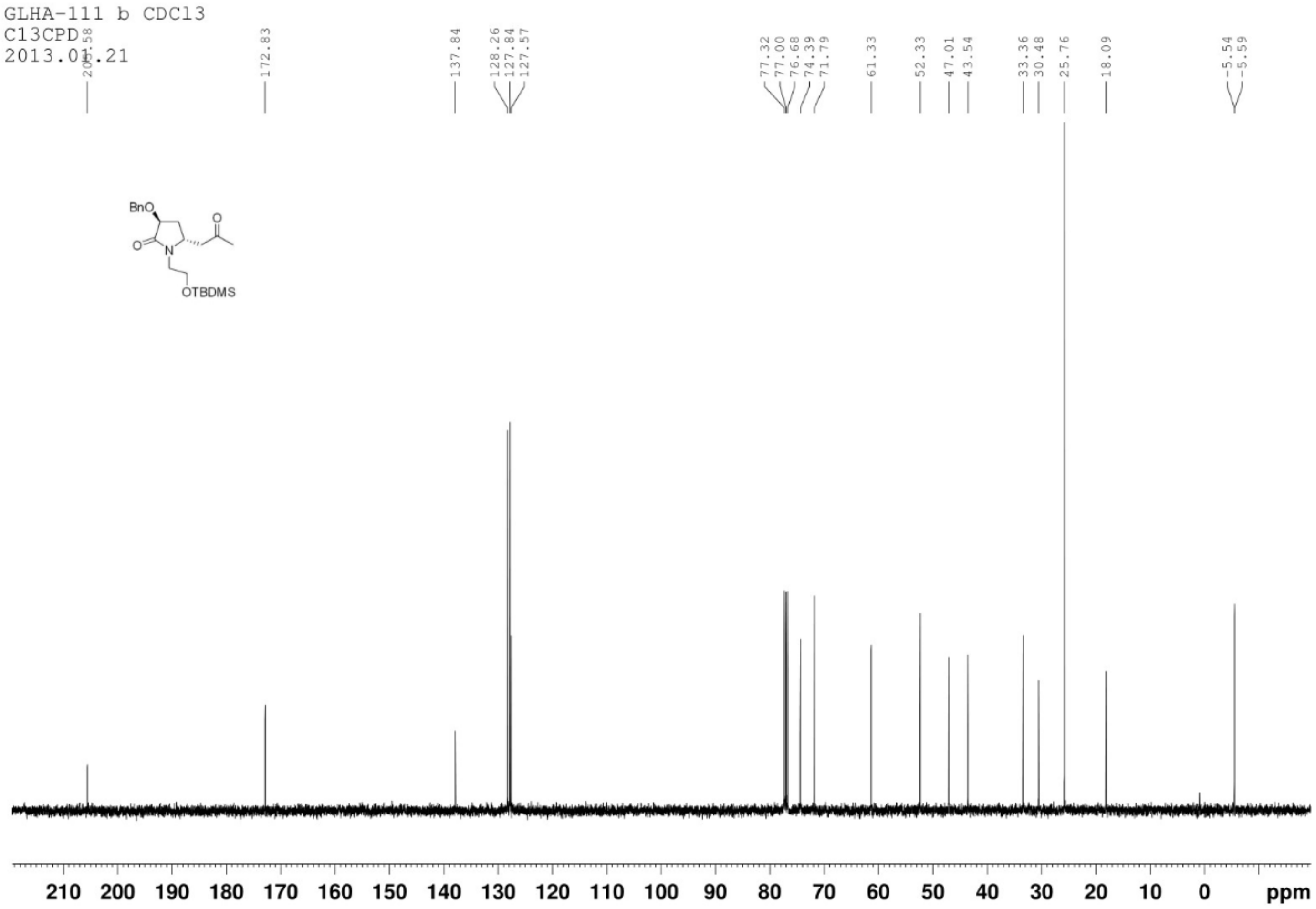

Supplementary Figure 19. ^{13}C NMR spectrum of compound ***trans*-17**

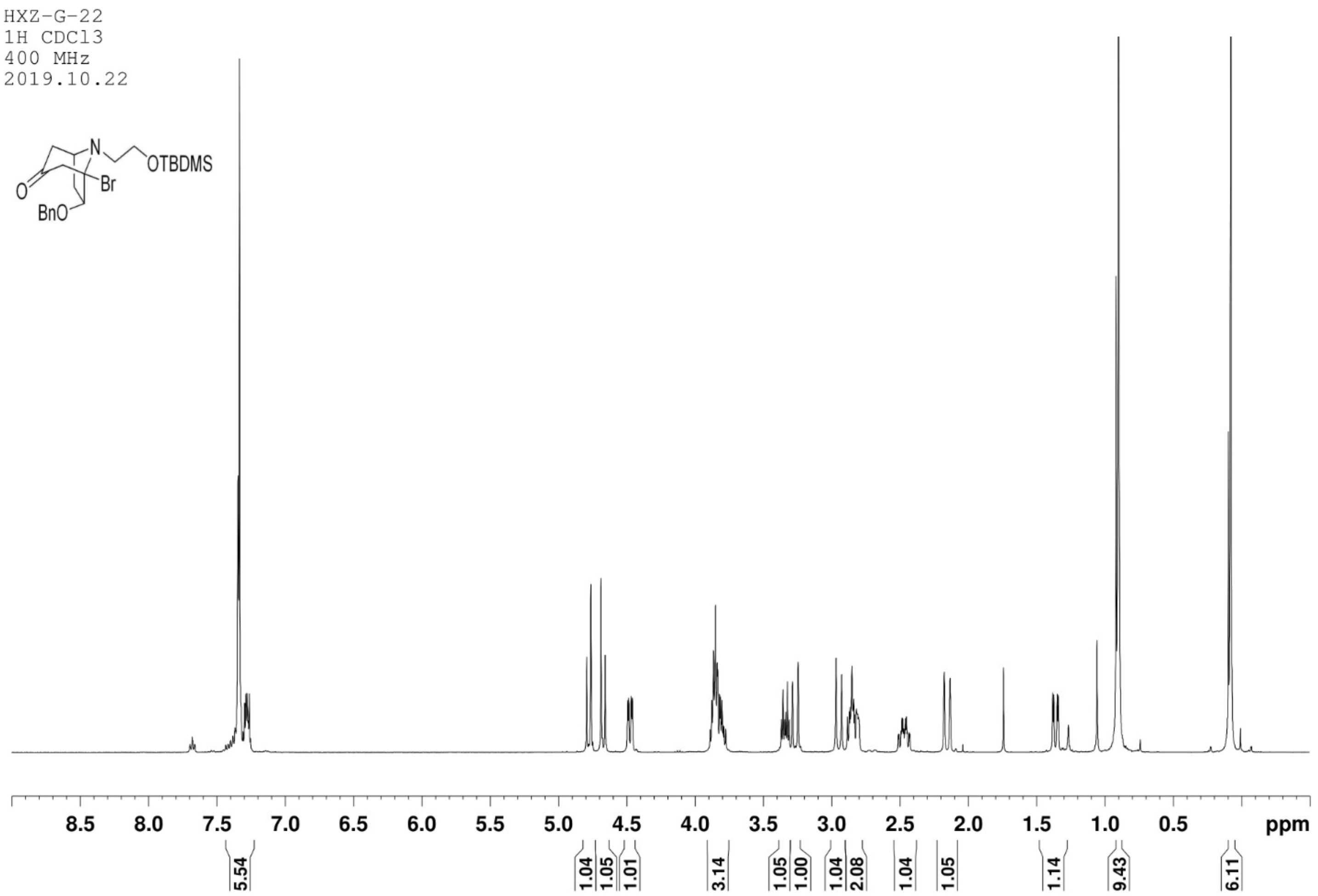

Supplementary Figure 20. ^{1}H NMR spectrum of compound **16**

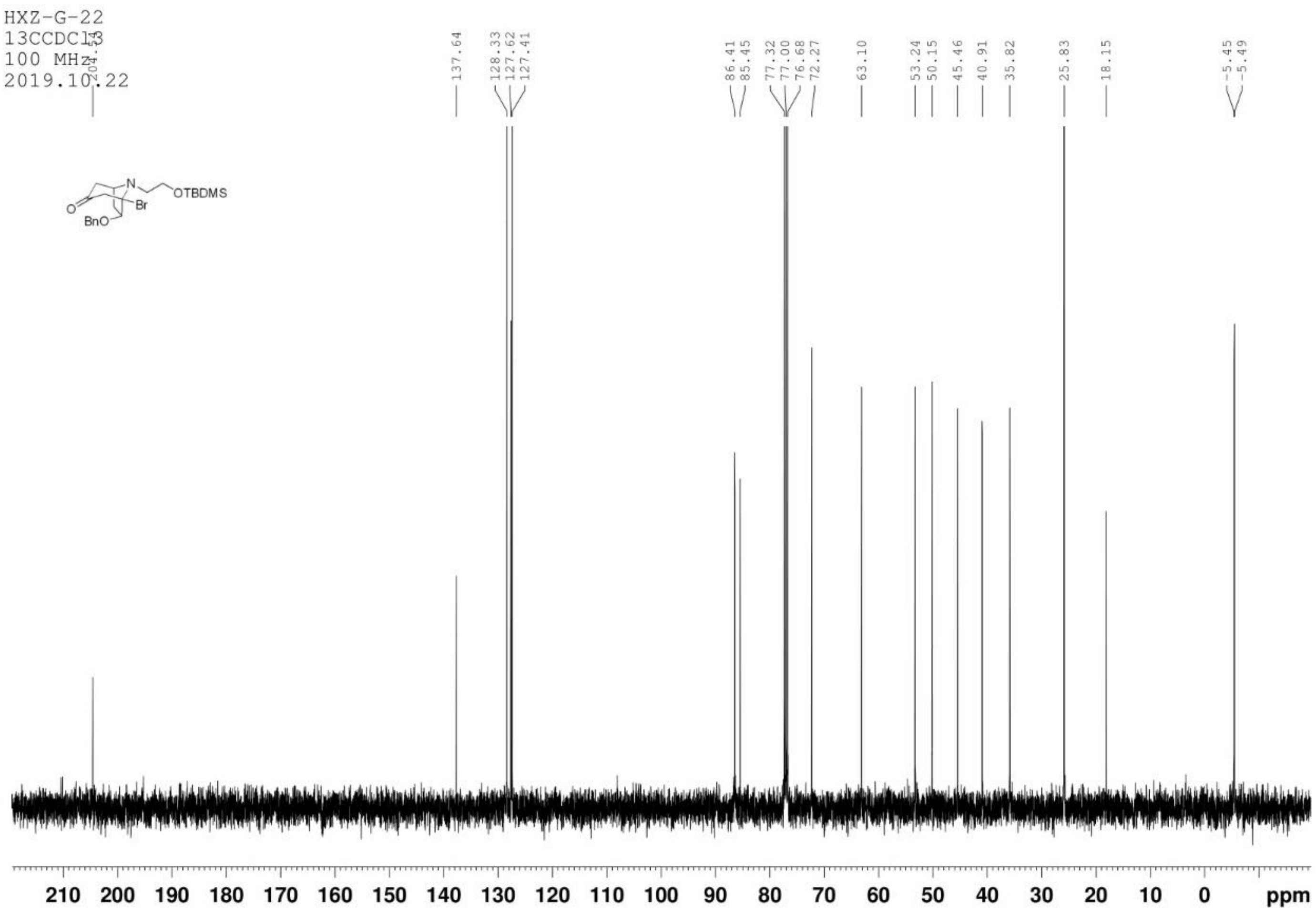

Supplementary Figure 21. ^{13}C NMR spectrum of compound **16**

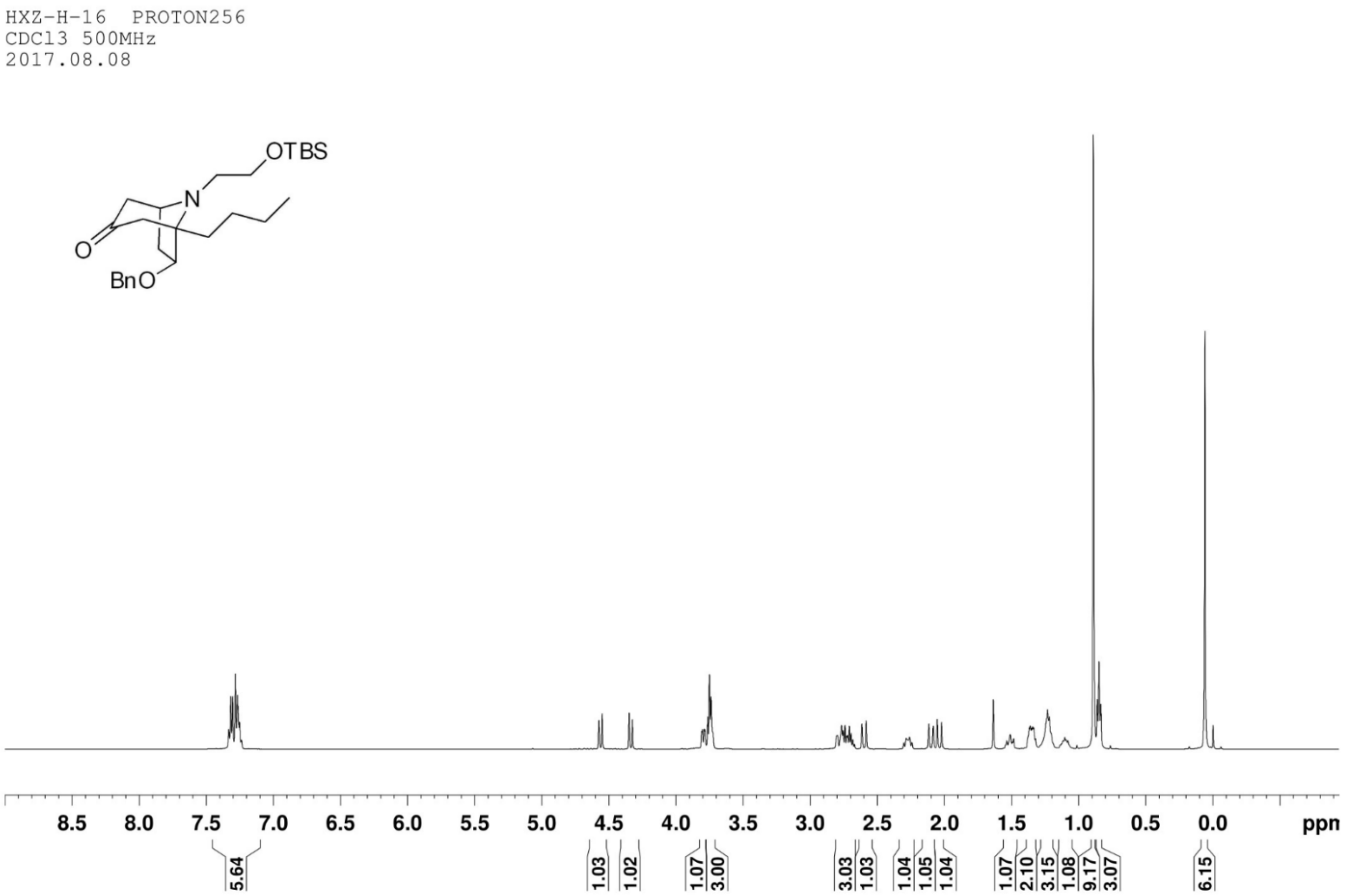

Supplementary Figure 22. ^{1}H NMR spectrum of compound **15**

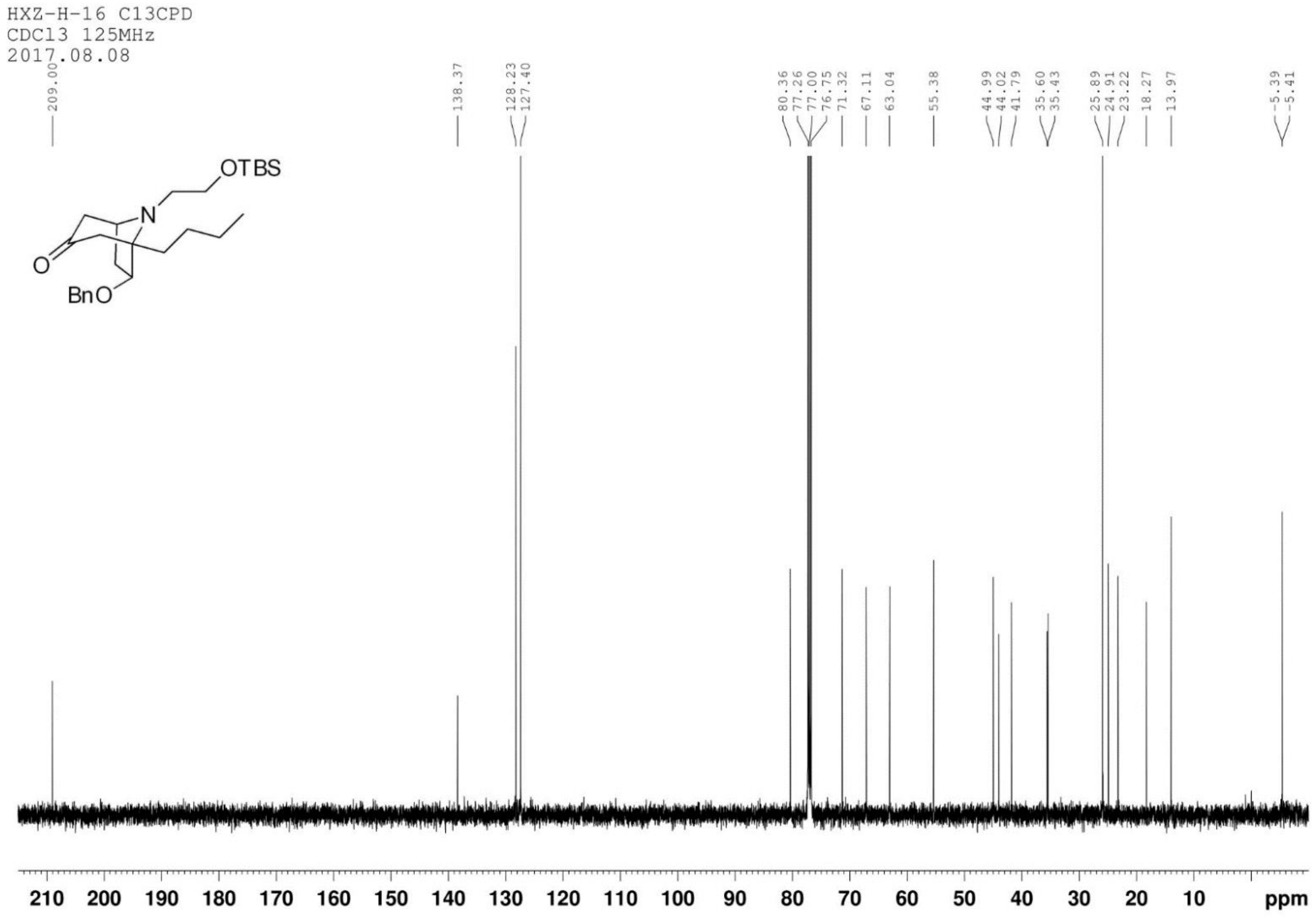

Supplementary Figure 23. ^{13}C NMR spectrum of compound **15**

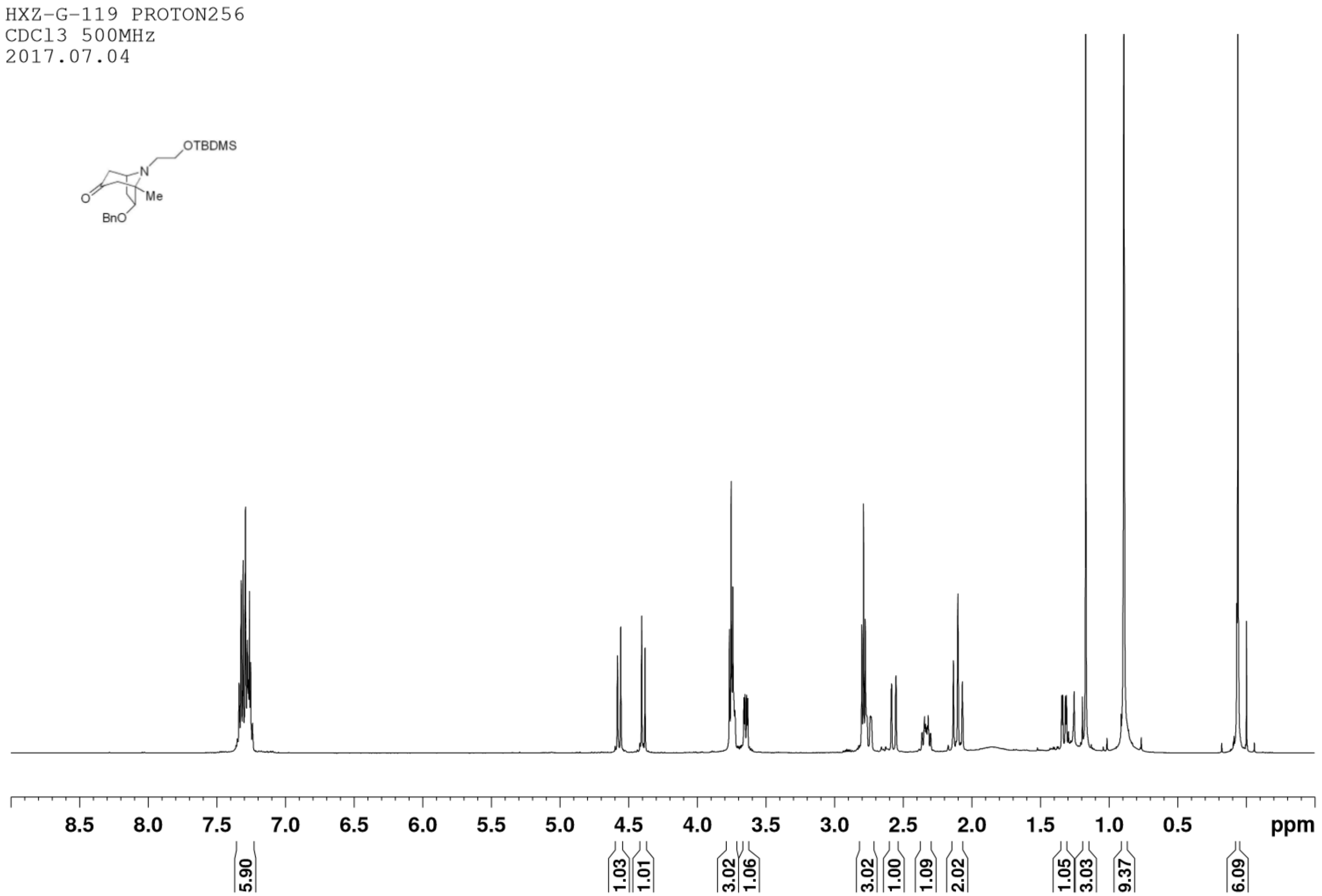

Supplementary Figure 24. ^{1}H NMR spectrum of compound **15a**

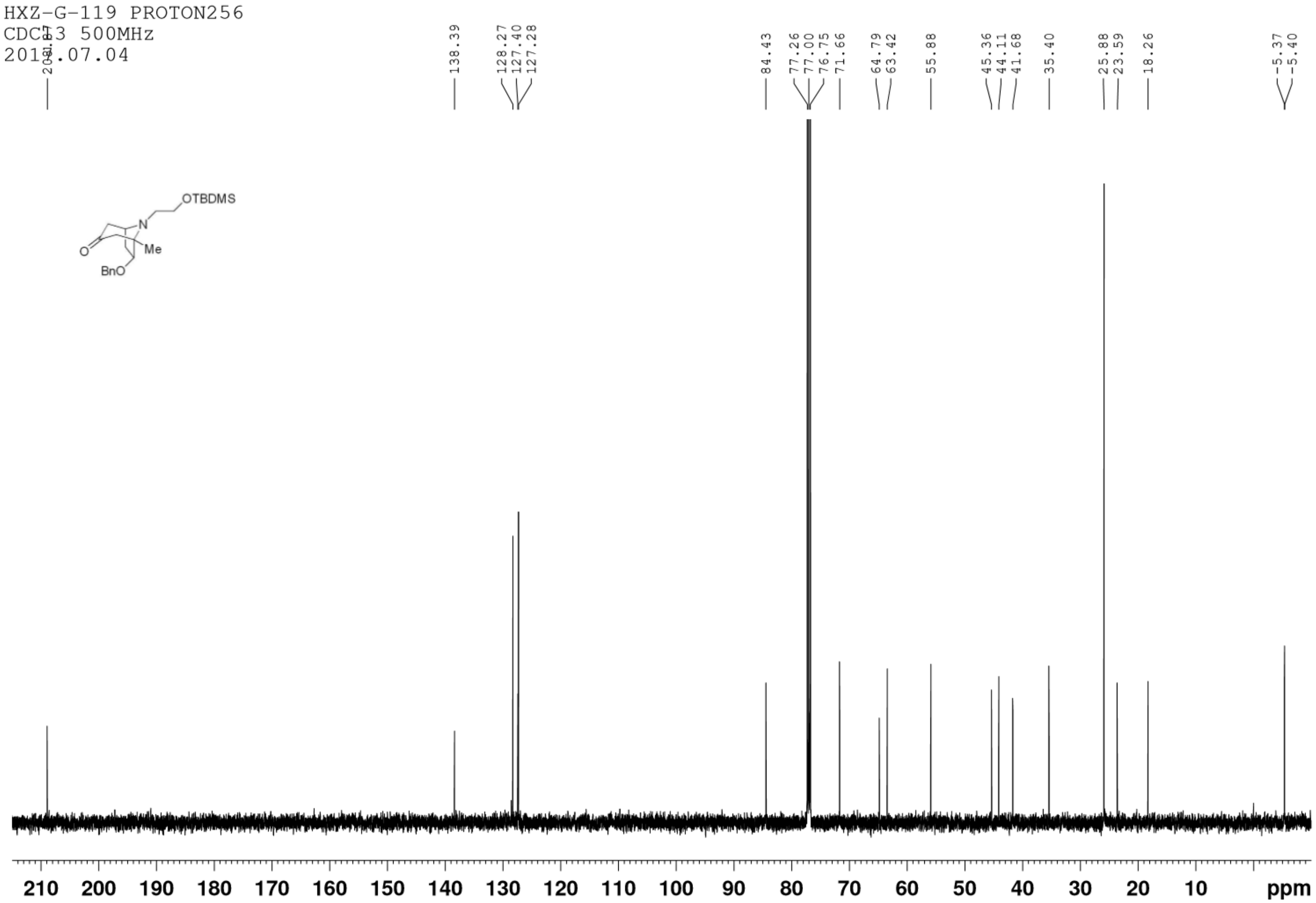

Supplementary Figure 25. ^{13}C NMR spectrum of compound **15a**

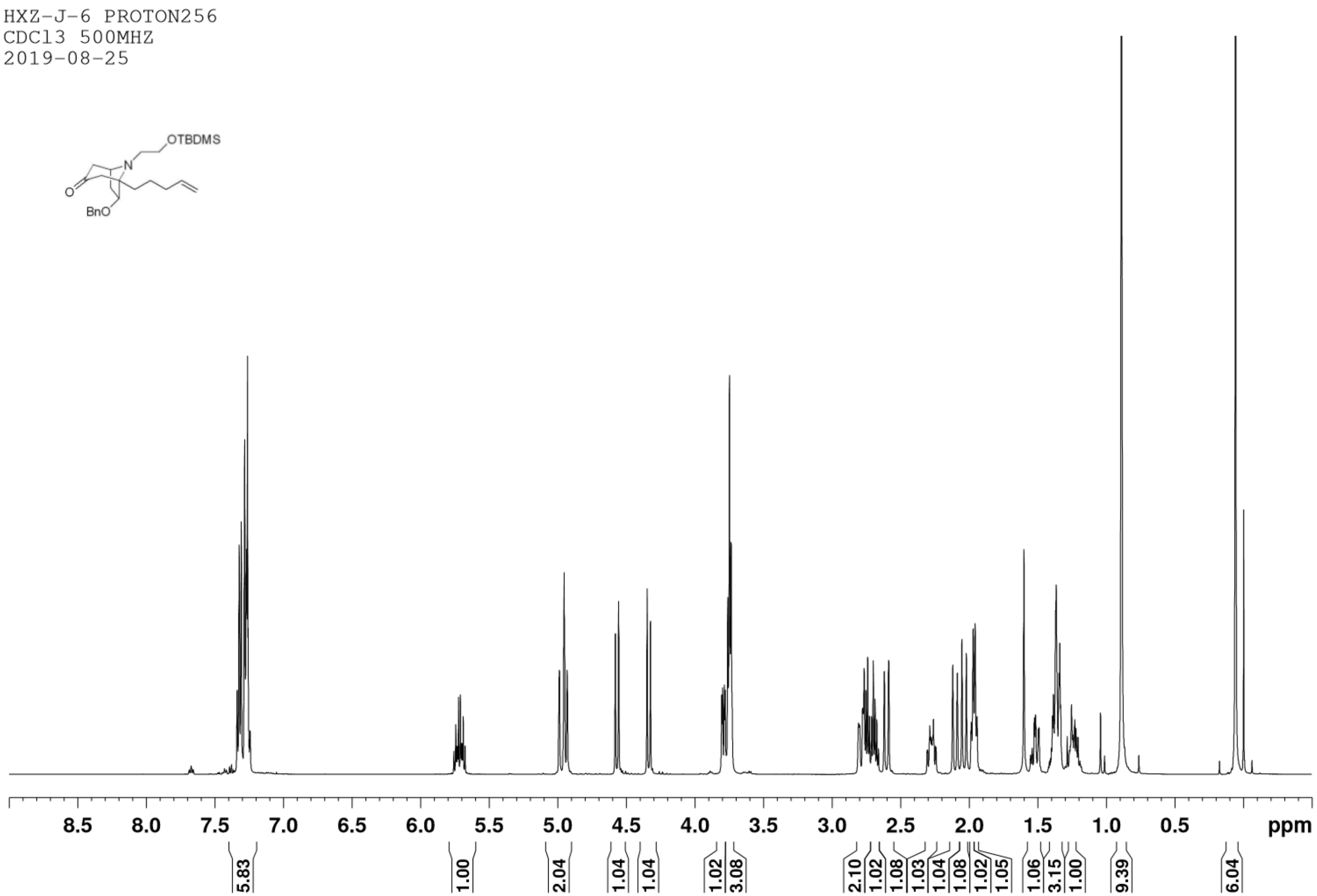

Supplementary Figure 26. ^{1}H NMR spectrum of compound **15b**

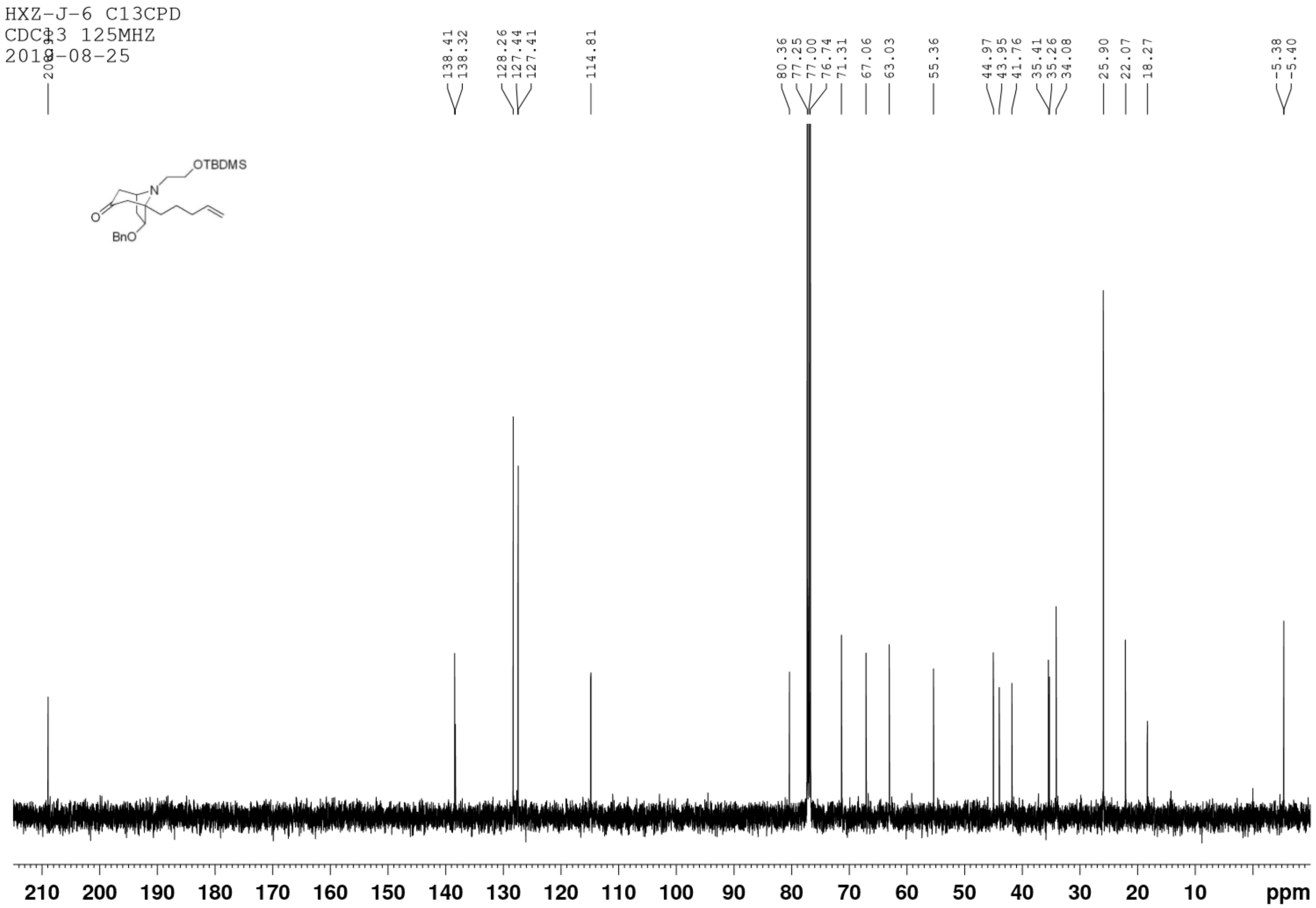

Supplementary Figure 27. ^{13}C NMR spectrum of compound **15b**

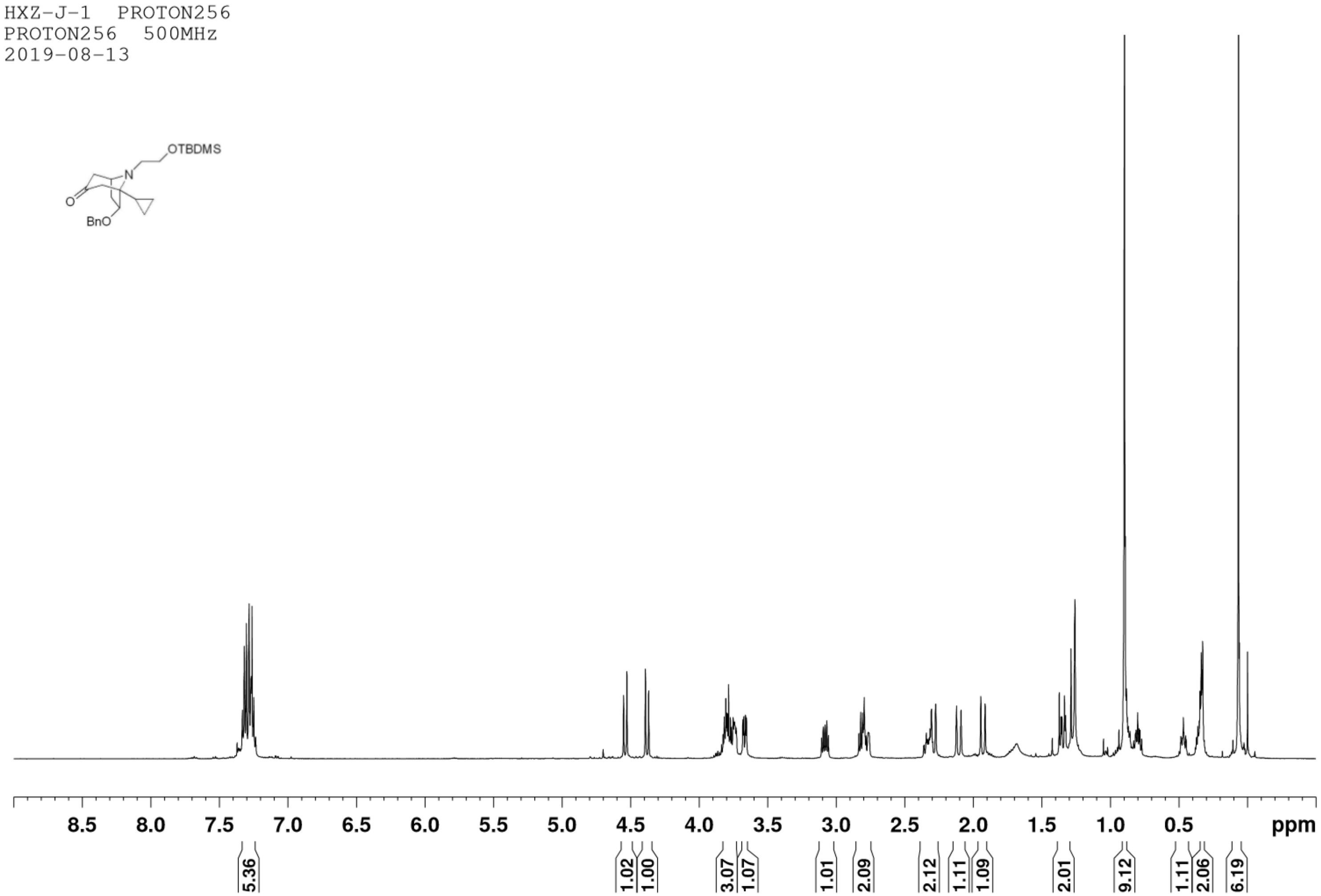

Supplementary Figure 28. ^{1}H NMR spectrum of compound **15c**

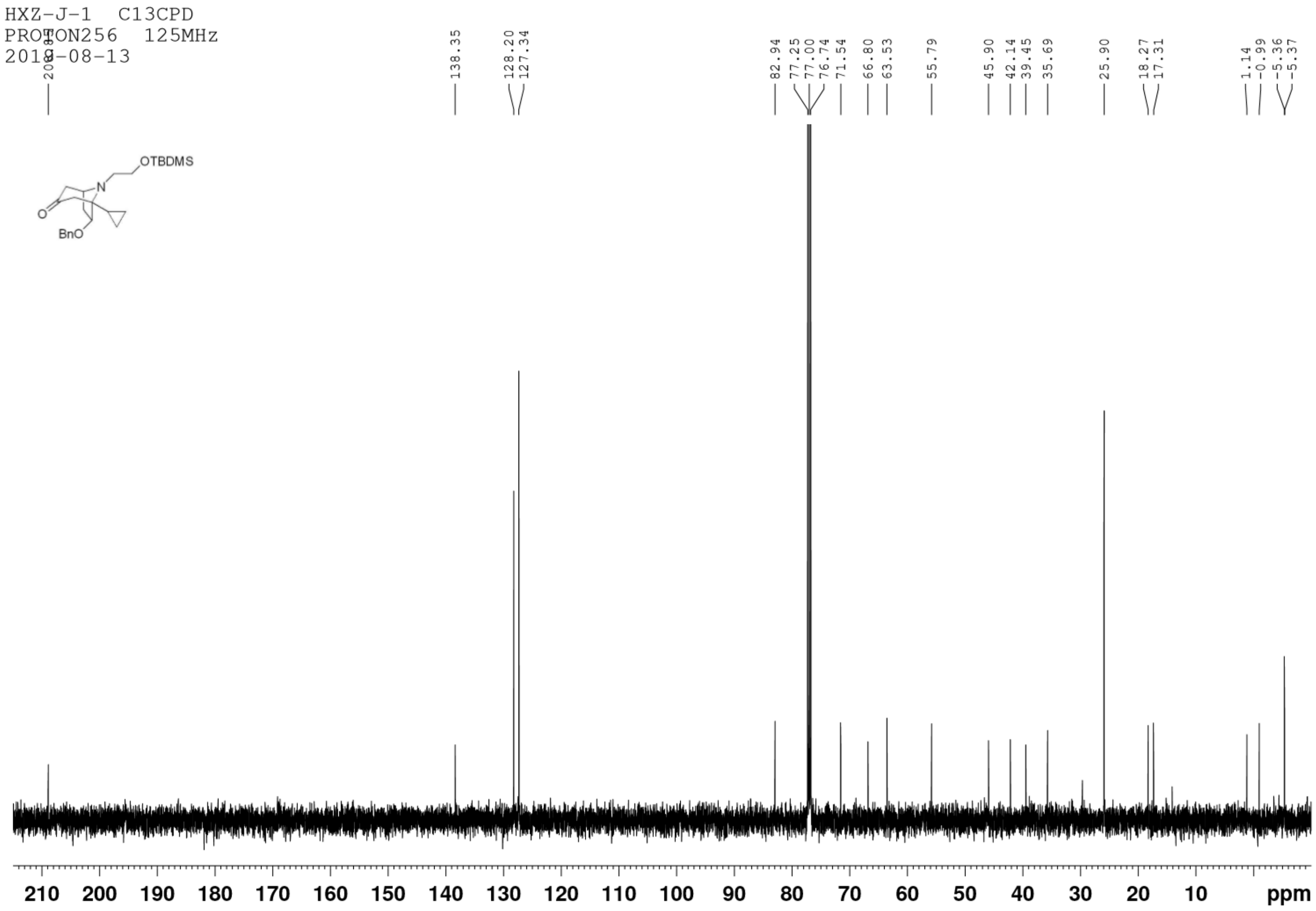

Supplementary Figure 29. ^{13}C NMR spectrum of compound **15c**

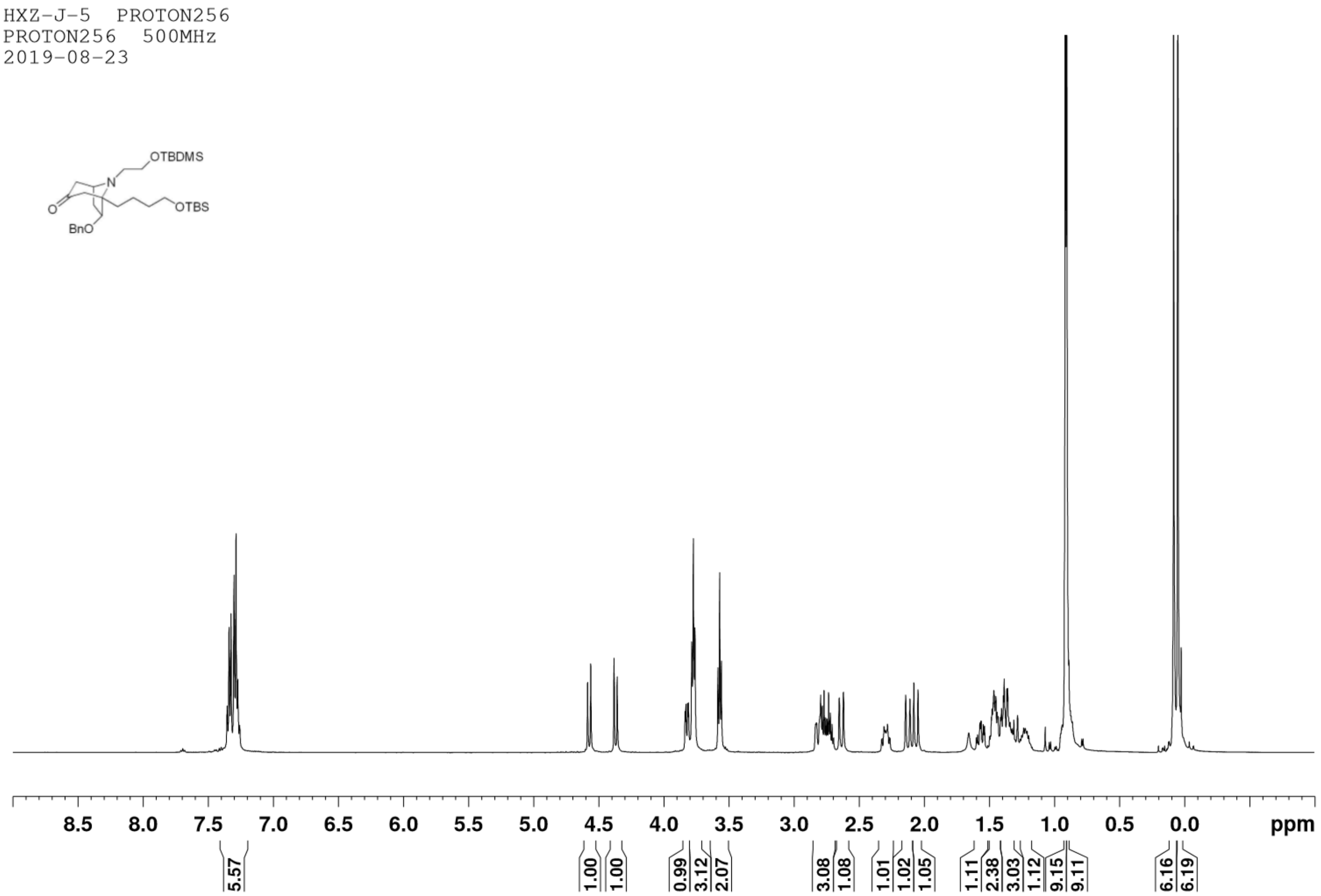

Supplementary Figure 30. ^{1}H NMR spectrum of compound **15d**

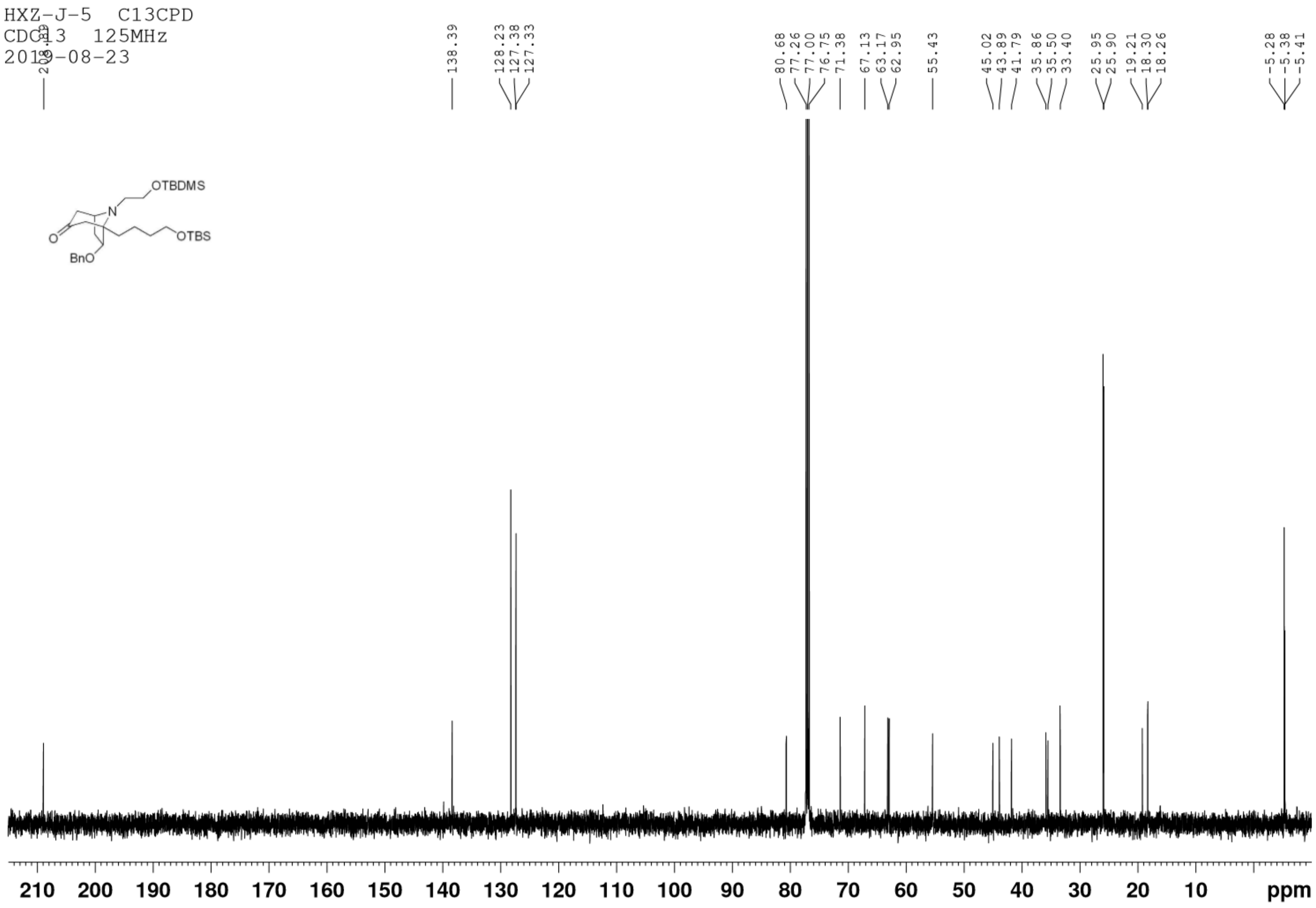

Supplementary Figure 31. ^{13}C NMR spectrum of compound **15d**

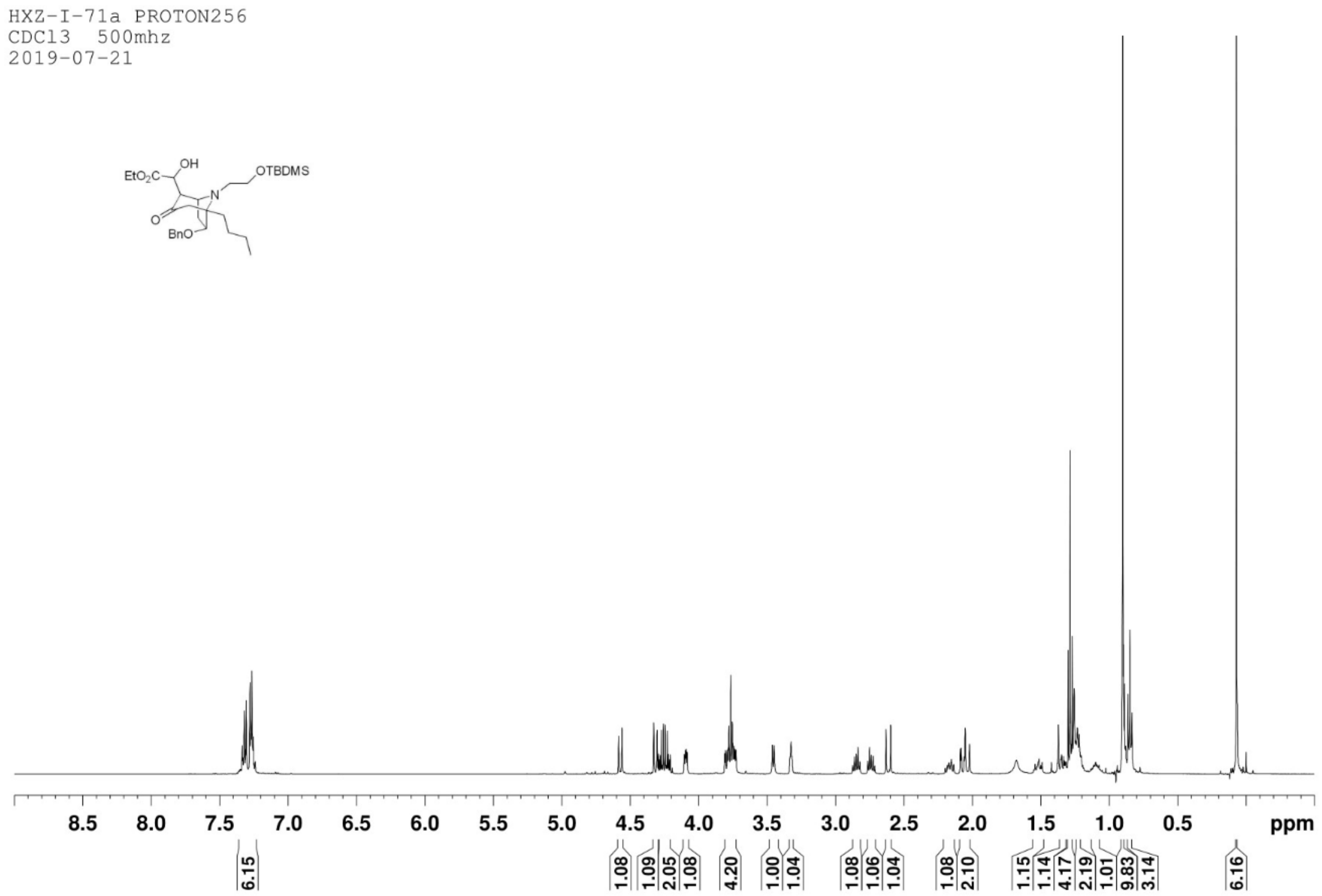

Supplementary Figure 32. ^{1}H NMR spectrum of compound **23-1 or 23-2**

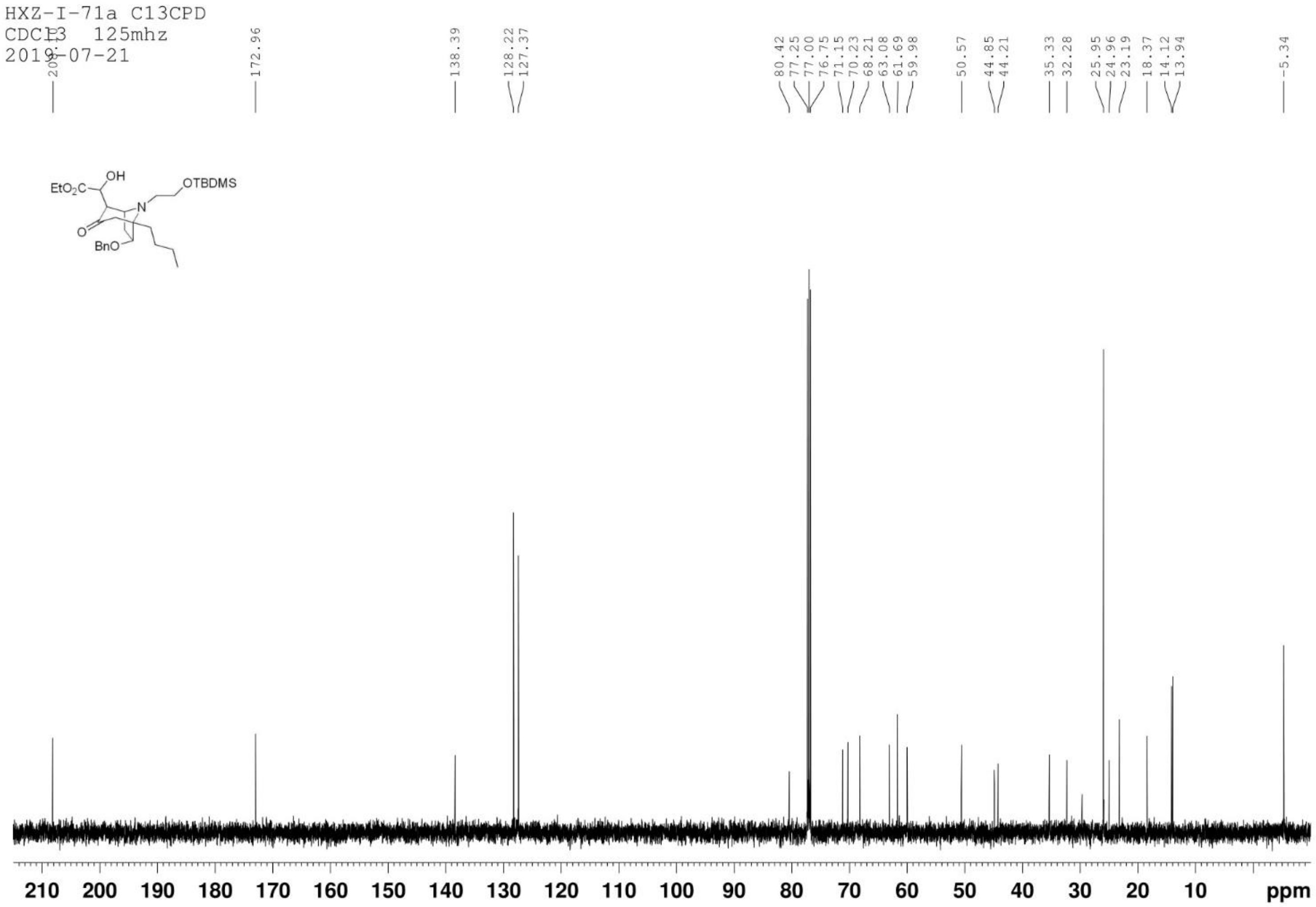

Supplementary Figure 33. ^{13}C NMR spectrum of compound **23-1 or 23-2**

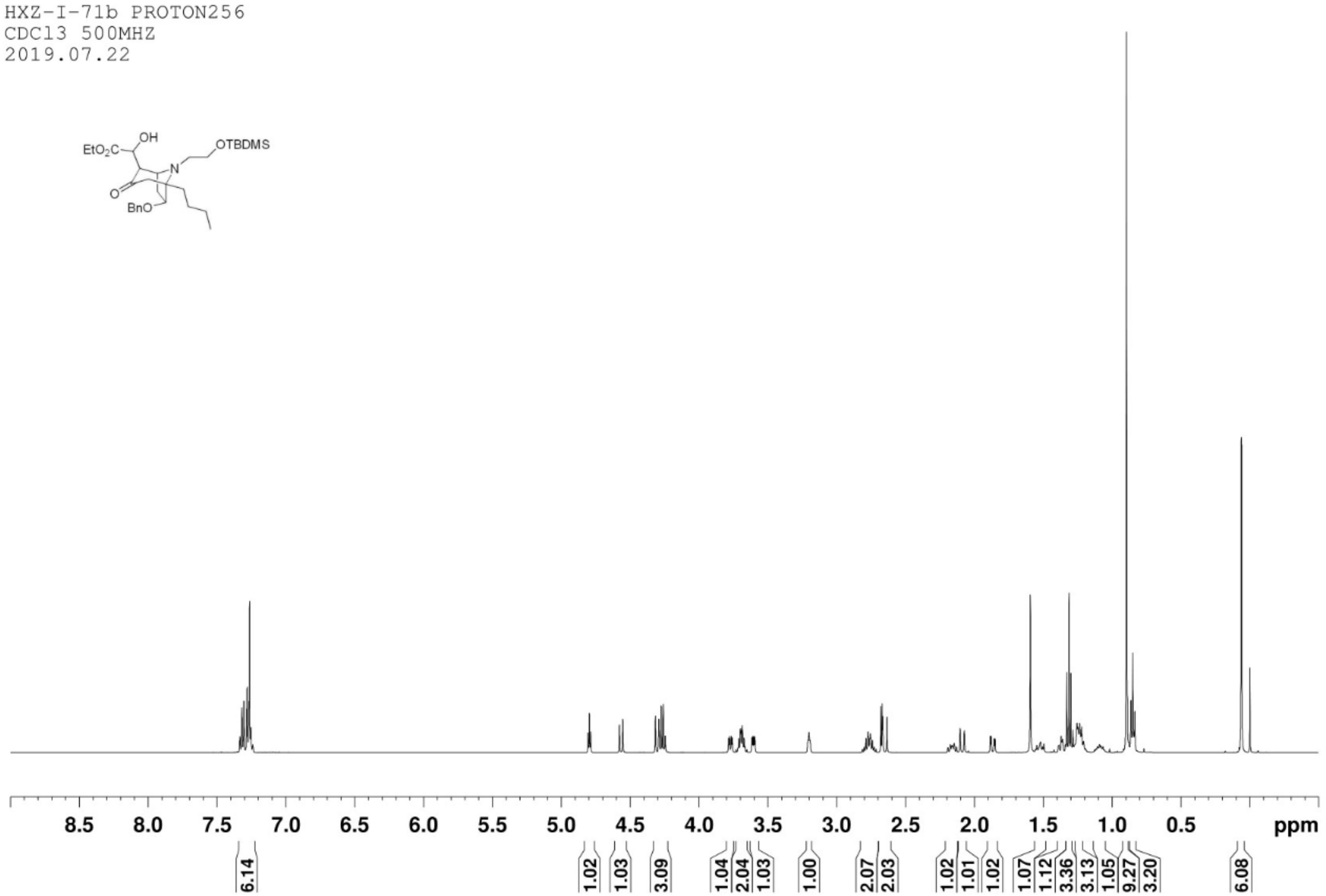

Supplementary Figure 34. ^{1}H NMR spectrum of compound **23-1 or 23-2**

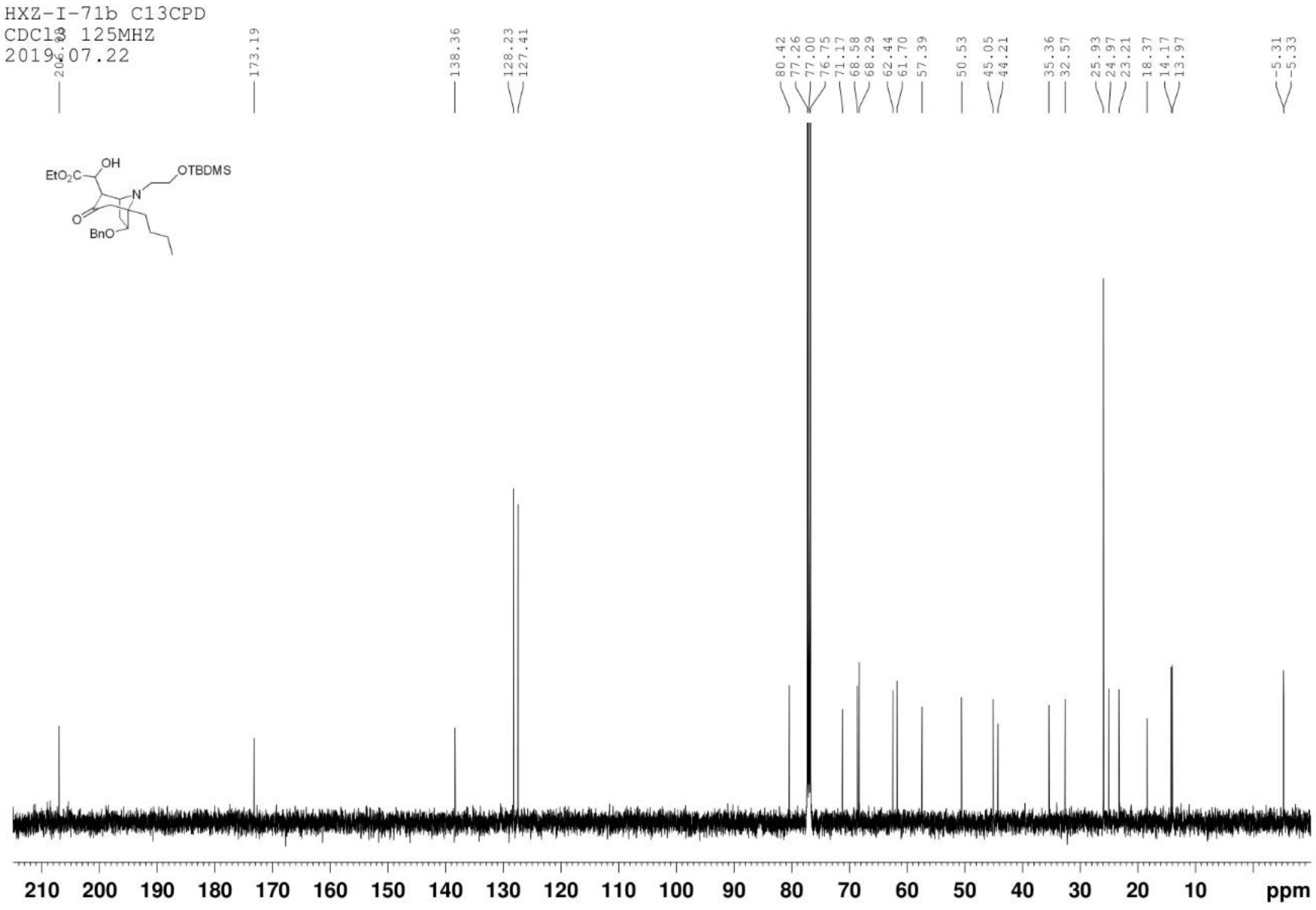

Supplementary Figure 35 ^{13}C NMR spectrum of compound **23-1 or 23-2**

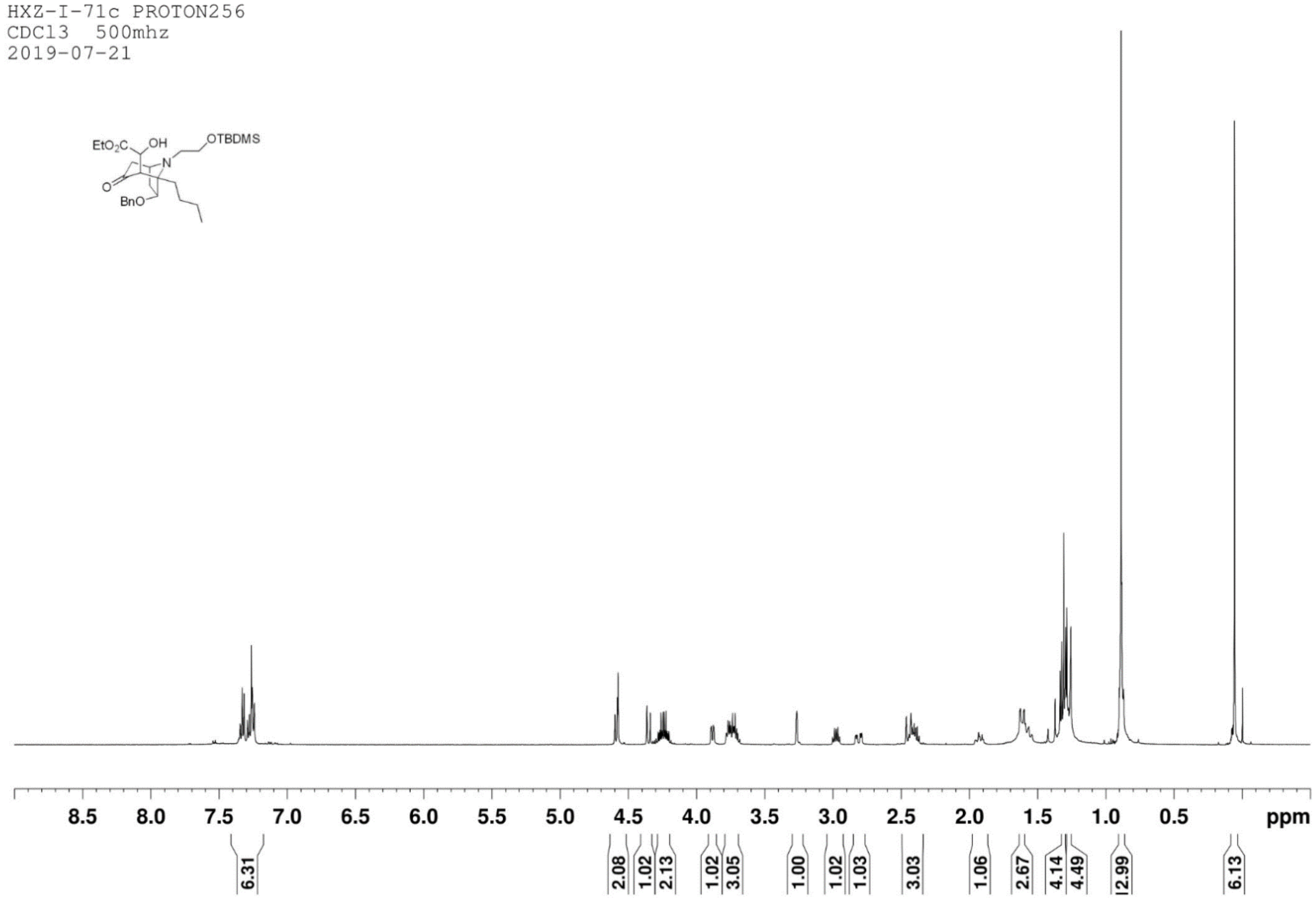

Supplementary Figure 36. ^{1}H NMR spectrum of compound **23a**

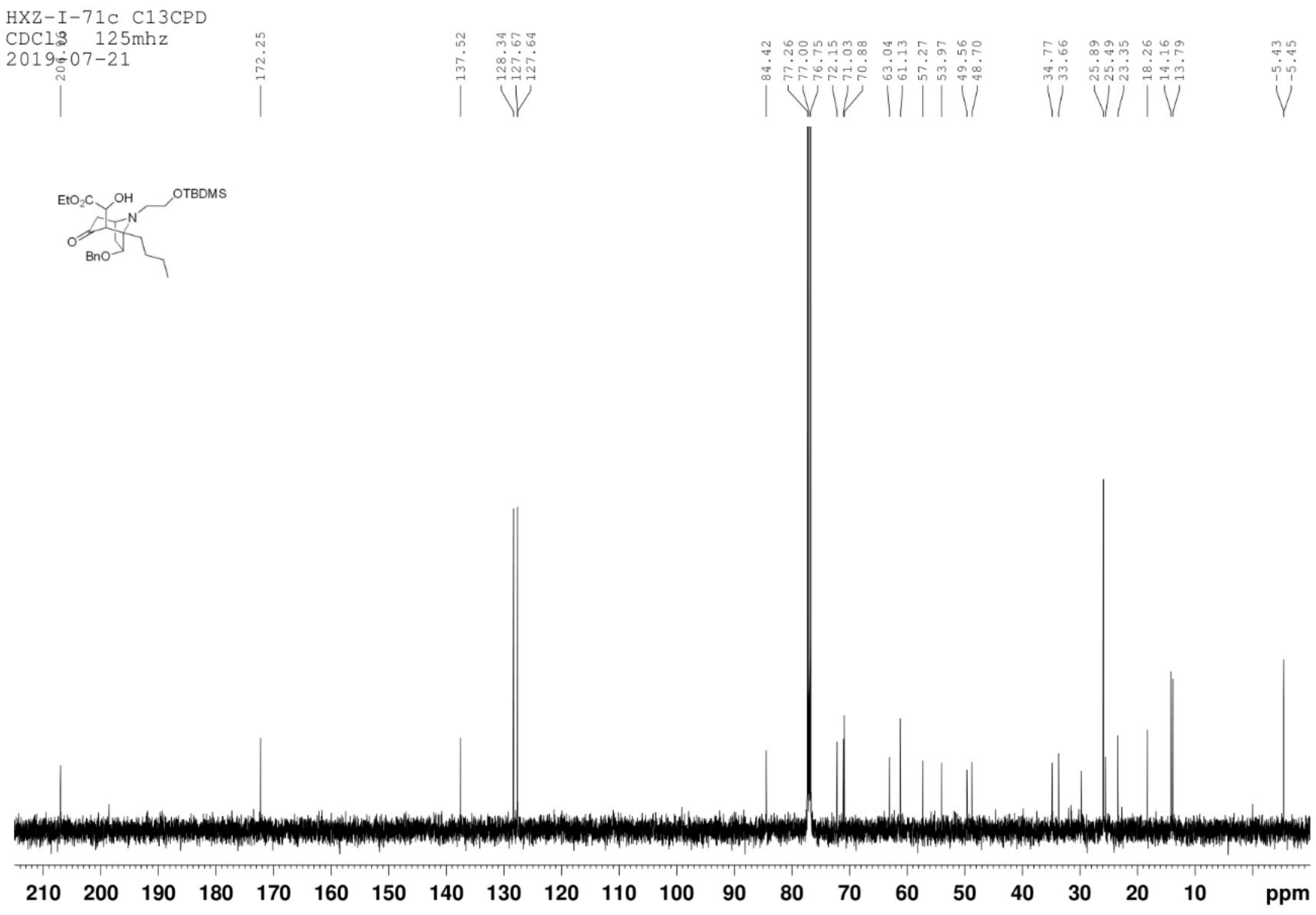

Supplementary Figure 37. ^{13}C NMR spectrum of compound **23a**

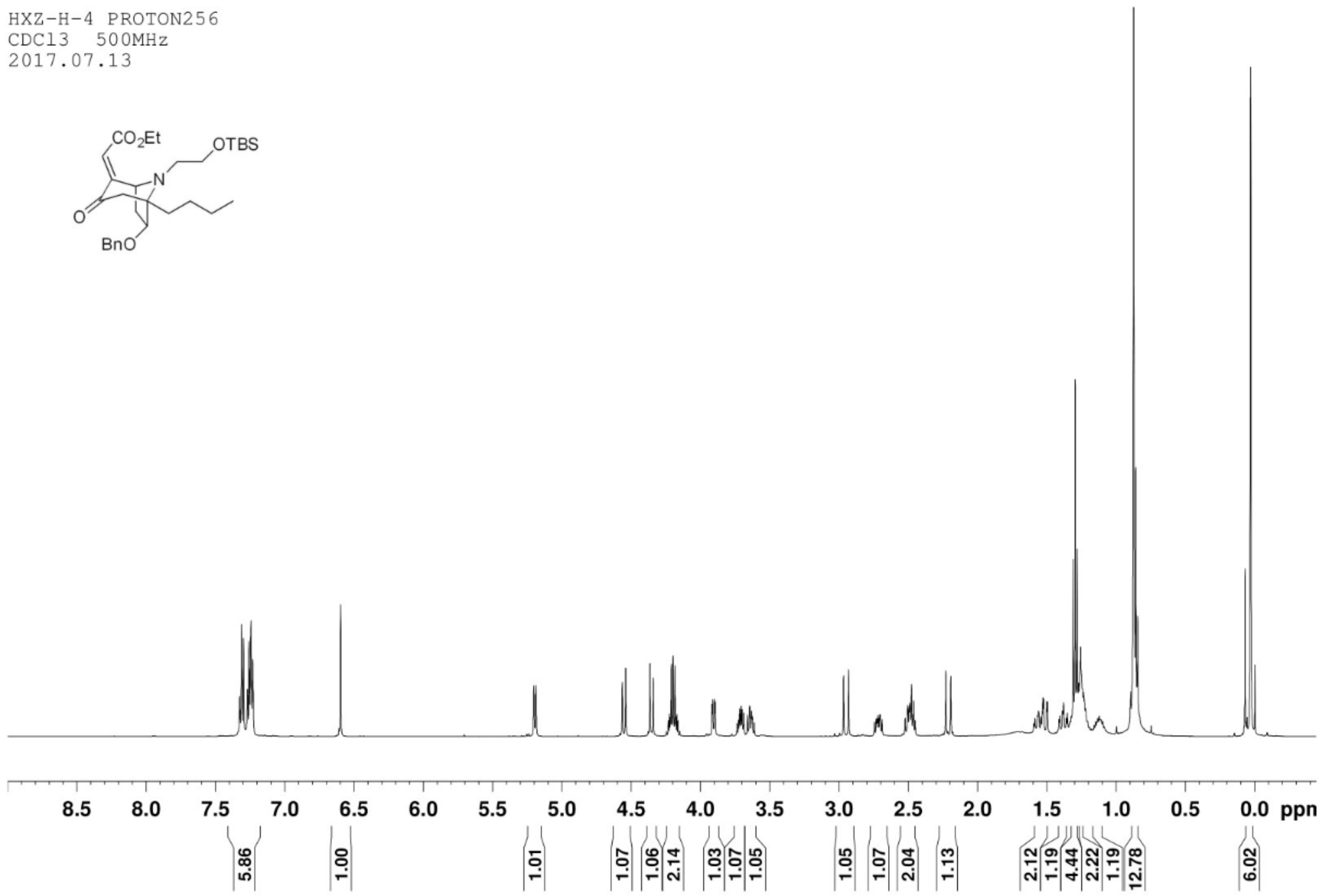

Supplementary Figure 38. ^{1}H NMR spectrum of compound **24**

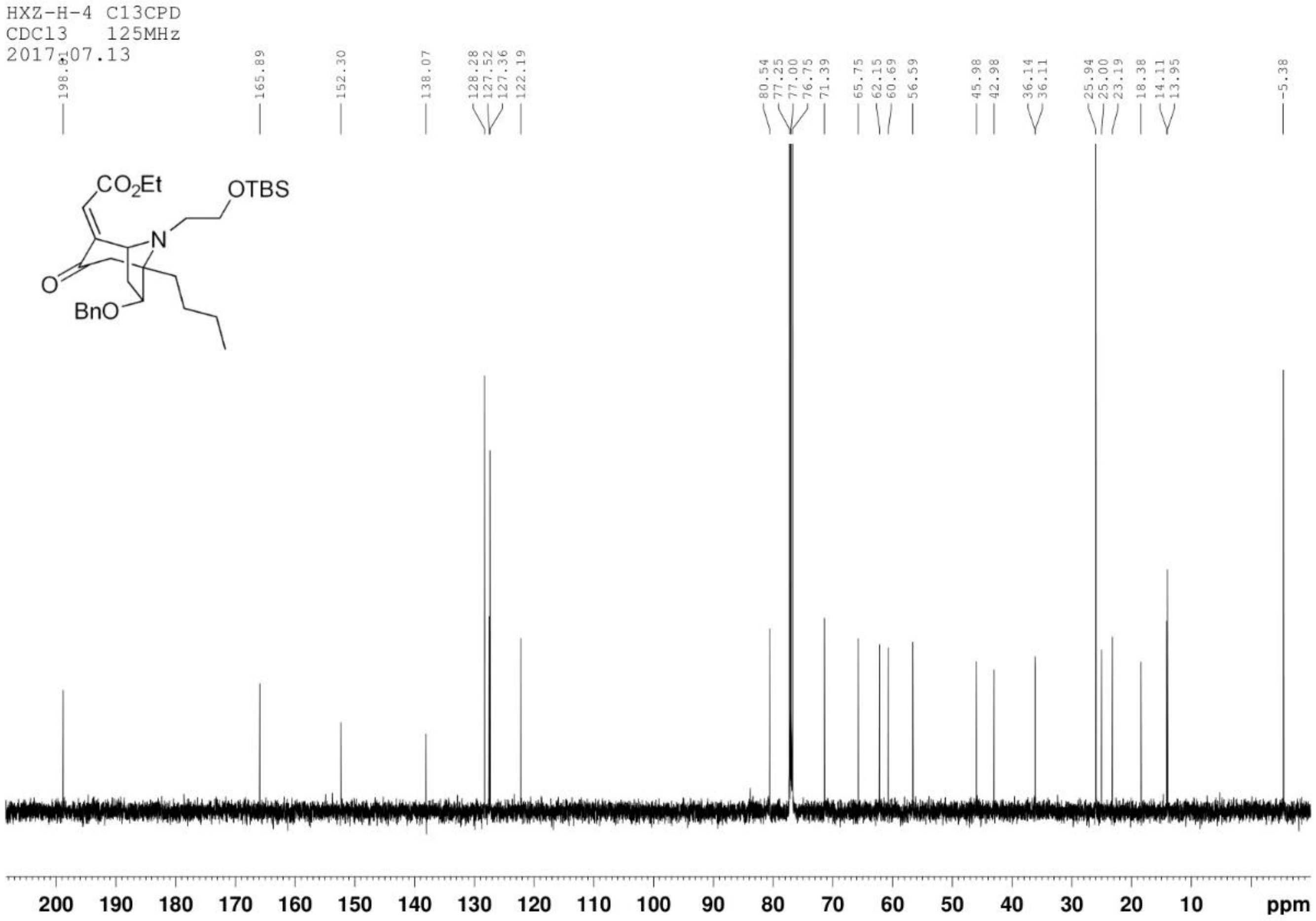

Supplementary Figure 39. ^{13}C NMR spectrum of compound **24**

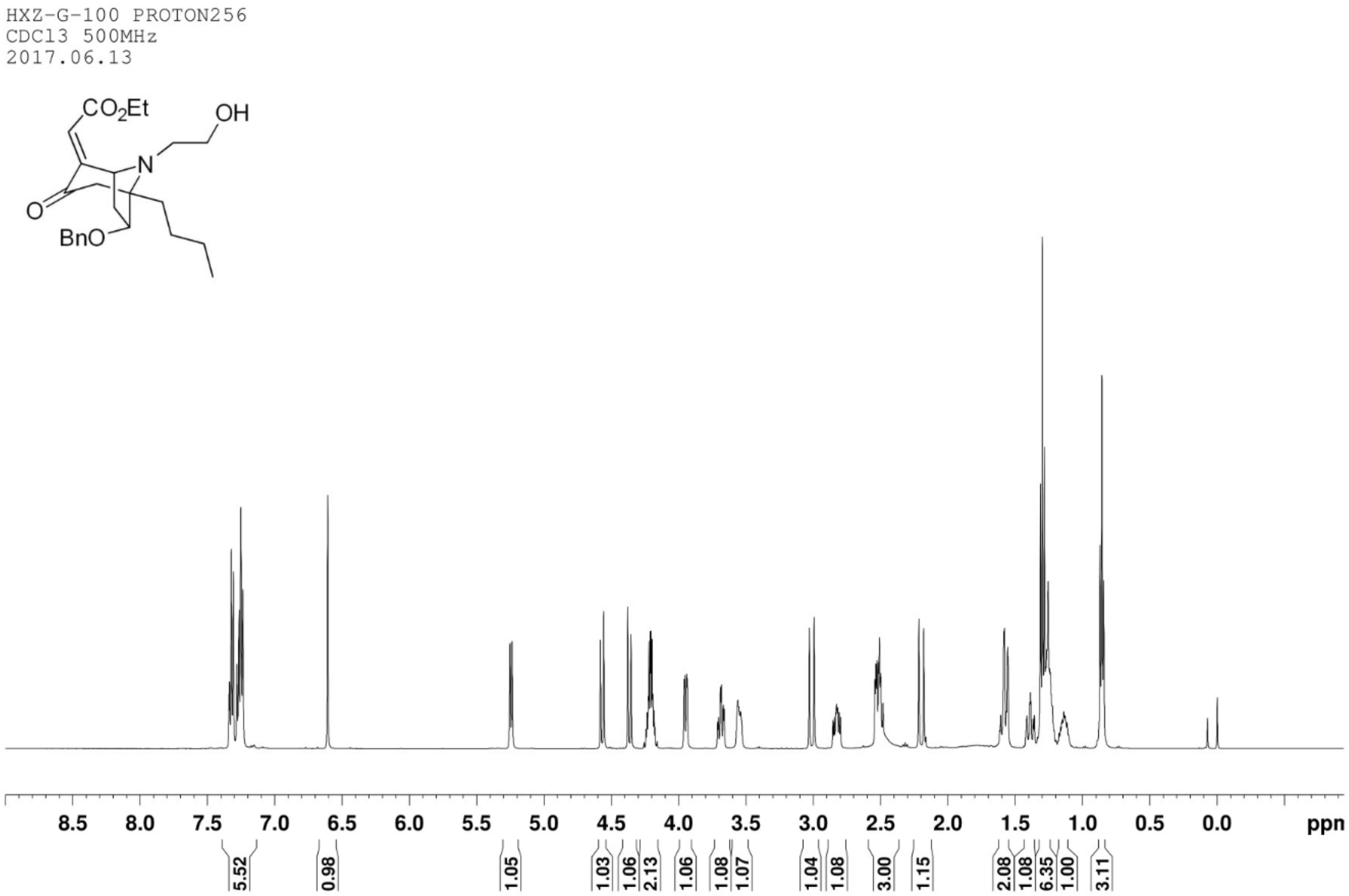

Supplementary Figure 40. ^{1}H NMR spectrum of compound **25**

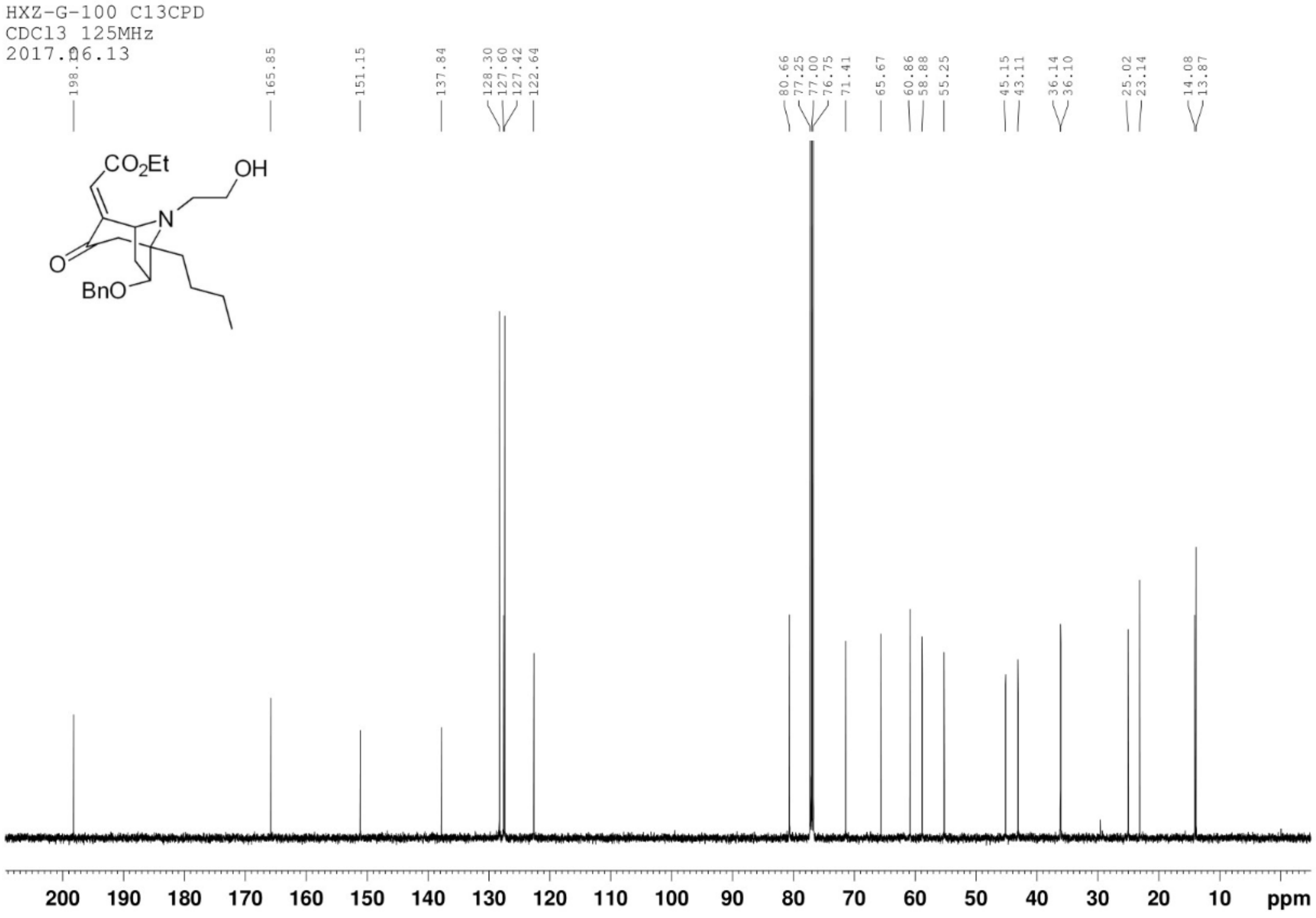

Supplementary Figure 41. ^{13}C NMR spectrum of compound **25**

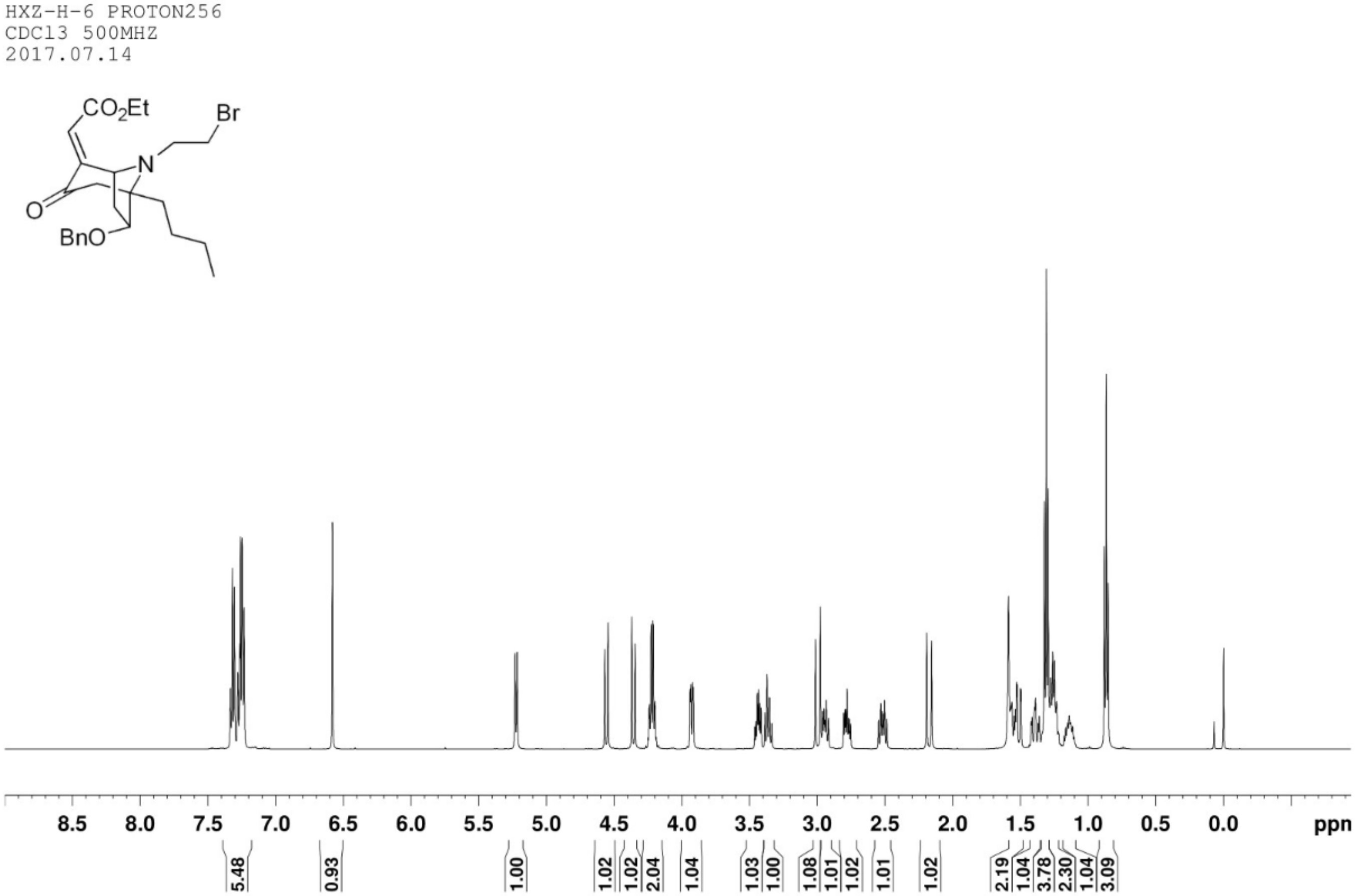

Supplementary Figure 42. ^{1}H NMR spectrum of compound **26**

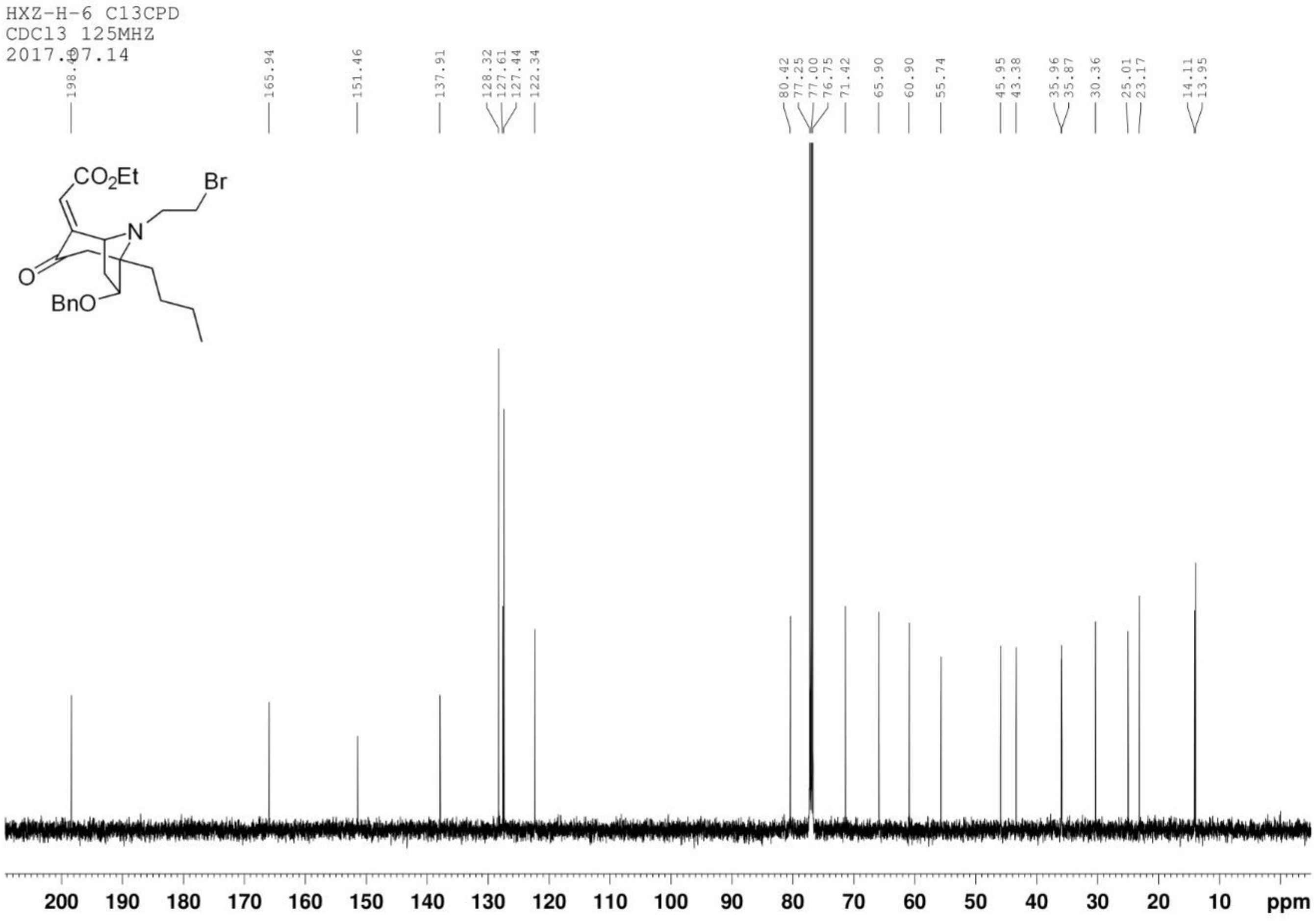

Supplementary Figure 43. ^{13}C NMR spectrum of compound **26**

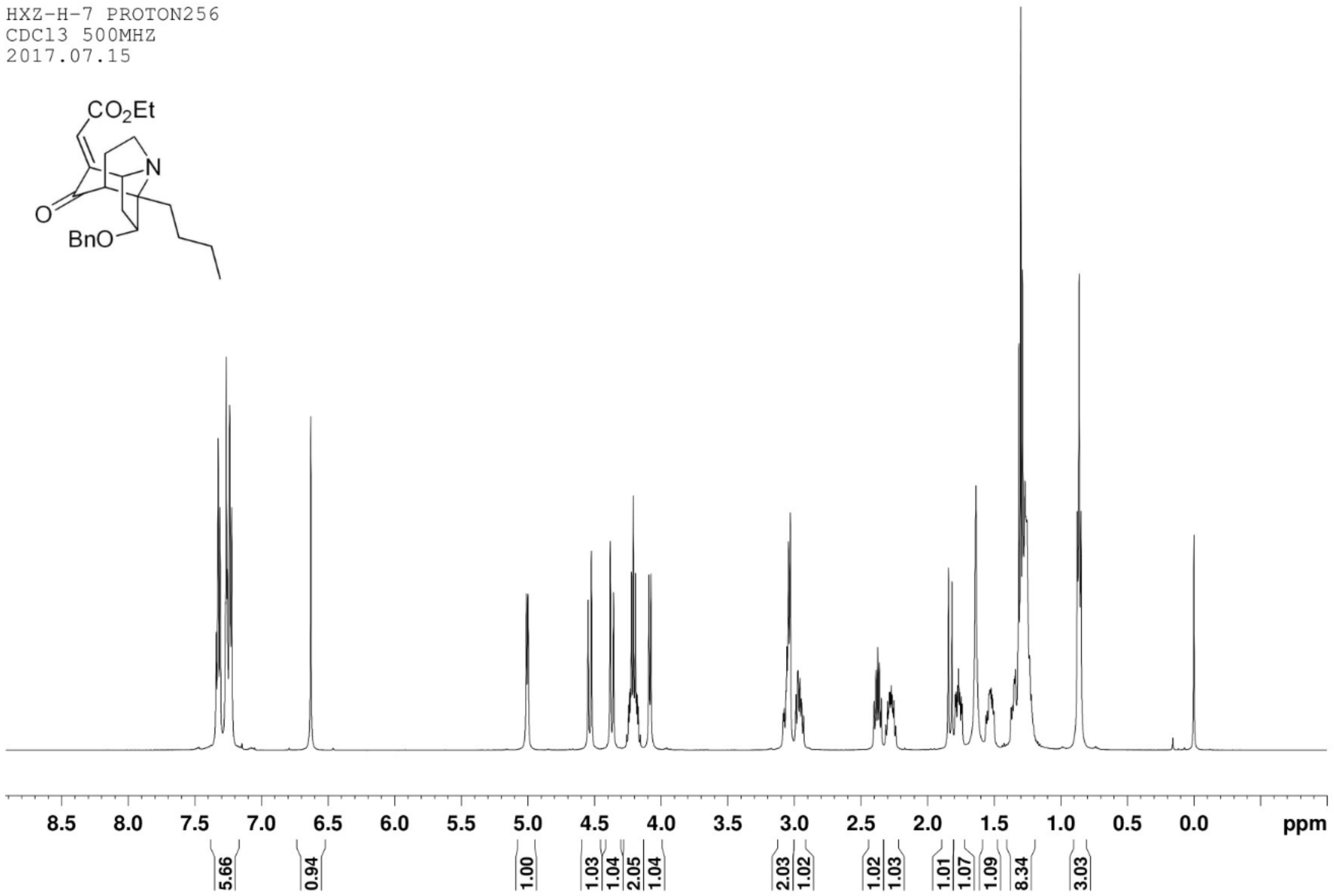

Supplementary Figure 44. ^{1}H NMR spectrum of compound **14**

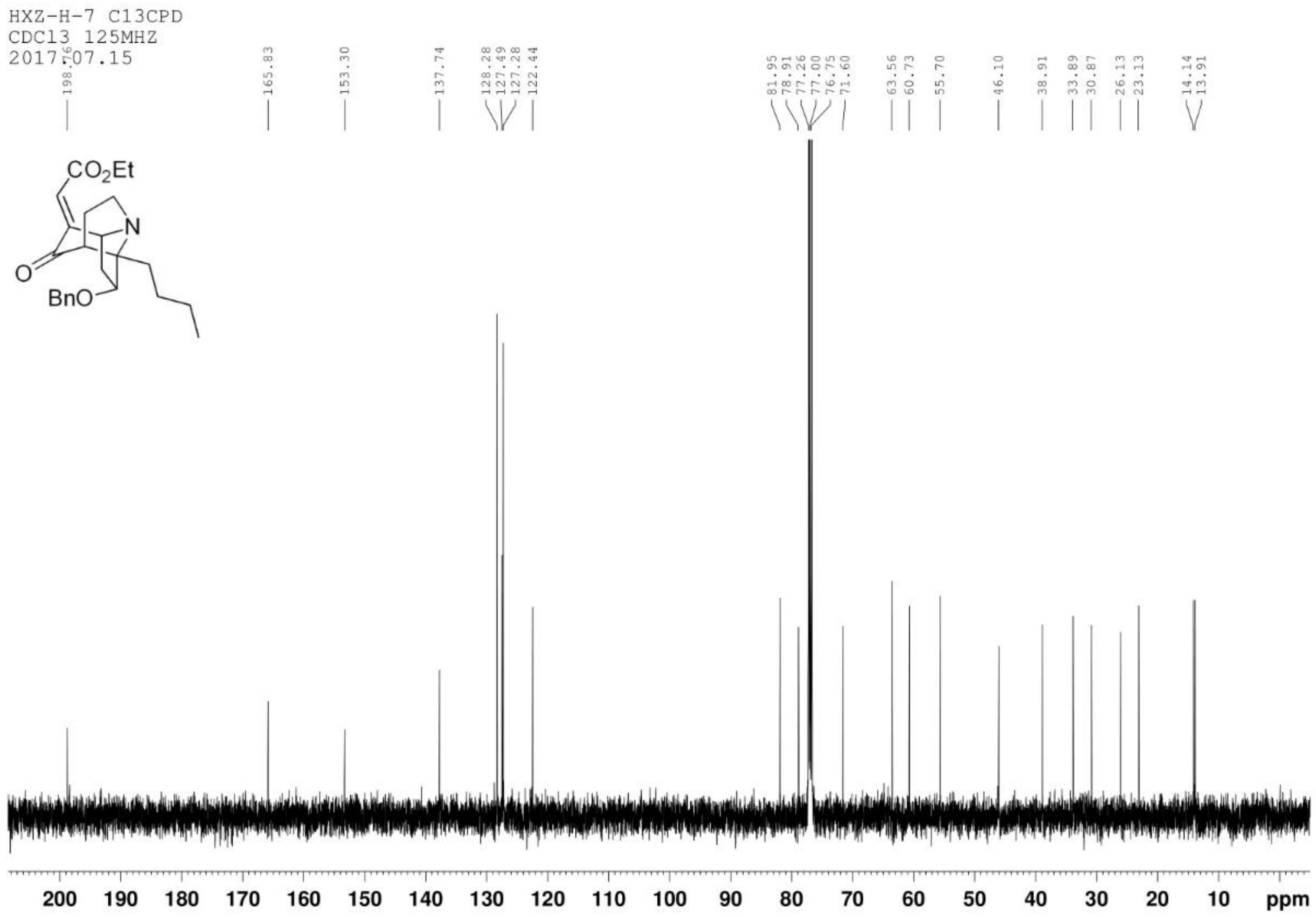

Supplementary Figure 45. ^{13}C NMR spectrum of compound **14**

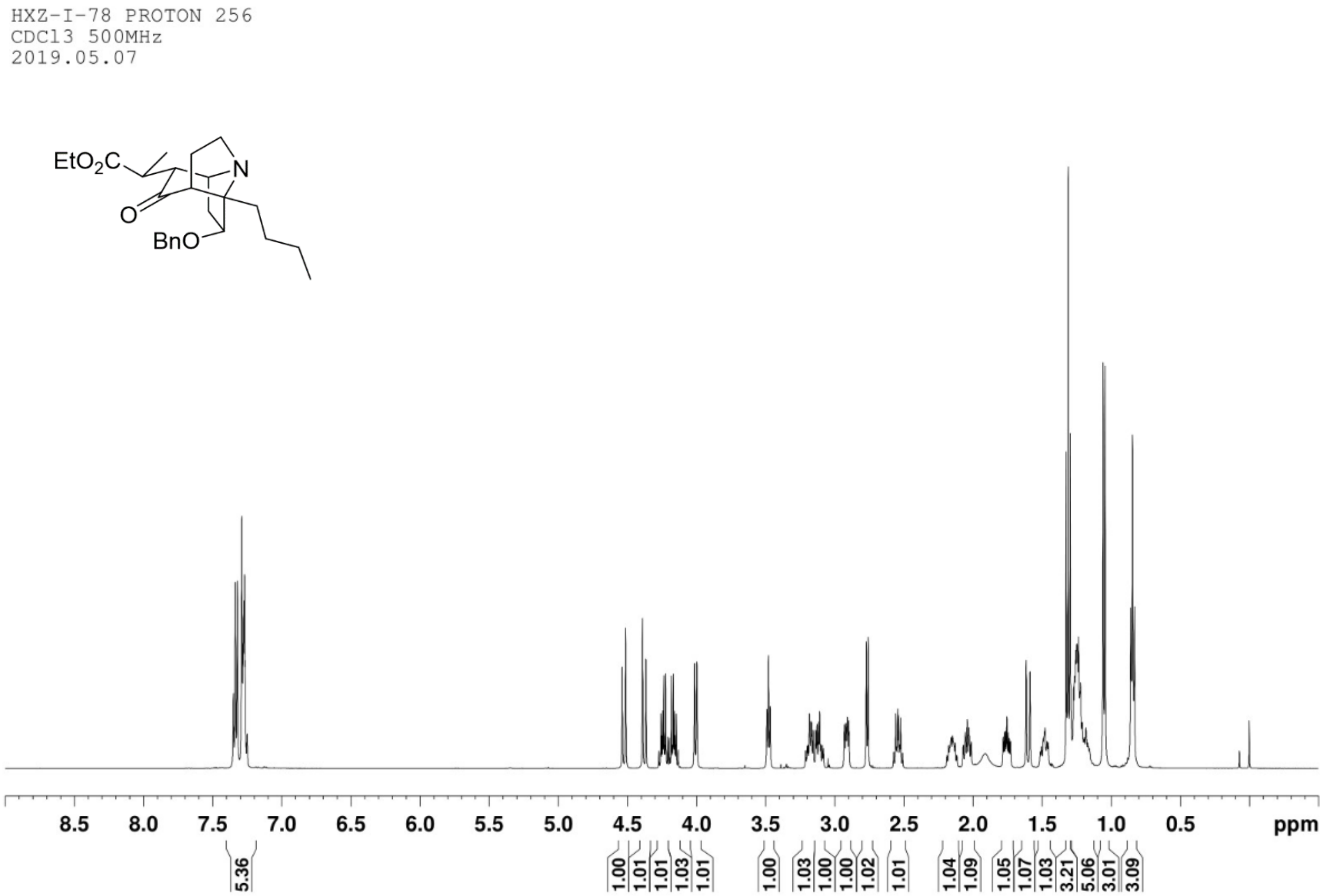

Supplementary Figure 46. ^{1}H NMR spectrum of compound **13**

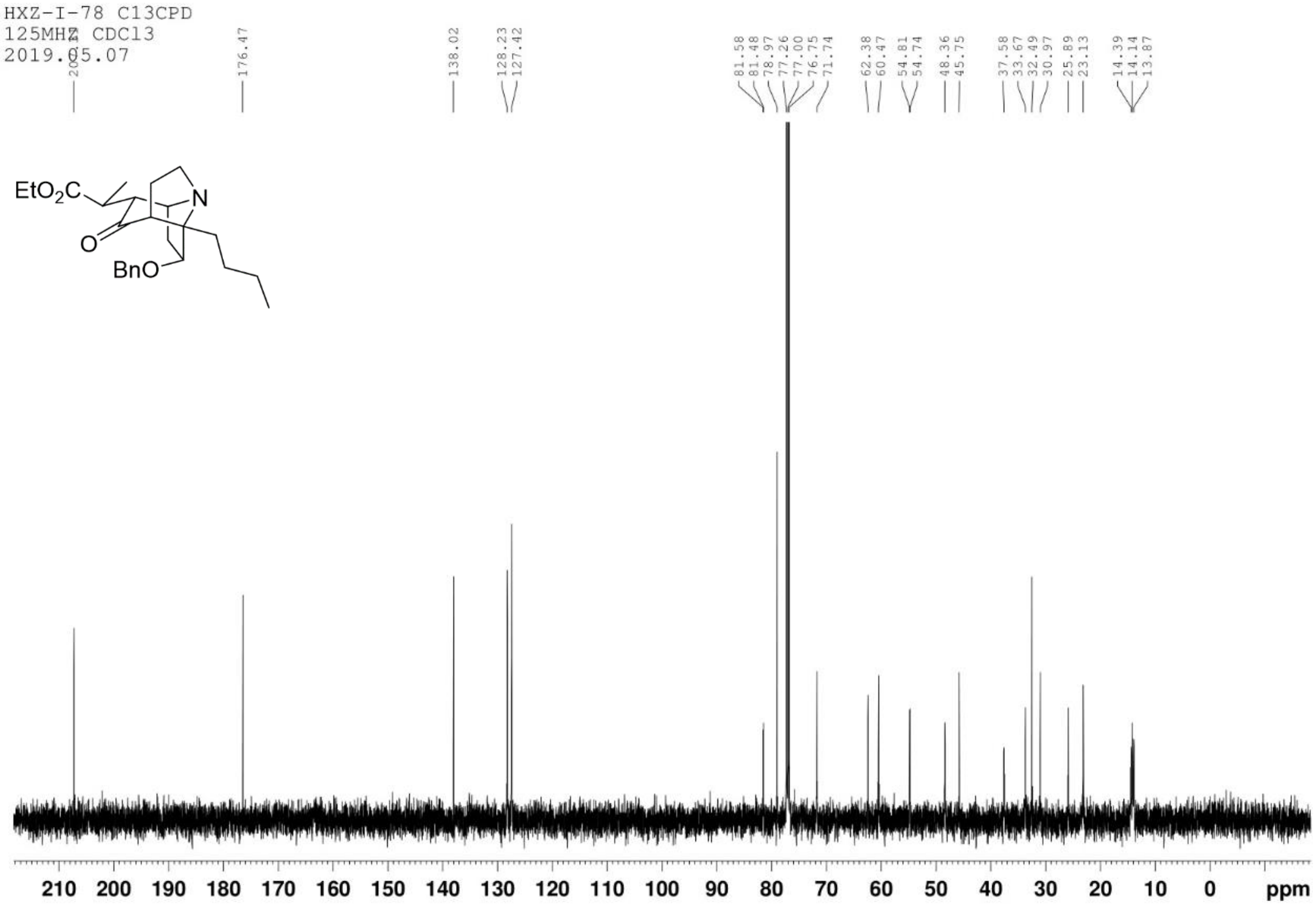

Supplementary Figure 47. ^{13}C NMR spectrum of compound **13**

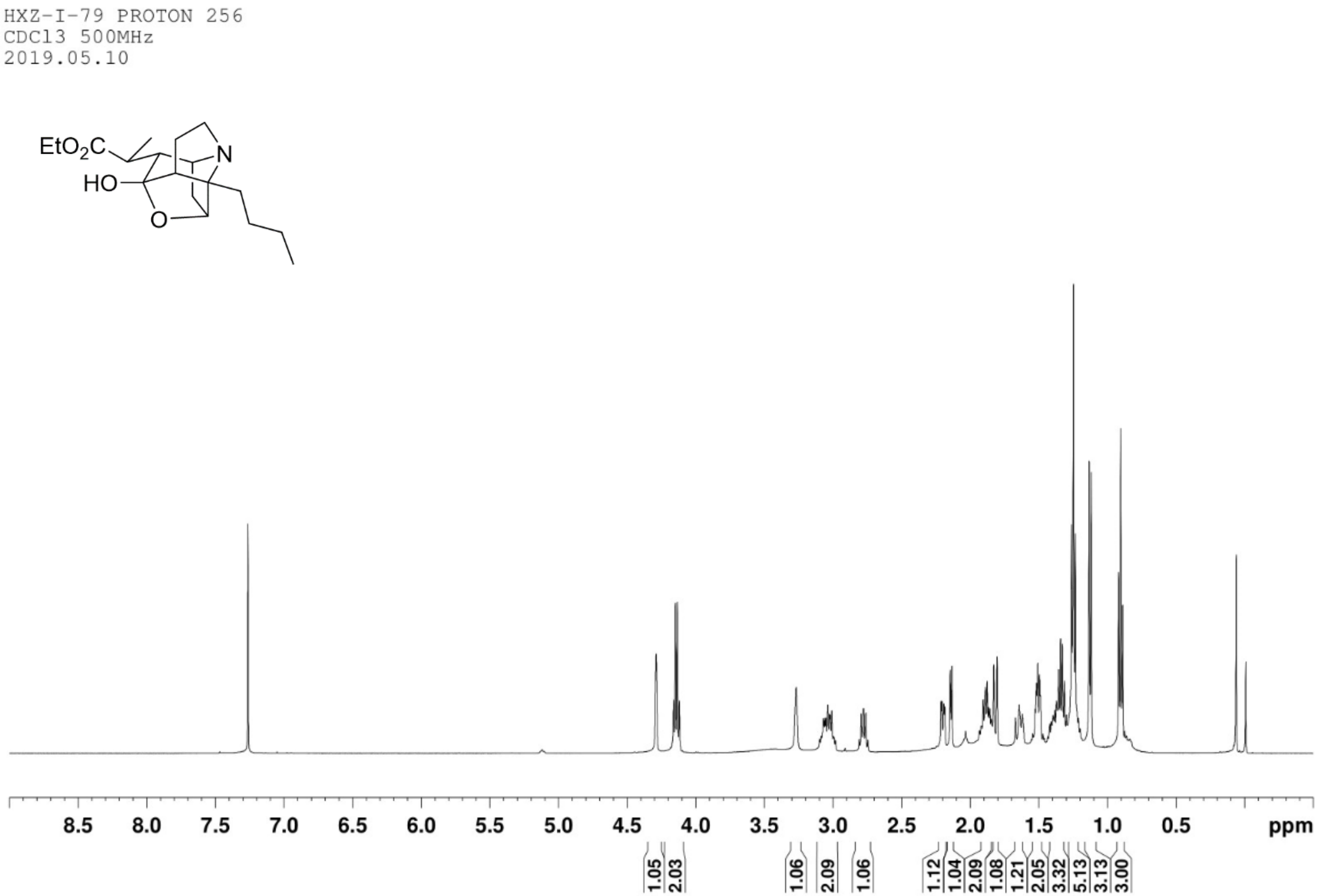

Supplementary Figure 48. ^{1}H NMR spectrum of compound **27**

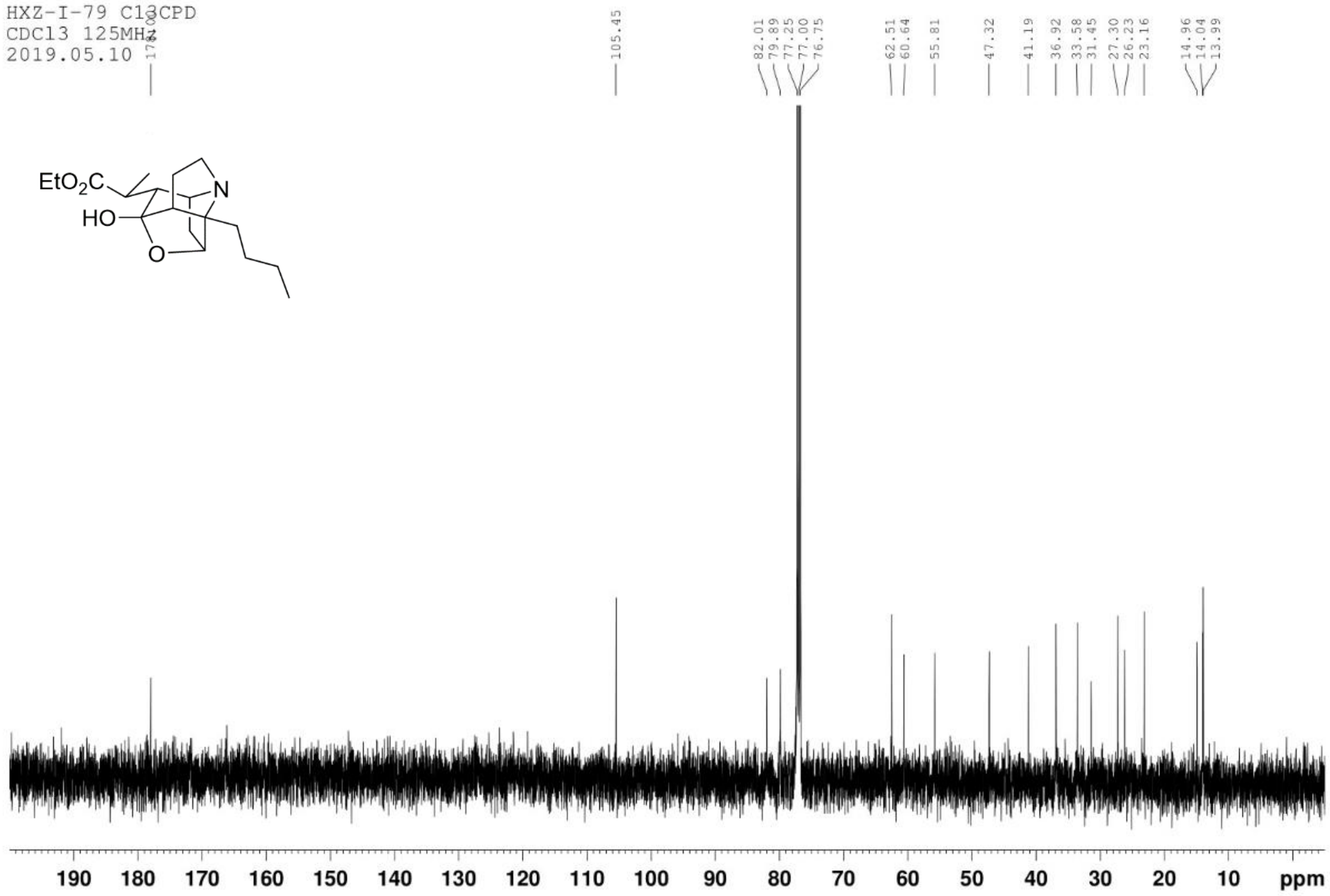

Supplementary Figure 49. ^{13}C NMR spectrum of compound **27**

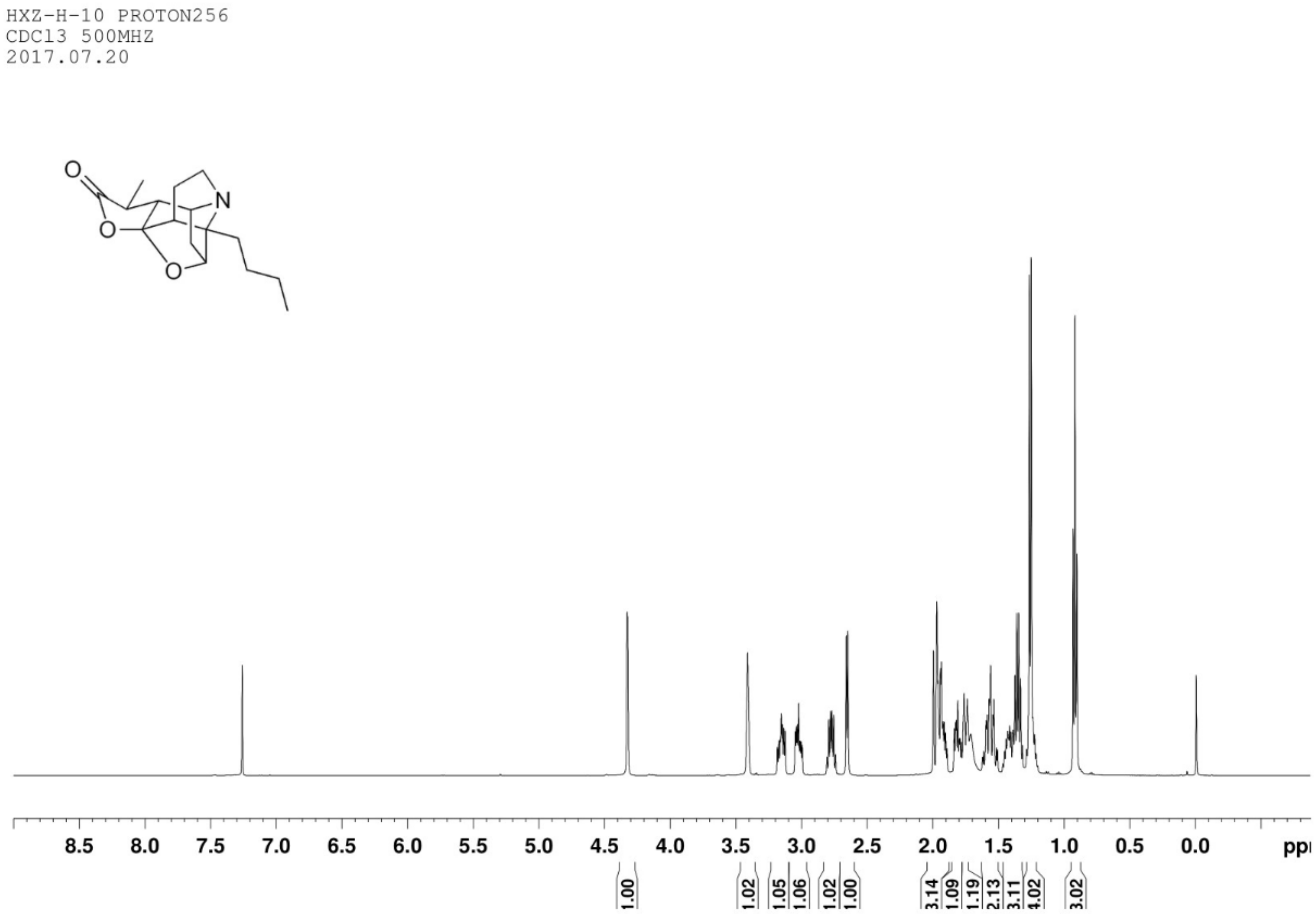

Supplementary Figure 50. ^{1}H NMR spectrum of compound **12**

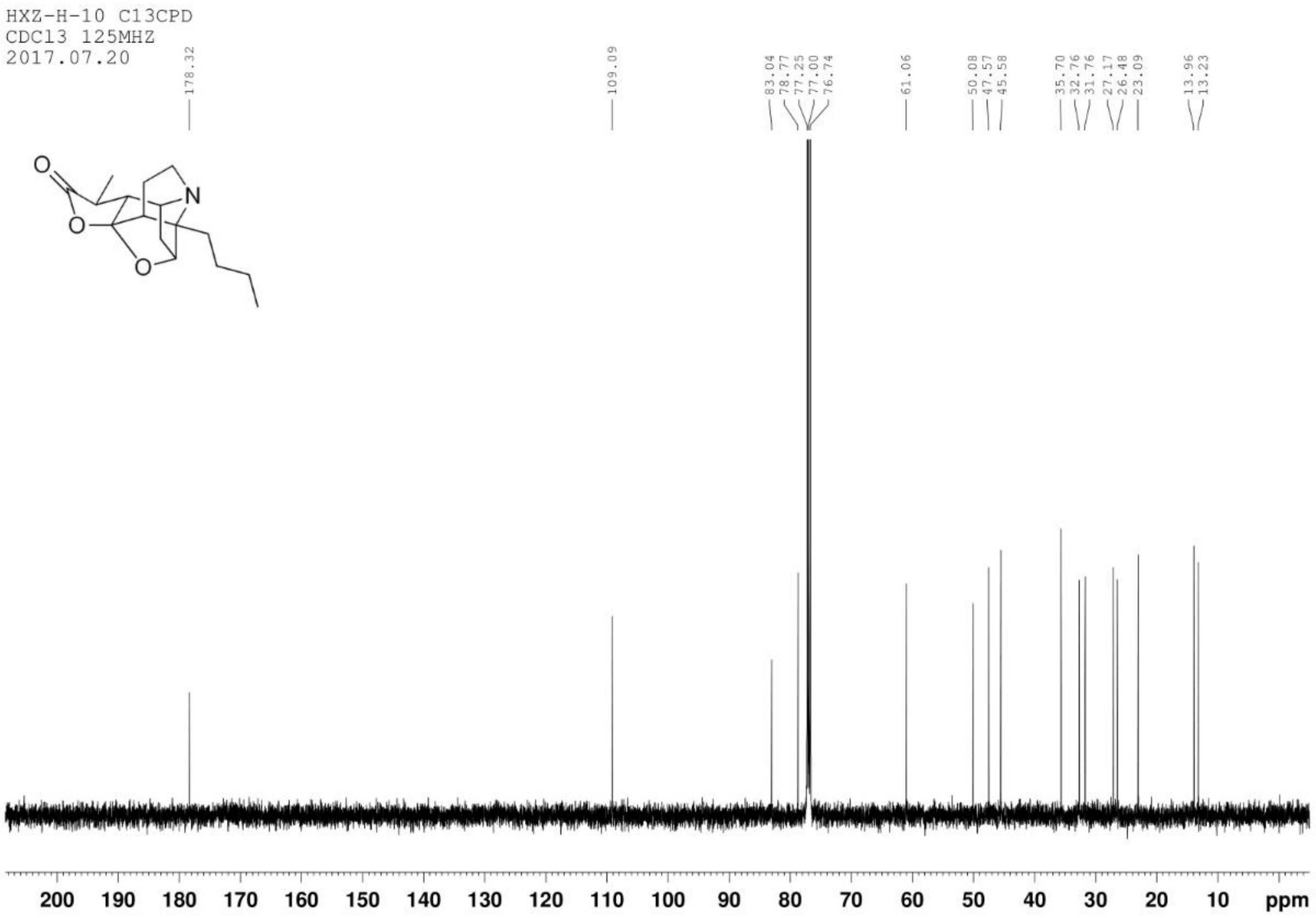

Supplementary Figure 51. ^{13}C NMR spectrum of compound **12**

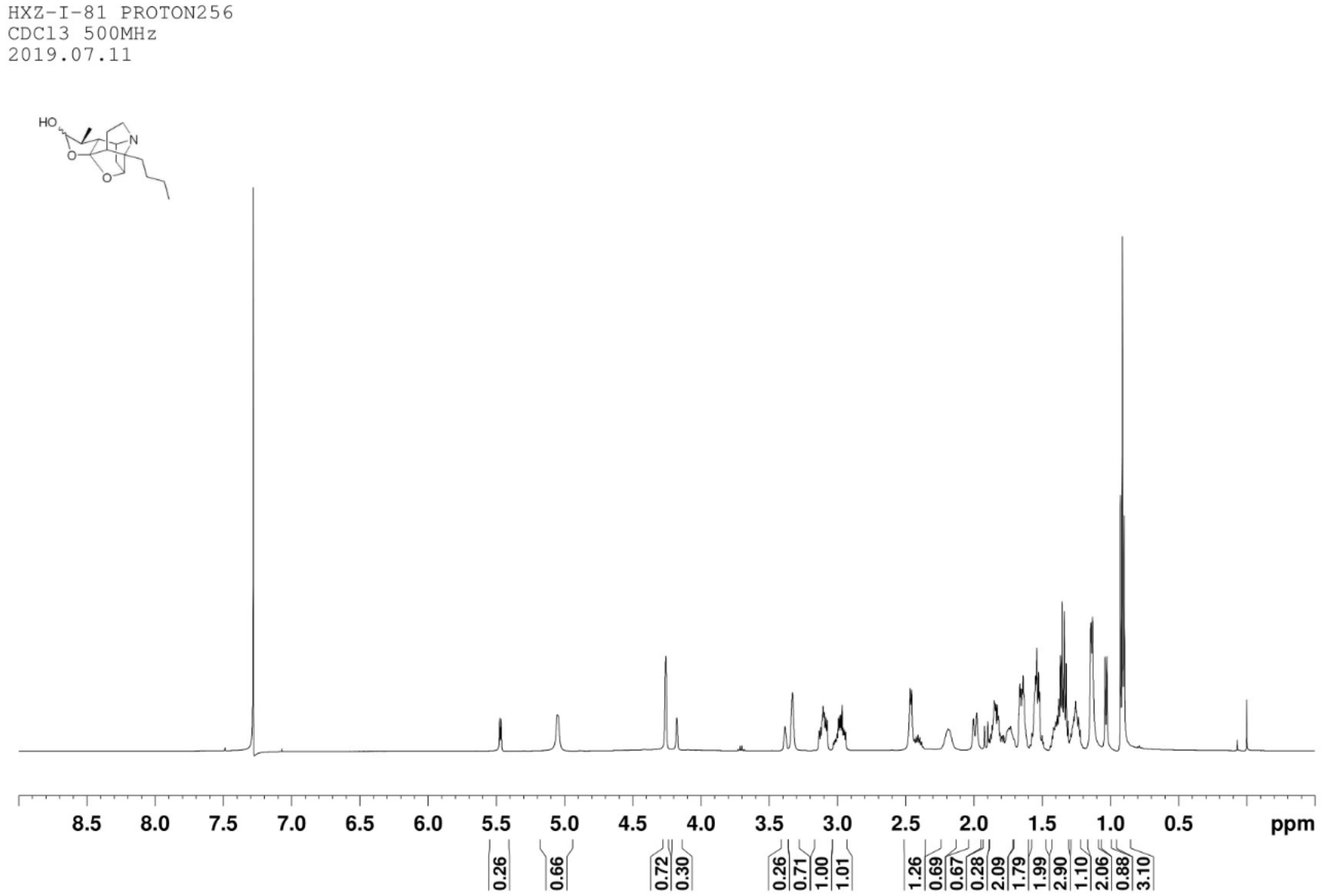

Supplementary Figure 52. ^{1}H NMR spectrum of compound **11**

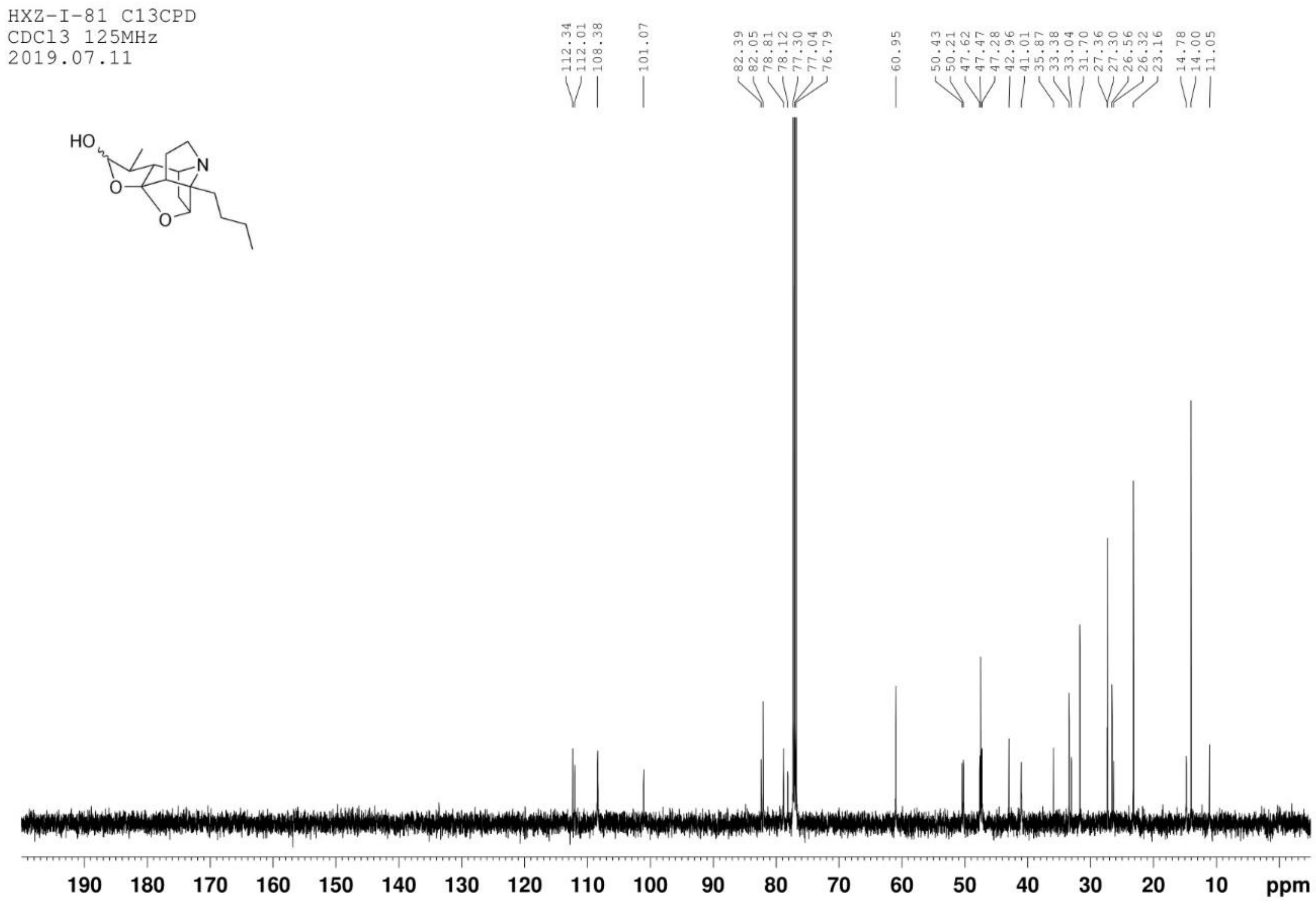

Supplementary Figure 53. ^{13}C NMR spectrum of compound **11**

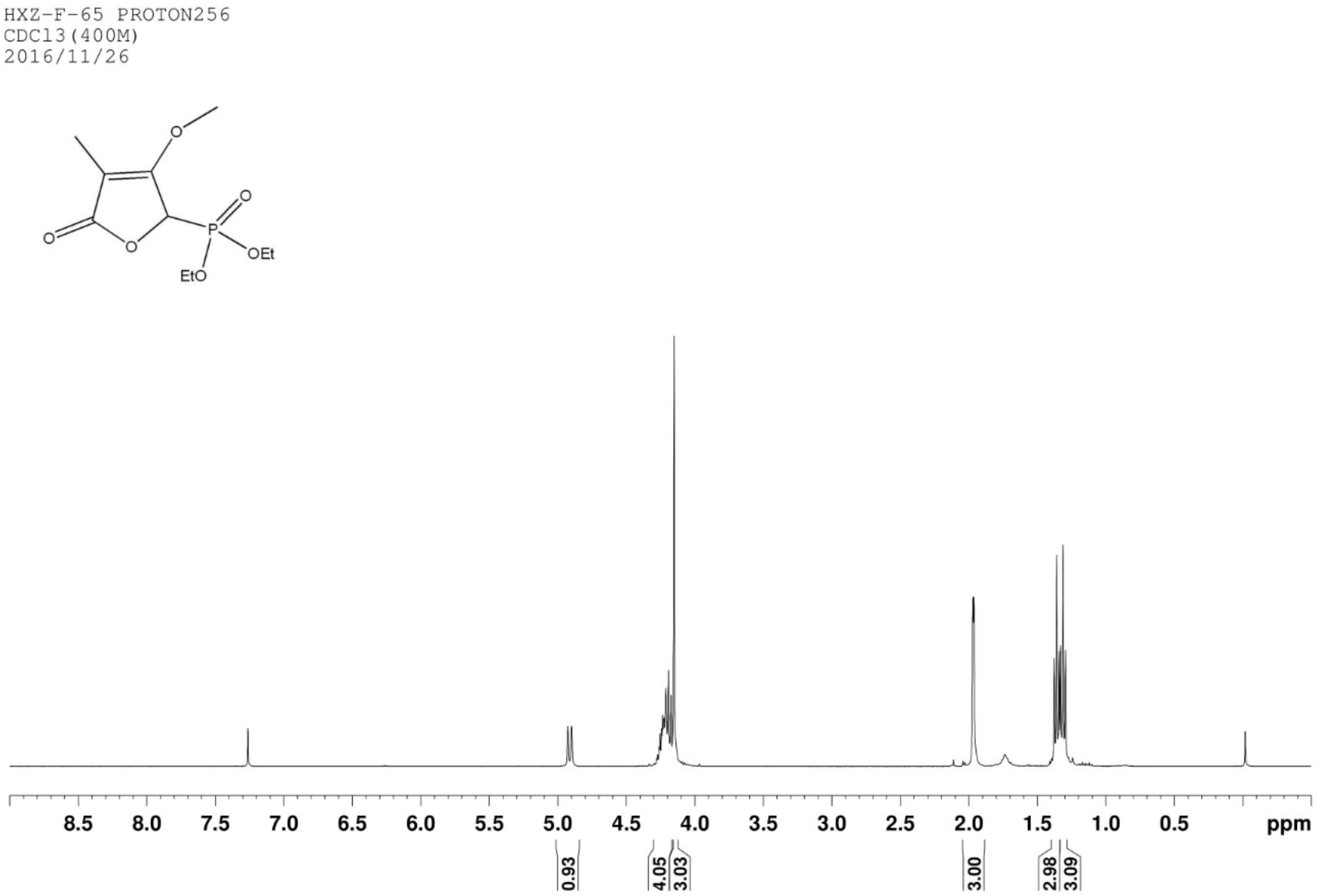

Supplementary Figure 54. ^{1}H NMR spectrum of compound **10**

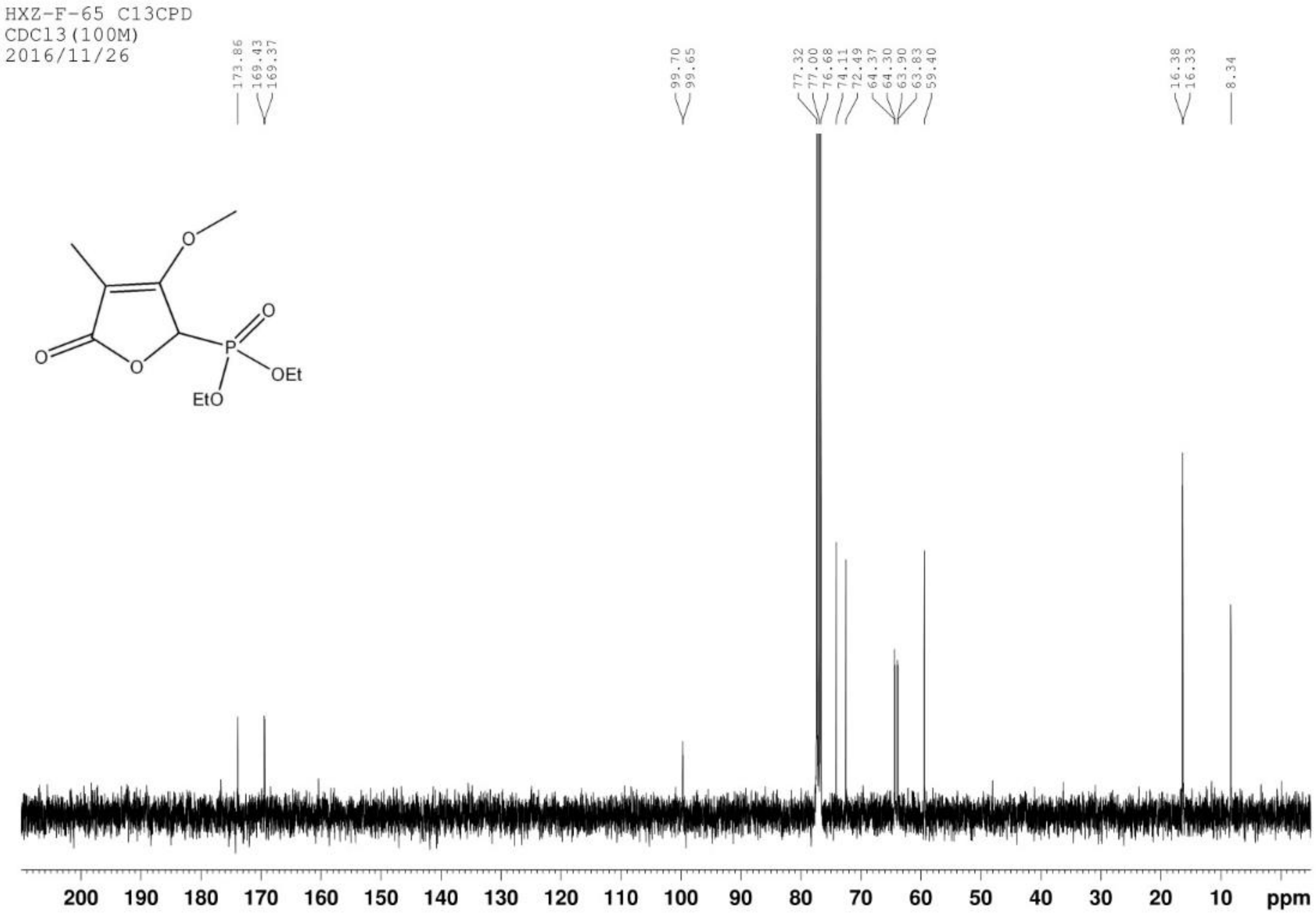

Supplementary Figure 55. ^{13}C NMR spectrum of compound **10**

HXZ-H-12a2 PROTON256
500 MHZ CDCl3
2017.07.27

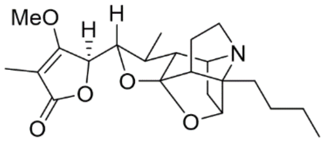

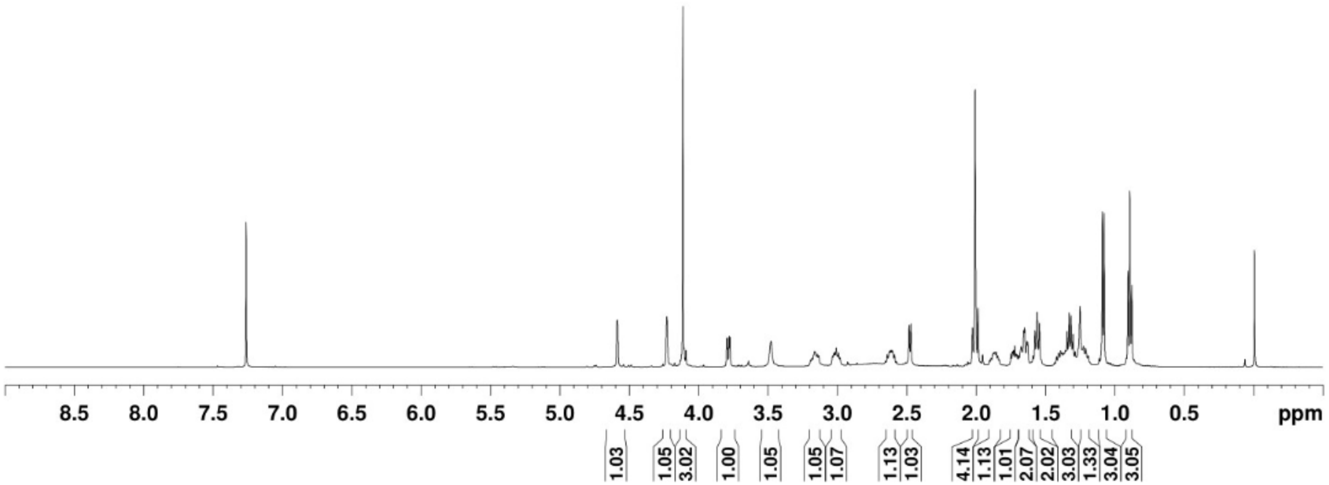

Supplementary Figure 56. ^{1}H NMR spectrum of compound (11*S*,12*R*)-dihydrostemofoline (**8**)

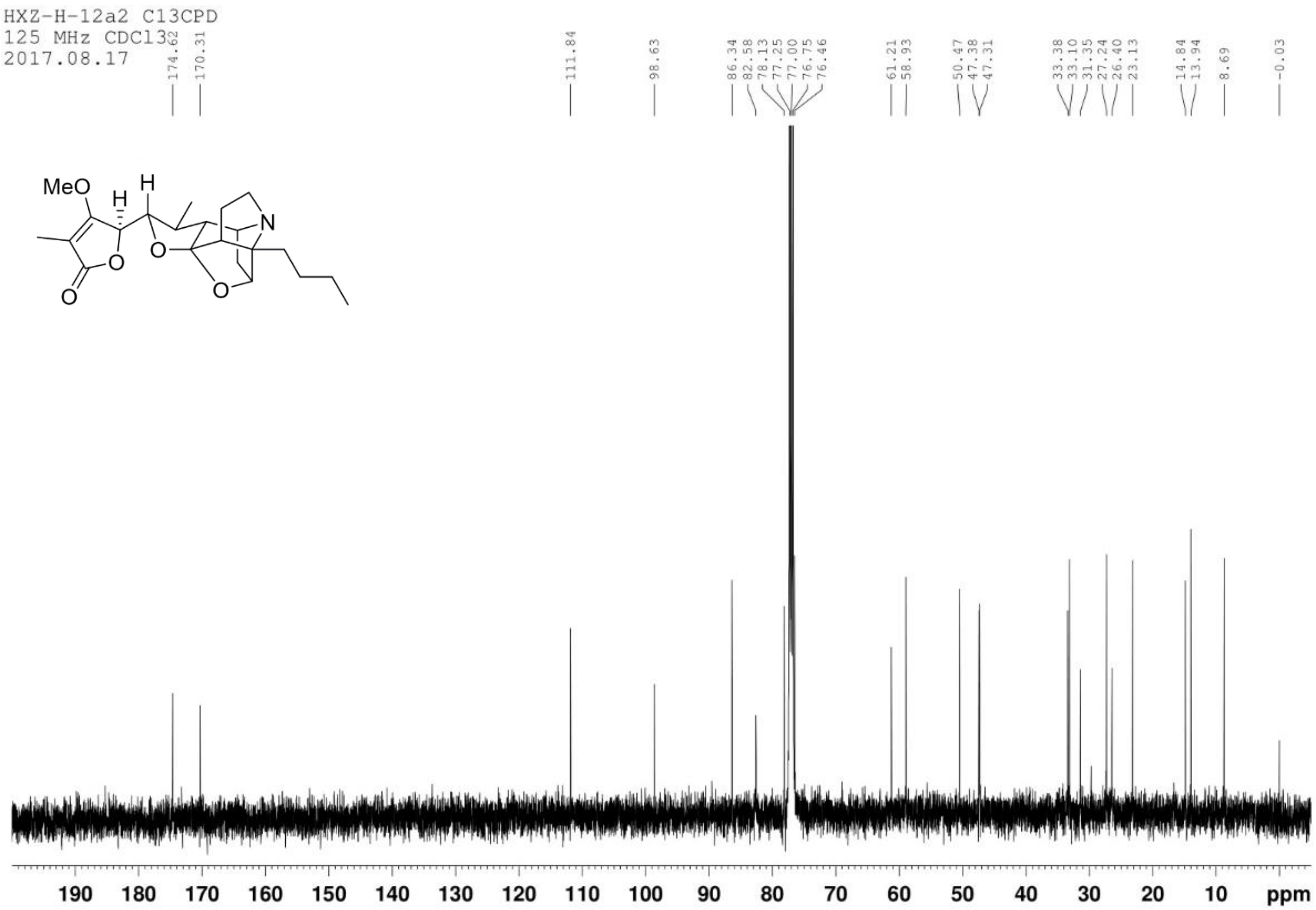

Supplementary Figure 57. ^{13}C NMR spectrum of compound (11*S*,12*R*)-dihydrostemofoline (**8**)

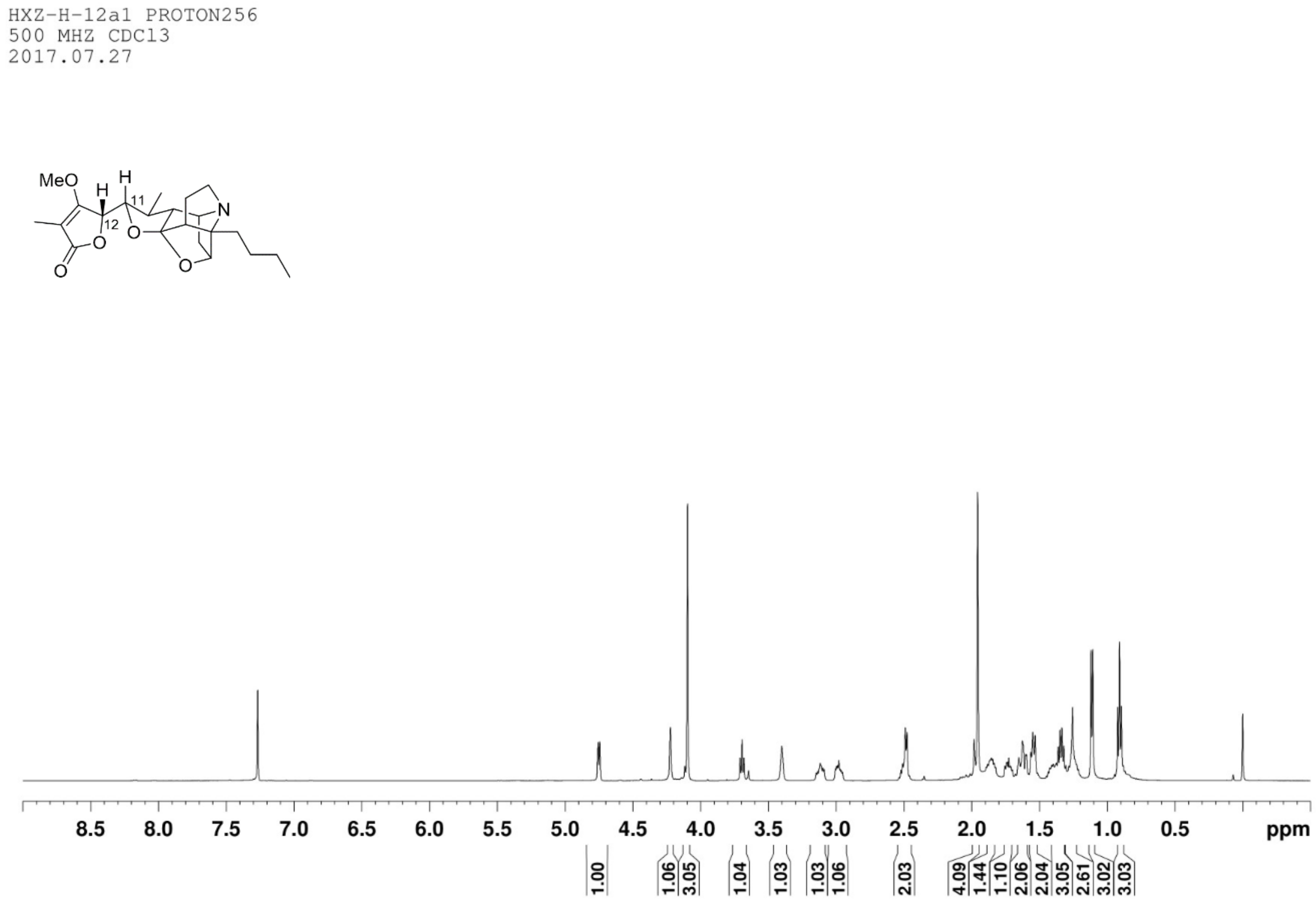

Supplementary Figure 58. ^{1}H NMR spectrum of compound (11*S*,12*S*)-dihydrostemofoline (**9**)

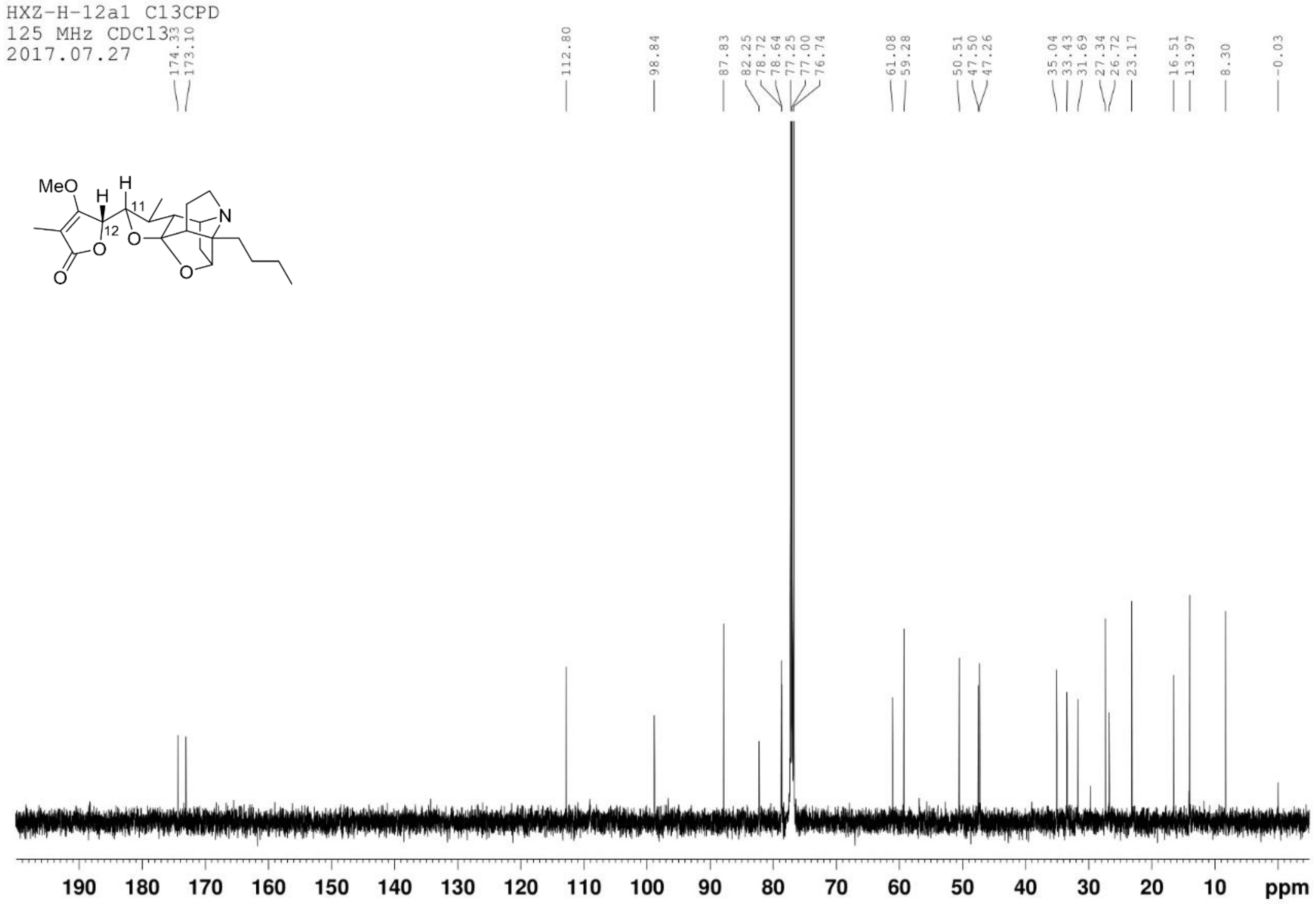

Supplementary Figure 59. ^{13}C NMR spectrum of compound (11*S*,12*S*)-dihydrostemofoline (**9**)

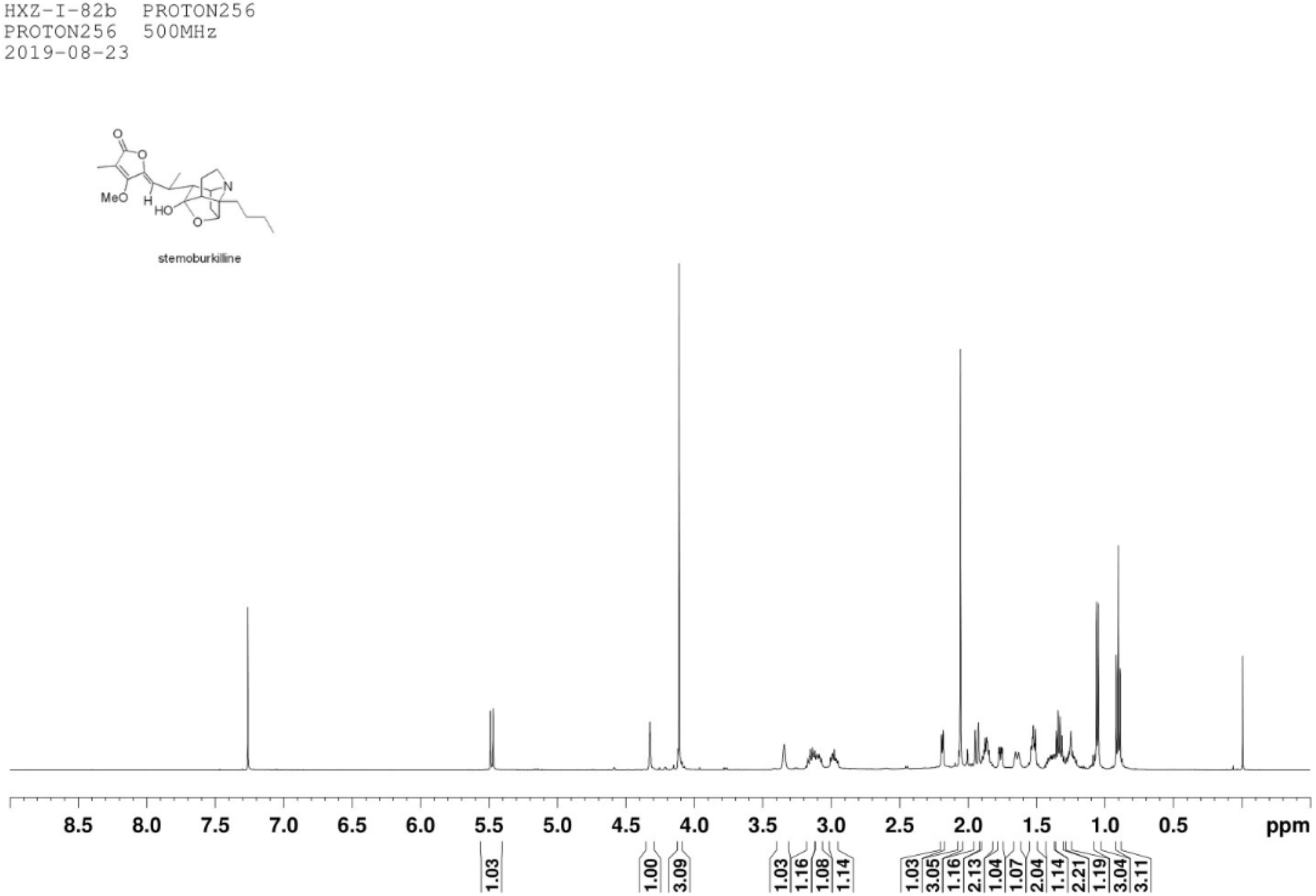

Supplementary Figure 60. ^{1}H NMR spectrum of compound stemoburkilline (**7**)

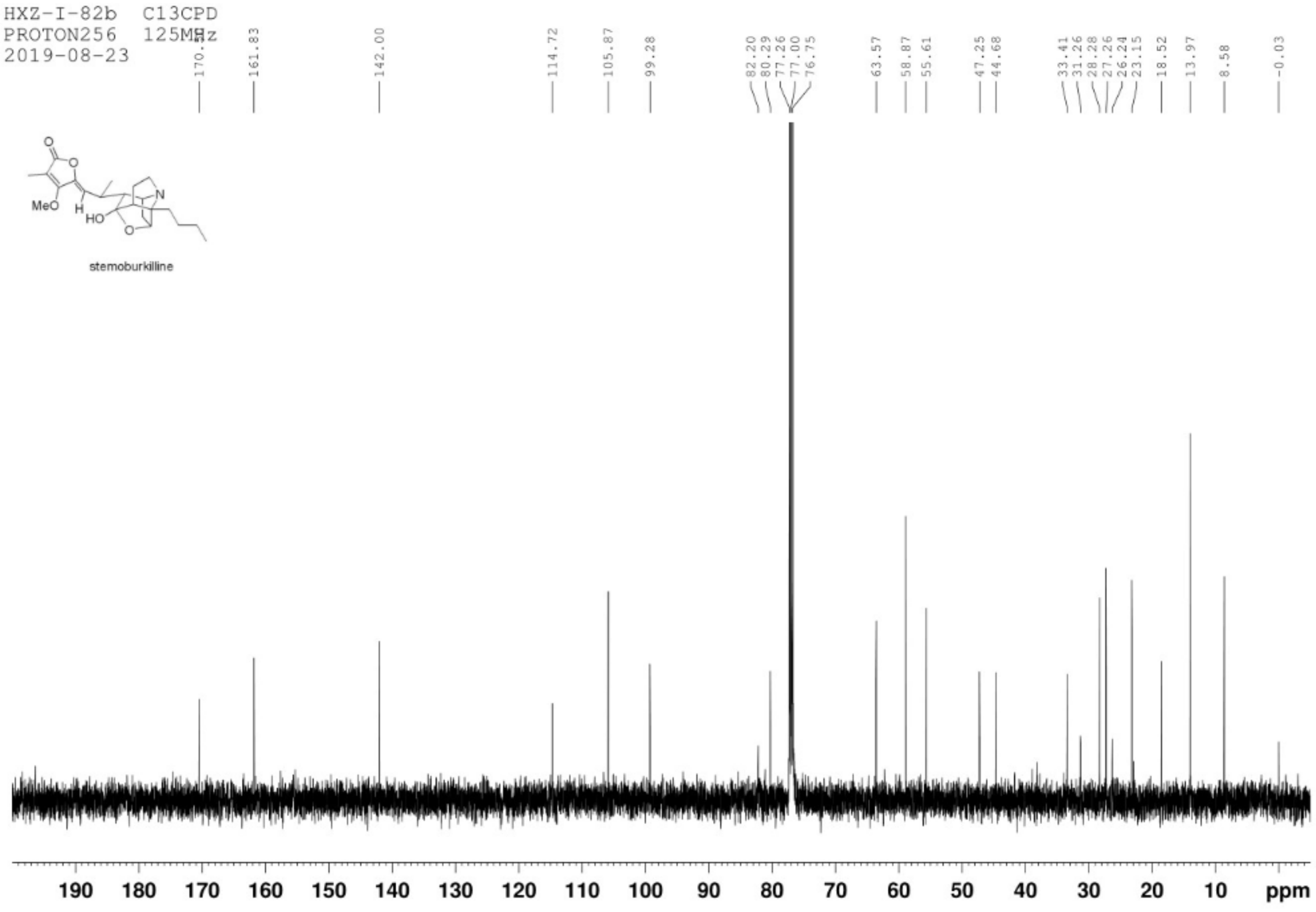

Supplementary Figure 61. ^{13}C NMR spectrum of compound stemoburkilline (**7**)

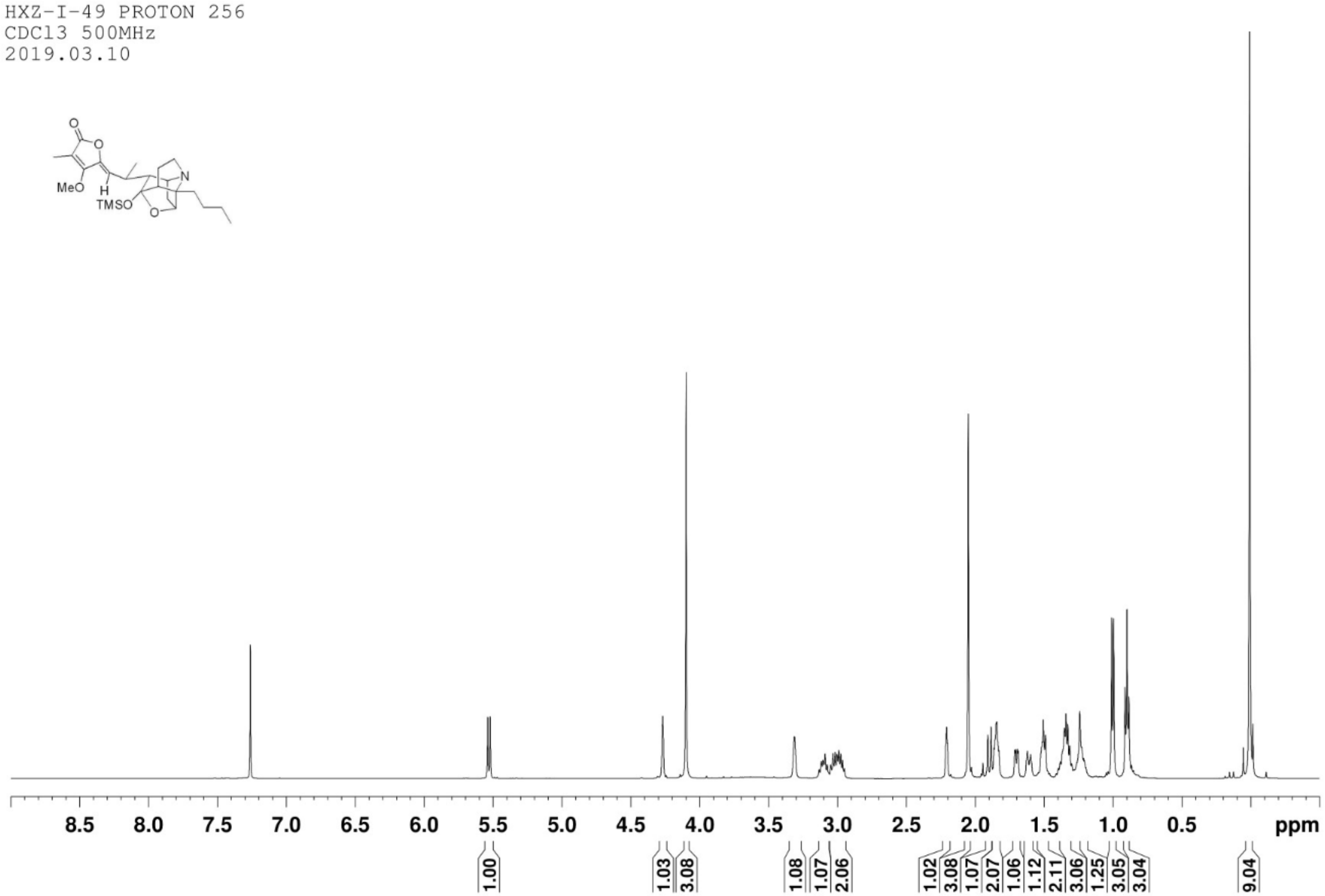

Supplementary Figure 62. ^{1}H NMR spectrum of compound **30**

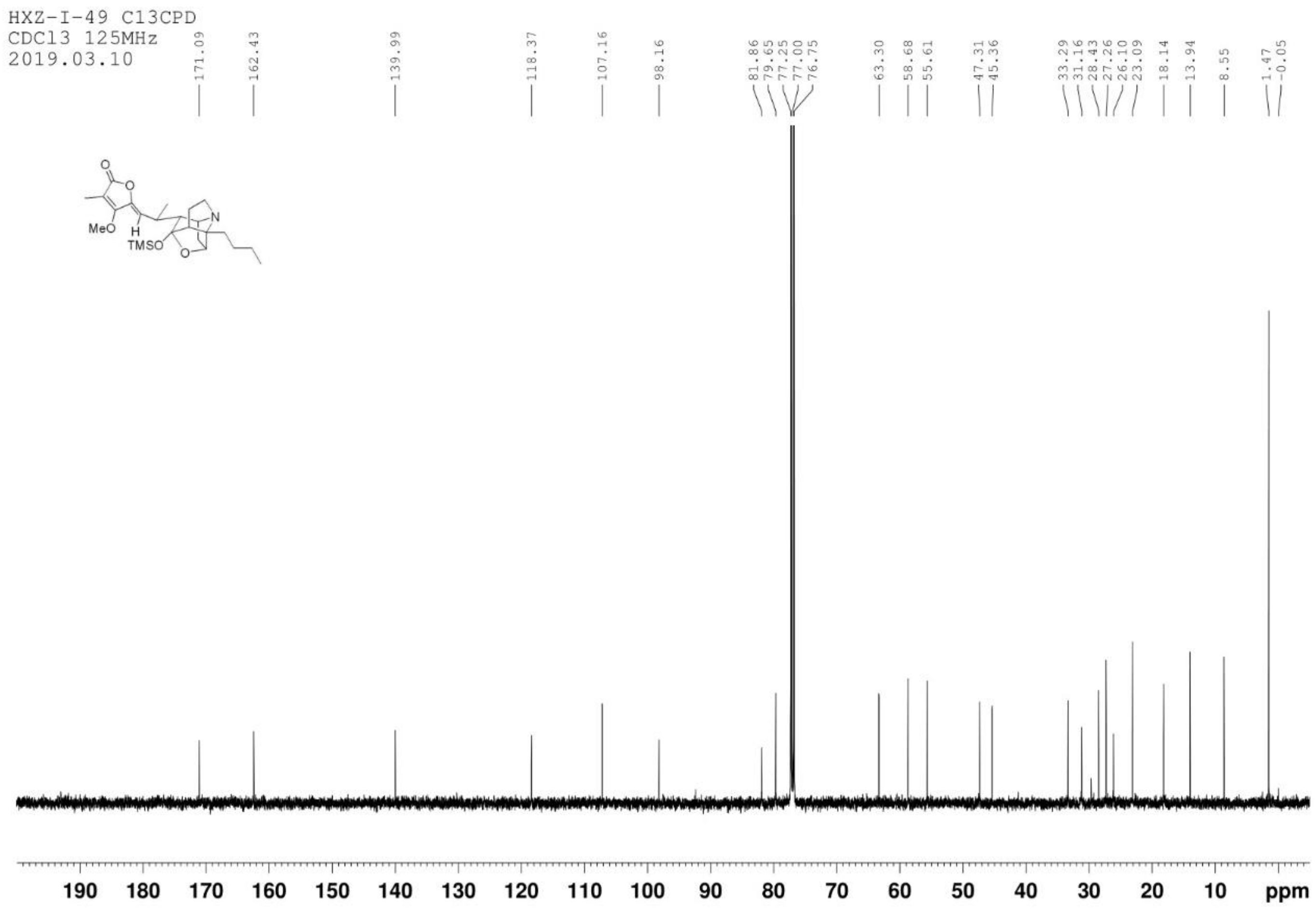

Supplementary Figure 63. ^{13}C NMR spectrum of compound **30**

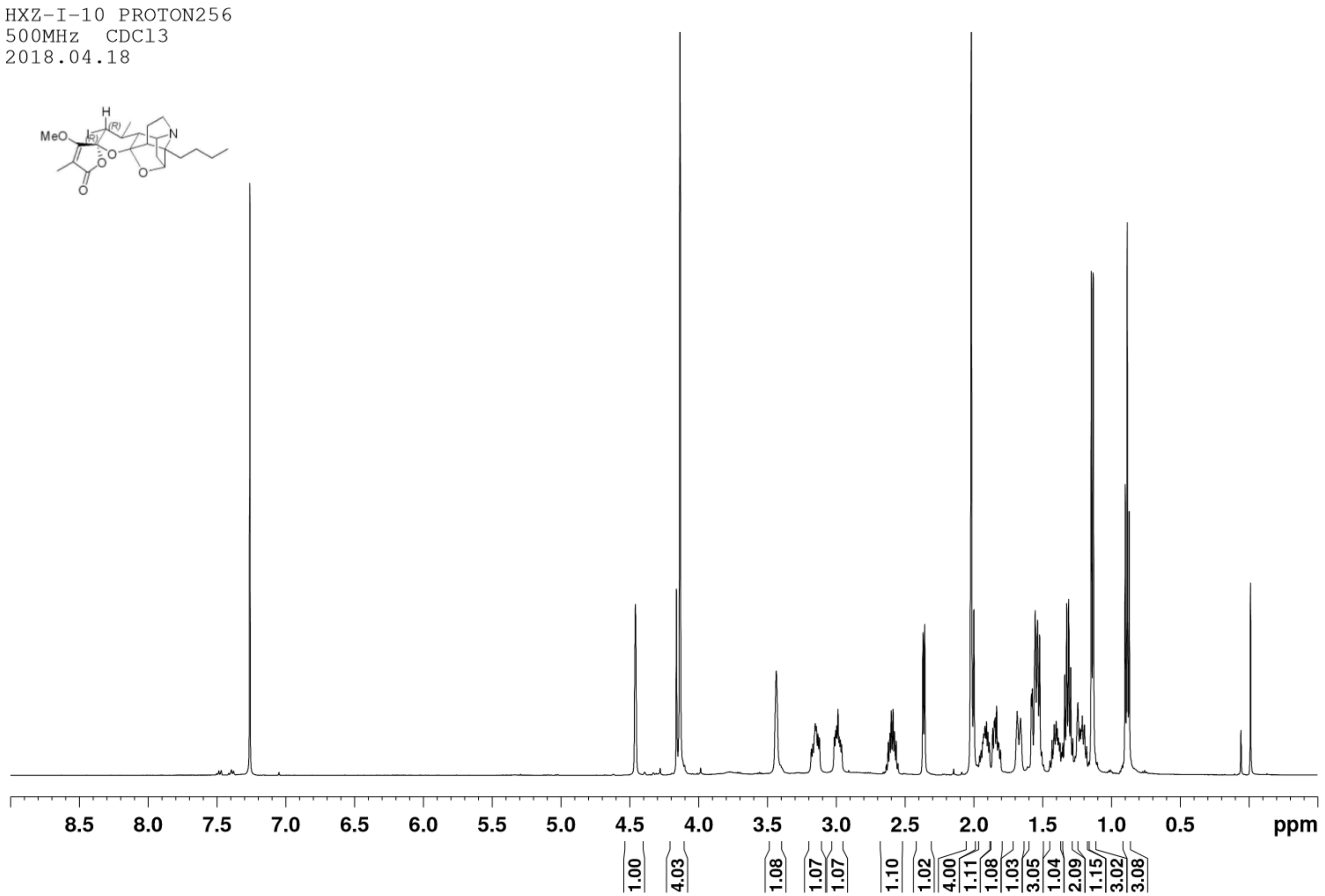

Supplementary Figure 64. ^{1}H NMR spectrum of compound **31**

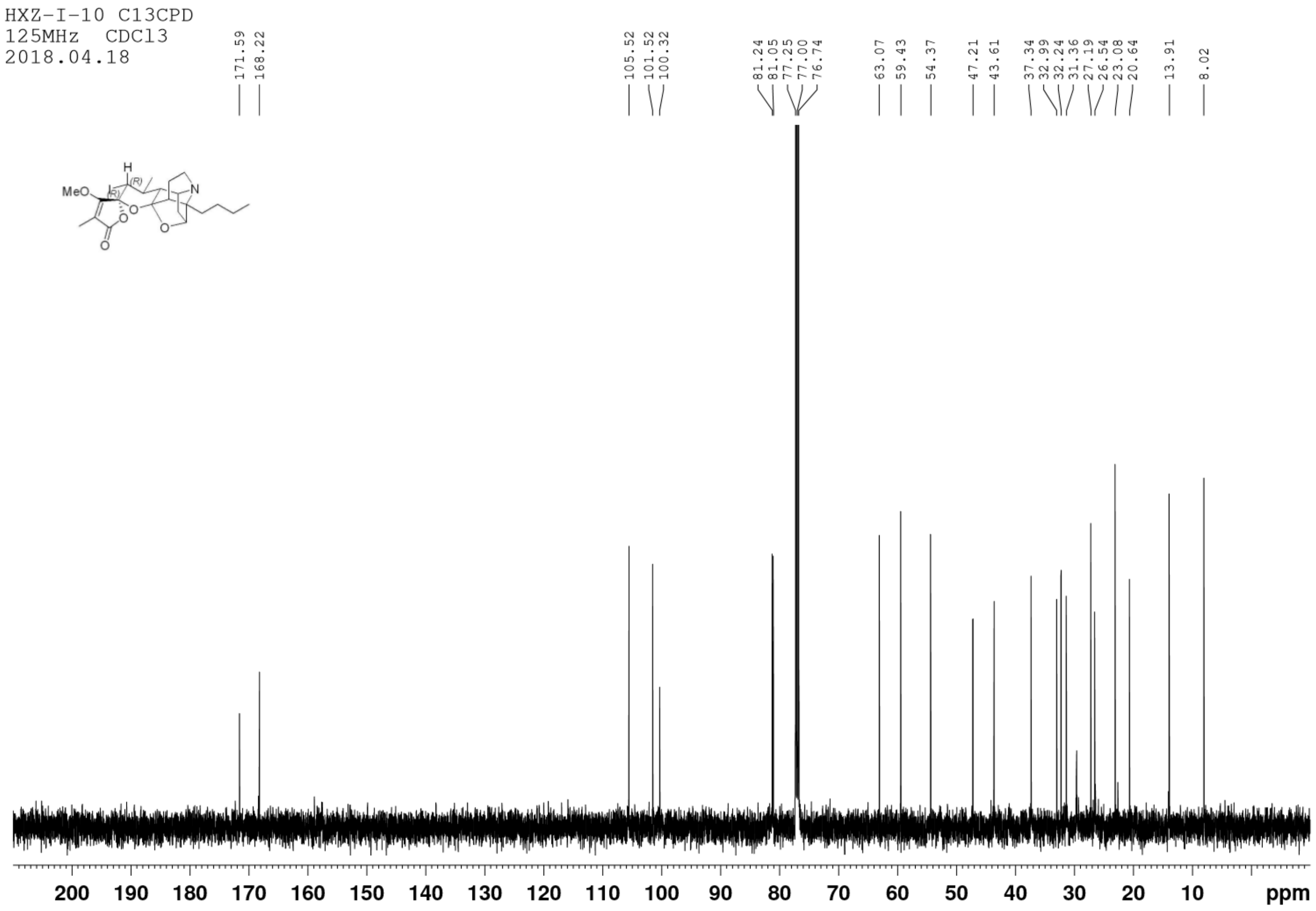

Supplementary Figure 65. ^{13}C NMR spectrum of compound **31**

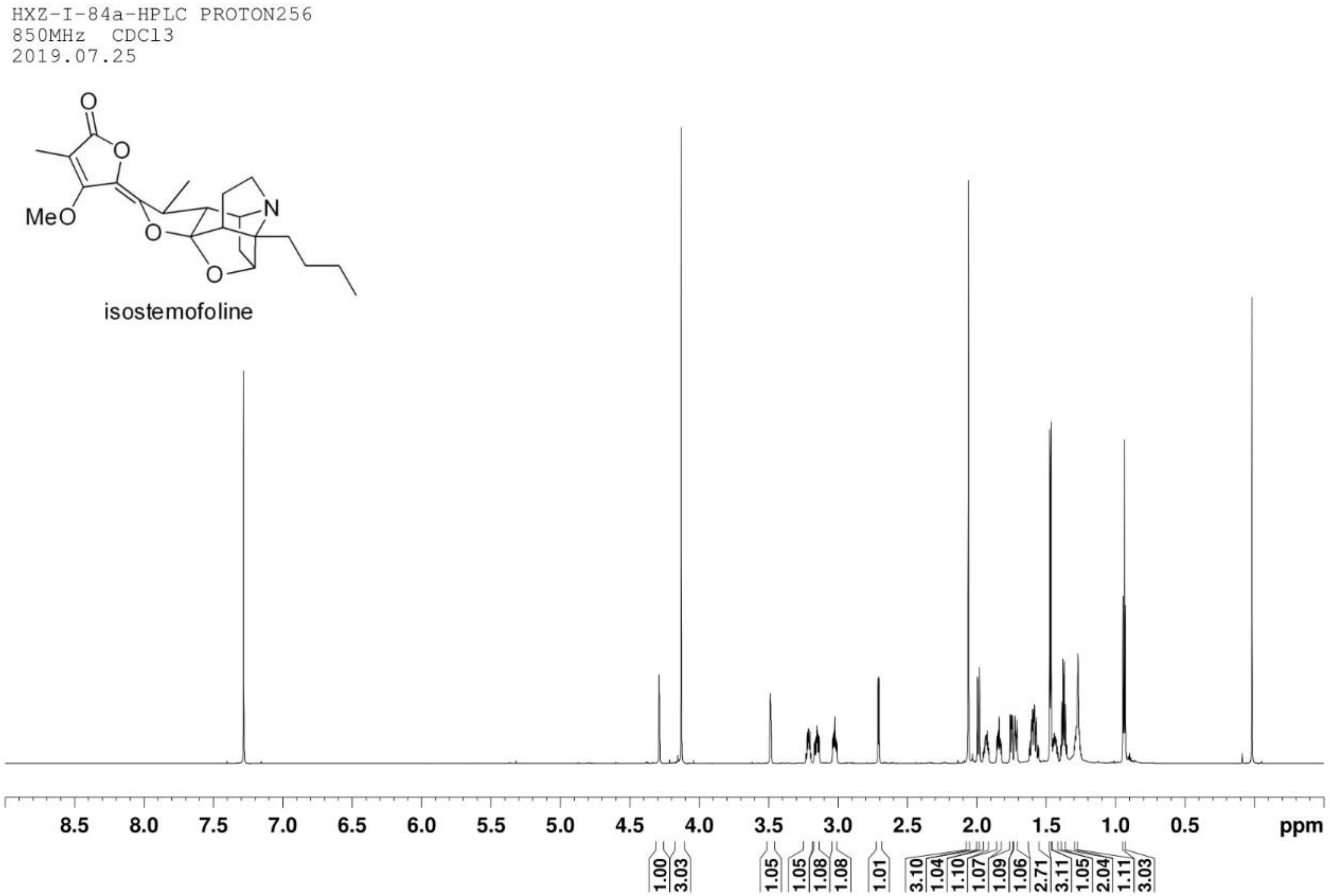

Supplementary Figure 66. ¹H NMR spectrum of compound isostemofoline (**2**)

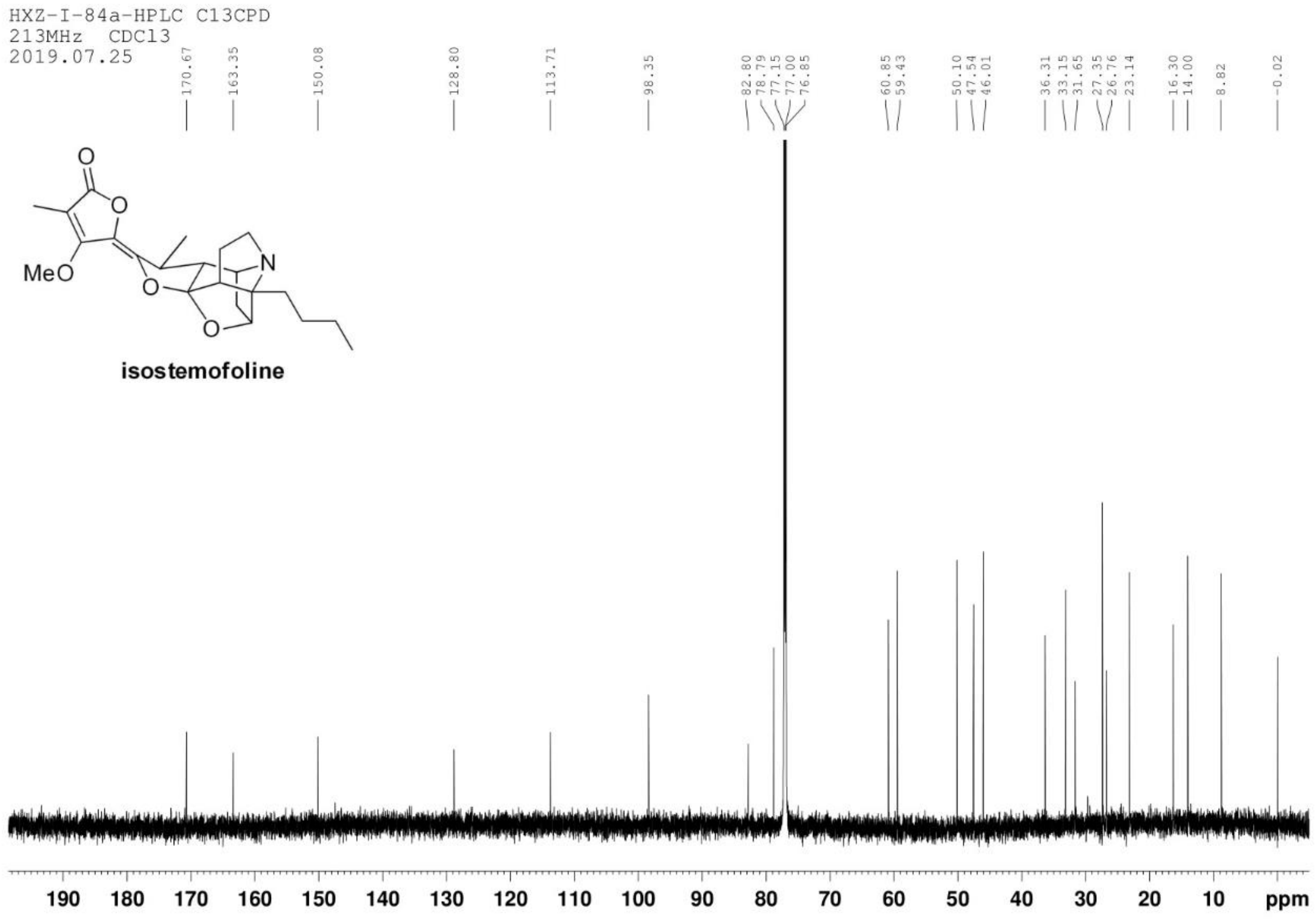

Supplementary Figure 67. ^{13}C NMR spectrum of compound isostemofoline (**2**)

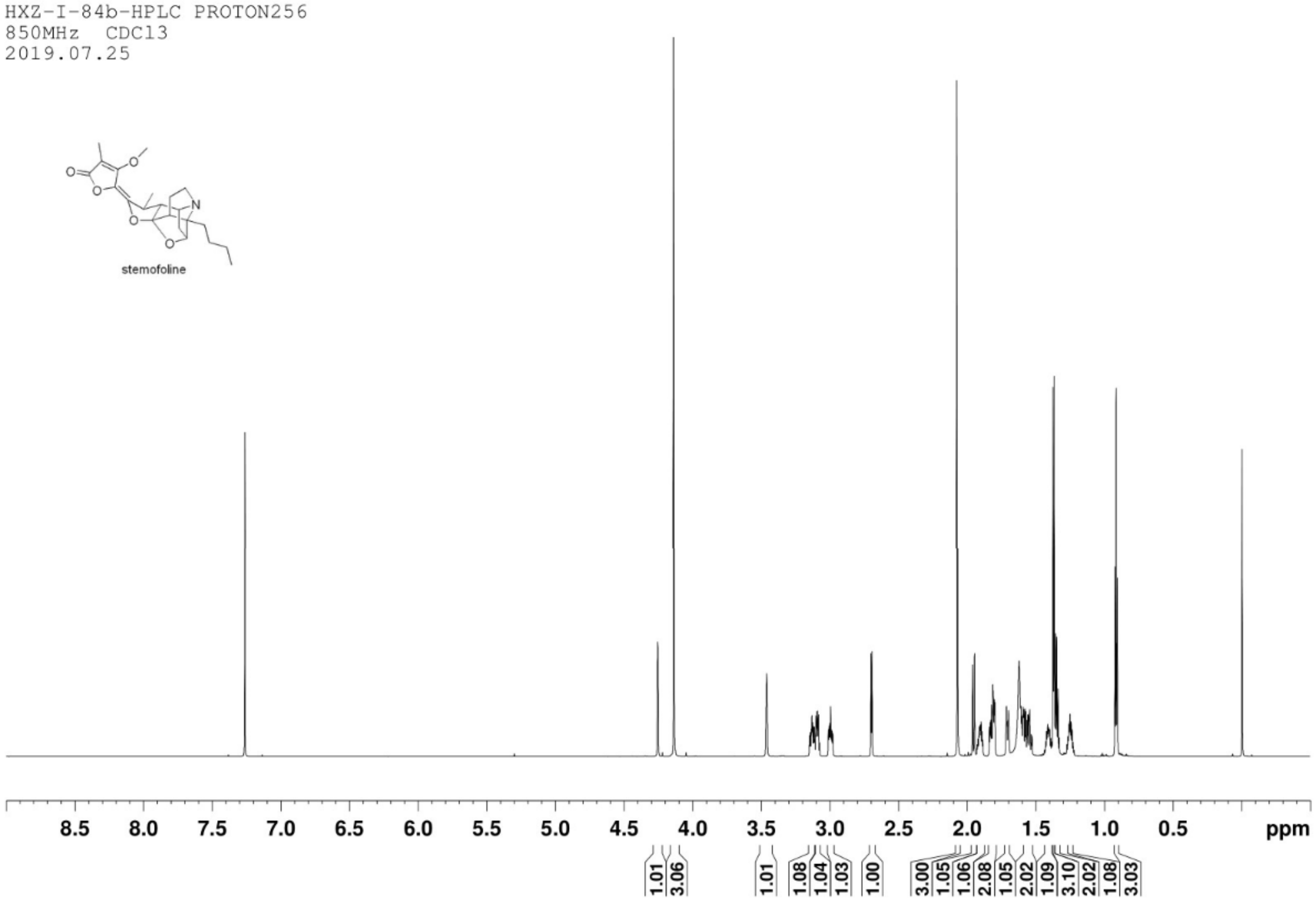

Supplementary Figure 68. ^{1}H NMR spectrum of compound stemofoline (**1**)

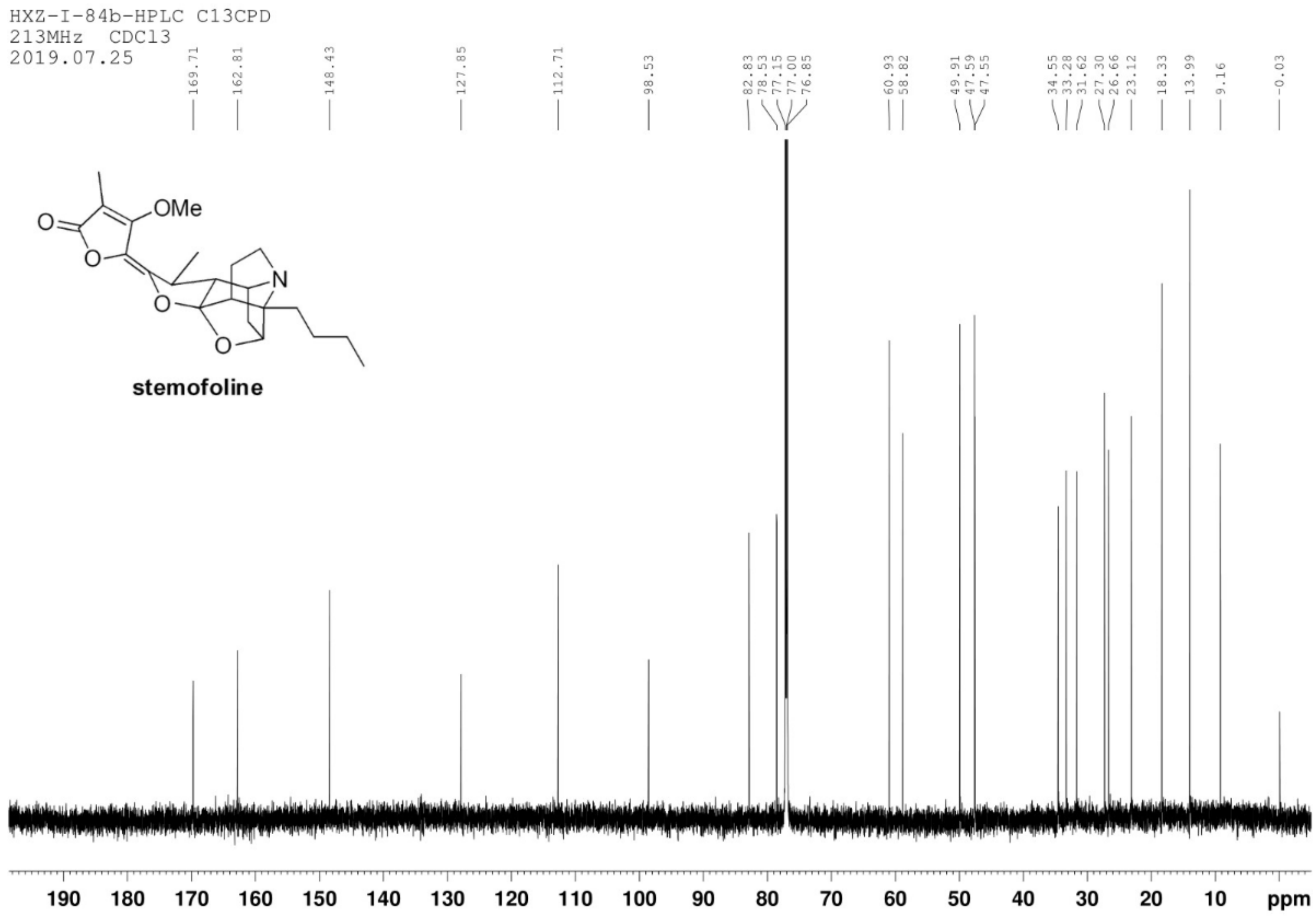

Supplementary Figure 69. ^{13}C NMR spectrum of compound stemofoline (**1**)

Supplementary References

(1) Huang, P.-Q. et al. Enantioselective total synthesis of (+)-methoxystemofoline and (+)-isomethoxystemofoline. *Chem. Commun.*, **51**, 4576–4578 (2015).

(2) Kende, A. S., Smalley, T. & Huang, H. Total synthesis of (±)-isostemofoline. *J. Am. Chem. Soc.* **121**, 7431–7432 (1999).

(3) Sastraruji, K. et al. Semisynthesis and acetylcholinesterase inhibitory activity of stemofoline alkaloids and analogues. *J. Nat. Prod.* **73**, 935–941 (2010).

(4) Thuring, J. J. F. et al. *N*-Phthaloylglycine-derived strigol analogues. Influence of the D-ting on seed germination activity of the parasitic weeds Striga hermonthica and Orobanche crenata. *J. Agric. Food Chem.* **45**, 2284–2290 (1997).

(5) Mungkornasawakul, P. et al. Phytochemical studies on Stemona burkillii prain: two new dihydrostemofoline alkaloids. *J. Nat. Prod.* **67**, 1740–1743 (2004).

(6) Sastraruji, K. et al. Structural revision of stemoburkilline from an *E*-alkene to a *Z*-alkene. *J. Nat. Prod.* **72**, 316–318 (2009).

(7) Irie, H. et al. The Crystal Structure of a New Alkaloid, Stemofoline, from *Stemona japonica*. *J. Chem. Soc., Chem. Commun*., 1066–1066 (1970).

(8) Seger, C. et al. Two pyrrolo[1,2-*a*]azepine type alkaloids from *Stemona collinsae* CRAIB: structure elucidations, relationship to asparagamine a, and a new biogenetic concept of their formation. *Chem. Biodivers.* **1**, 265–279 (2004).

(9) Chanmahasathien, W., Ampasavate, C., Greger, H. & Limtrakul, P. *Stemona* alkaloids, from traditional Thai medicine, increase chemosensitivity via P-glycoprotein-mediated multidrug resistance. *Phytomedicine* **18**, 199–204 (2011).
